# Complexation by γ-cyclodextrin as a way of improving anticancer potential of sumanene

**DOI:** 10.1038/s41598-024-78110-1

**Published:** 2024-11-07

**Authors:** Artur Kasprzak, Agnieszka Żuchowska, Hidehiro Sakurai

**Affiliations:** 1 Faculty of Chemistry, Warsaw University of Technology, Noakowskiego Str. 3, Warsaw, 00-664 Poland; 2 Division of Applied Chemistry, Graduate School of Engineering, Osaka University, 2-1 Yamadaoka, Suita, 565-0871 Osaka Japan; 3 Innovative Catalysis Science Division, Institute for Open and Transdisciplinary Research Initiatives (ICS-OTRI), Osaka University, Suita, 565-0871 Osaka Japan

**Keywords:** Supramolecular chemistry, Carbohydrate chemistry, Medicinal chemistry, Physical chemistry

## Abstract

Biological applications of sumanene *buckybowl* molecule have been widely discussed over the years yet remain still unexplored experimentally. On the other hand, creating cyclodextrin-containing supramolecular assemblies was demonstrated to be a powerful tool in terms of designing effective systems for medicinal chemistry purposes. Here, we show that sumanene molecule exclusively forms 1:1 host-guest complexes with γ-cyclodextrin (γCD) or (2-hydroxypropyl)-γ-cyclodextrin (HP-γCD), as revealed by extensive spectroscopic studies supported with density functional theory (DFT) computations. Based on our preliminary biological studies, we discovered that the formation of such complexes resulted in the improvement of anticancer properties of sumanene, expressed by high cell viabilities in vitro of healthy human mammary fibroblasts (HMF) together with low viabilities of human breast adenocarcinoma cells (MDA-MB-231). Improved pharmacokinetic (ADME-Tox) properties for sumanene@γCD and sumanene@HP-γCD complexes in comparison to native sumanene were also supported by *in sillico* modeling studies. This work provides the method how to focus the cytotoxic action of sumanene toward cancer cells using supramolecular assembly strategy, paving the way to the further exploration of biological properties of sumanene-containing supramolecular systems with bioactive features and applications of this *buckybowl* in general.

**Supplementary Information:**

The online version contains supplementary material available at 10.1038/s41598-024-78110-1.

## Introduction

**Fig. 1:**
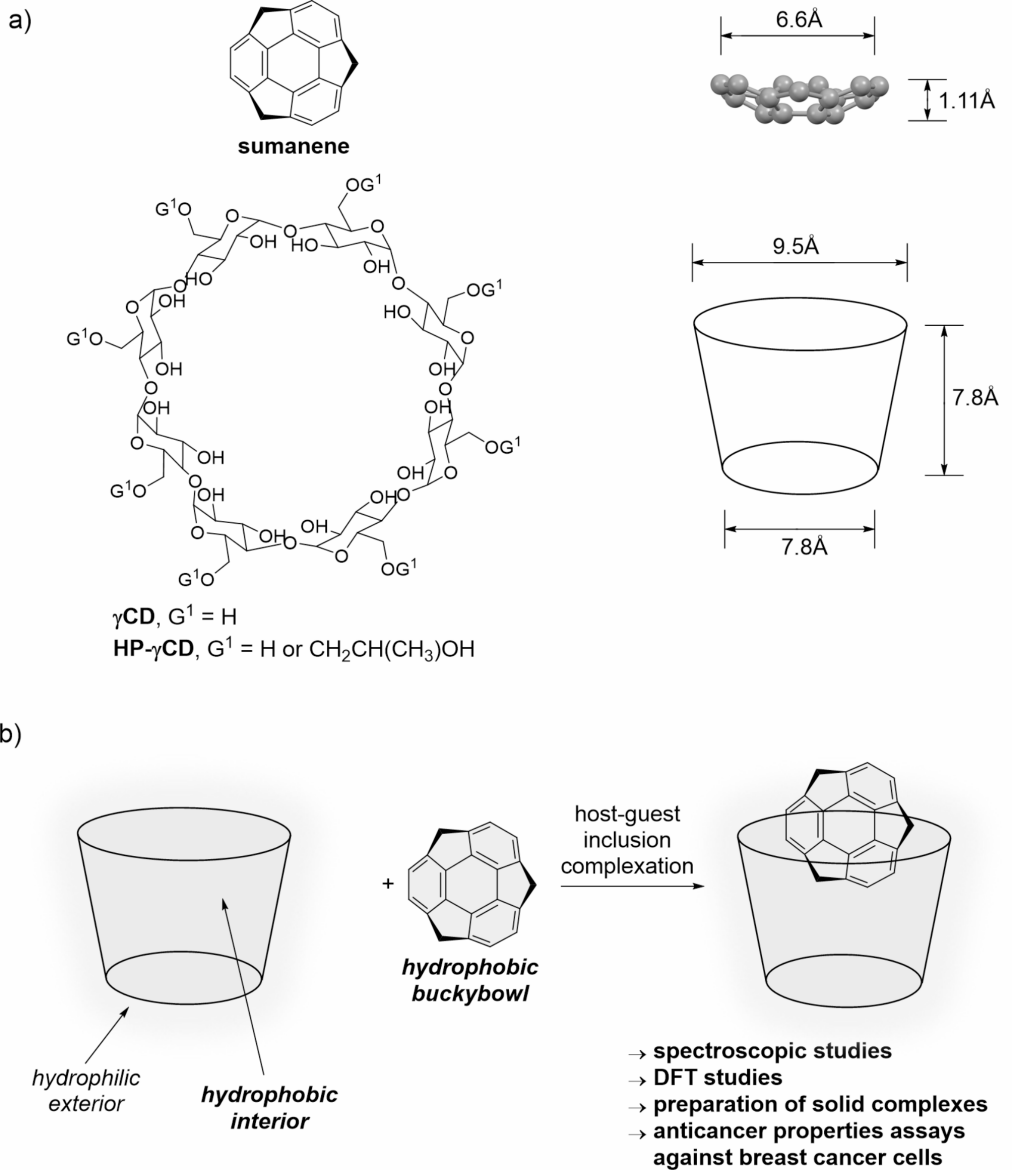
(**a**) Structures of sumanene (and representation of its bowl shape), γCD and HP-γCD (and representation of their cup shape), together with (**b**) graphical representation of the aims of this work.

Bowl-shaped molecules (*buckybowls*) are an emerging class of compounds for various applications^1–4^. Sumanene is an example of a *buckybowl* featuring many unique physicochemical properties. Since it is a bowl-shaped fragment of fullerene C_60_, it might be included in the family of compounds originating from carbon allotropes. Due to the significant curvature in its structure (bowl depth of 1.11Å, from the crystal structure^5^; Fig. 1a), sumanene shows not only attractive optical properties but also is a valuable building unit of many functional materials, including organized frameworks or capsules^6–9^. Since its first successful synthesis reported 21 years ago^10,11^, there has been a continuous interest in expanding the chemistry of this molecule from basic research to applied sciences. In the last five years, we witnessed the achievement of many important milestones in this light, such as the design of sumanene-based liquid crystals^12^, supramolecular polymers^13^, molecular receptors^14–16^, and crystalline dielectric materials^17–19^.

Despite these significant achievements within sumanene science, applications of this *buckybowl* molecule in medicinal chemistry remain uncovered. On the other hand, there are reports suggesting biological features of corannulene *buckybowl* (another fullerene fragment)^20,21^. Notably, the chemistry of corannulene is generally more explored than sumanene science since corannulene was isolated more than 30 years before sumanene. In fact, bio-related properties of *buckybowls* were widely discussed and anticipated over the years due to their origin from carbon allotropes, which feature many valuable possibilities in medicinal chemistry^22–24^. So far, this field for sumanene has been only studied theoretically by several groups, with computational results suggesting the potential of sumanene molecule in the delivery of drugs, such as 5-fluorouracil, methimazole or niraparib (recent works dated back to 2021–2023)^25–28^. Very recently (early 2024) our group reported on the application of ferrocenium-containing sumanenes as anticancer agents^29^. This work revealed possible anticancer effects of sumanene, mostly tuned by the presence of ferrocenium cation in the molecule. Generally, the cytotoxic action of sumanene can be expected, however, the still existing limitation of medicinal applications of sumanene could be mainly related to its polycyclic aromatic hydrocarbon (PAH) nature, which can be considered a drawback in terms of demonstrated possible toxicity of PAHs to humans^30,31^. Therefore, from the viewpoint of the anticancer potential of sumanene, there is not only a need to experimentally validate the toxicity of sumanene to cells but more importantly to design the system enabling the focus of biological action of sumanene on cancer cells with simultaneous high viability of normal cells.

In pursuit of opening yet uncovered medicinal chemistry applications of sumanene, as well as improving biocompatibility and cytotoxic action of this unique bowl-shaped molecule, here we report on the studies on the non-covalent supramolecular interactions between sumanene and γ-cyclodextrin (γCD) or (2-hydroxypropyl)-γ-cyclodextrin (HP-γCD), as well as preliminary evaluation of anticancer features of such attractive supramolecular assemblies (Fig. 1). Cyclodextrin based complexation, constitute flagship example of using supramolecular assembly toward improving, e.g., solubility, biocompatibility, bioavailability and cytotoxic action of drugs^32–34^. Our work was also inspired by the reports by M. C. Stuparu^35,36^ and Y. Zhao^37,38^ groups, which demonstrated the beneficial effects of γCD complexation on the biological properties of corannulene. Notably, it is known that host-guest complex formation with cyclodextrins is highly affected by the size of the guest molecule, thus, the possibility of sumanene complexation by γCD might be considered not obvious, since corannulene is shallower (bowl depth 0.875Å) and wider (8.3Å) than sumanene (1.1Å x 6.60Å). The results of our studies reported herein revealed that sumanene can exclusively form 1:1 inclusion complexes with γCD/HP-γCD with satisfactory binding parameters, as elucidated with comprehensive spectroscopic and computational (density functional theory, DFT) studies. Importantly, for the first time, we found that γCD/HP-γCD complexation has an effect on lowering adverse cytotoxicity of sumanene toward healthy cells together with improving cytotoxic action against cancer cells, as elucidated with in vitro studies with human mammary fibroblasts (HMF) and human breast adenocarcinoma cells (MDA-MB-231). The experimental results on the biological potential of sumanene@γCD and sumanene@HP-γCD complexes were also supported with *in sillico* modeling of pharmacokinetic (ADME-Tox) properties.

## Results and discussion

Sumanene and γCD/HP-γCD feature different solubility profiles. Sumanene is a hydrophobic bowl-shaped PAH and, thus, is practically water-insoluble, whereas γCD/HP-γCD are soluble in water. The water solubility of HP-γCD is higher in comparison to γCD^39–41^. The exterior of γCD/HP-γCD is hydrophilic, whereas its interior is hydrophobic (see graphical representation in Fig. 1b). To evaluate the possibility of host-guest inclusion complexes formation between sumanene and γCD/HP-γCD, we selected dimethylsulfoxide (DMSO) as the solvent for analyzes. While the solubility of sumanene in DMSO is highly limited, we concluded that this is not only a good solvent for γCD/HP-γCD, but also considering its water miscibility, there is a possibility to force the inclusion phenomenon by inducing solvophobic effects. The latter aspect can be considered important since the complexation phenomenon with cyclodextrins might be limited in water-free organic solutions^42–45^.

**Fig. 2:**
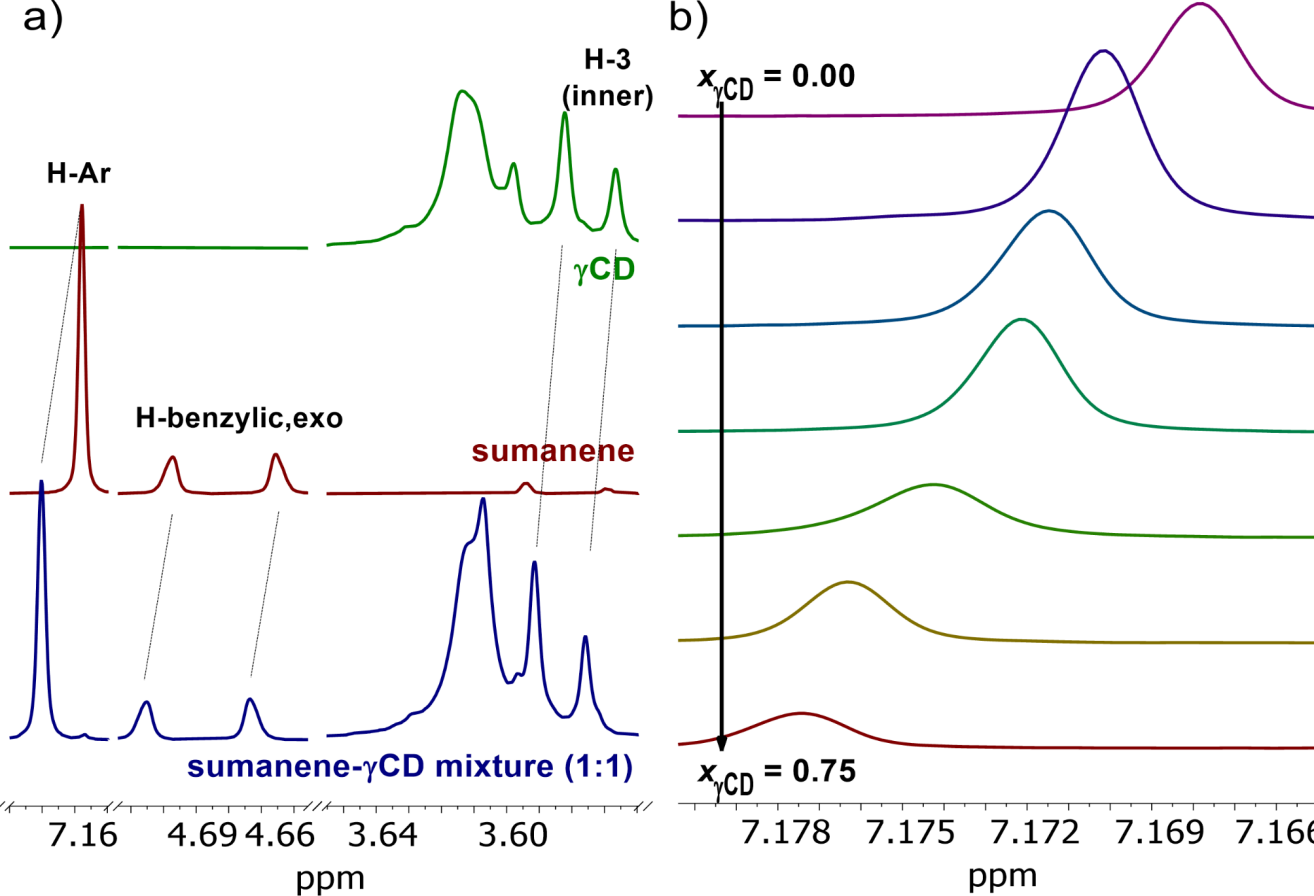
The selected results of ^1^H NMR (5 *vol*% D_2_O in DMSO-*d*_6_; *C*_sumenene_ = 3.15 mM, *C*_γCD_ = 3.15 mM; at 297.15 K) analyzes on the supramolecular interactions between sumanene and γCD: (**a**) selected key changes between the spectra of native molecules and their 1:1 mixture, (**b**) evolution of ^1^H NMR spectra (sumanene H_Ar_ region) regarding Job’s plot analyzes with the increasing *x*_γCD_ in the sample.

^1^H NMR spectroscopy analyses of the mixtures of sumanene and γCD/HP-γCD in deuterated DMSO (DMSO-*d*_6_) in the presence of deuterated water (D_2_O; 5 *vol*%) revealed the clear downfield shifts of H_Ar_ and H_benzylic_ from sumanene, as well as inner protons^45^ of γCD/HP-γCD (see Fig. 2a for spectra of sumanene and γCD interactions; refer to supporting information (SI), Section S2, for other spectra). No chemical shifts (d_H_) of OH-6, H-6 (-C**H**_2_-O**H** group), and H-2, H-4 (outer protons) of γCD were detected. It not only further suggested the inclusion nature of the interaction phenomenon, but also suggested sumanene docking into the γCD cavity through the wider side of the γCD cup, similar to the corannulene molecule^35,45^. Importantly, respective ^1^H NMR experiments with cyclodextrins featuring smaller cavities, namely α-cyclodextrin (αCD) and β-cyclodextrin (βCD), revealed no interactions with sumanene (refer to SI, Section S2 for spectra). It supported the specificity of the interaction of sumanene with γCD, demonstrating that the size of cyclodextrin’s cup (9.5Å x 7.8Å for γCD, Fig. 1a) plays an essential role in terms of the possibility of complex formation with sumanene. The stoichiometry of the system, estimated with the continuous variation method (Job’s plot)^46^, was found to be 1:1, based on the data for the downfield shifts of both H_Ar_ (Fig. 2b) and H_benzylic_ (SI, Section S2) from sumanene, as well as fluorescence spectroscopy analyzes (SI, Section S3).

^1^H-^1^H ROESY NMR experiment (DMSO-*d*_6_ + D_2_O) for the representative sumanene@γCD complex revealed mostly small intensity cross-peaks between H_benzylic-endo_ protons from sumanene and inner protons of γCD (refer to SI, Fig. S11), indicating inclusion of sumanene bowl inside γCD cup and the smallest distance of these protons to the inner protons of γCD^35,45^. This outcome also suggested that the sumanene molecule might not deeply penetrate the γCD cup, which is similar to the complexation behavior for methylcorannulene^35^. Notably, no such cross-peaks were detected when the spectrum of sumanene and γCD mixture was acquired in DMSO-*d*_6_ without the presence of D_2_O (refer to SI, Fig. S12).

^1^H DOSY NMR experiments revalued that this pseudo-2D NMR technique could be helpful in the analysis of non-covalent supramolecular interactions between *buckybowls* and cyclodextrins since different diffusion coefficient values for sumanene and γCD/HP-γCD in the complex sample in comparison to native molecules were found (refer to SI, Sections S1-S2 and Table S1, for spectra and data). It indicated dynamic, non-covalent interactions between the molecules in solution^47–49^. Approximate hydrodynamic radii (*r*_H, solv_) for the complexes were about 1.79–1.81 nm (calculated from the Stokes-Einstein Eq. 5^0a^; refer to SI, Section S1.2 for details of calculation). At 297.15 K, the estimated binding constant (*K*) value^50b–53a^ for sumanene@γCD complex equaled 53.10 M^− 1^ which corresponded to the Gibbs free energy (Δ*G*) value of − 9.81 kJ × mol^− 1^, whereas the respective values for sumanene@HP-γCD were 62.70 M^− 1^ and − 10.23 kJ × mol^− 1^. The respective values at 318.15 K were found to be 11.76 M^− 1^ (− 6.52 kJ × mol^− 1^) and 30.51 M^− 1^ (− 9.04 kJ × mol^− 1^), for sumanene@γCD and sumanene@HP-γCD, respectively, revealing the effect of increased temperature on lowering binding parameters for the studied complexes^53b-c^

Fluorescence spectroscopy titration experiments (DMSO: H_2_O = 2:1 *vol*/*vol*) revealed *turn-on* fluorescence behavior upon the addition of further portions (molar equivalents) of γCD/HP-γCD to a sumanene solution (refer to SI, Section S3, for the spectra). It further confirmed non-covalent interactions between molecules in the sample^54–56^. Notably, respective fluorescence spectroscopy titrations with αCD and βCD revealed no changes in the spectra of sumanene upon the addition of increasing molar equivalents of αCD and βCD, further supporting the specificity of the supramolecular interactions between sumanene and γCD/HP-γCD (refer to SI, Section S3, for the spectra). Binding constant (and Δ*G* in bracket) values for 1:1 complexes, estimated with the Benesi-Hildebrand method^57^, were 3.17 × 10^4^ M^− 1^ (− 25.60 kJ × mol^− 1^) and 1.05 × 10^5^ M^− 1^ (− 28.56 kJ × mol^− 1^) for sumanene@γCD and sumanene@HP-γCD, respectively (see summary of binding parameters in Table 1). These values are higher in comparison to the *K* estimated from ^1^H DOSY NMR experiments due to the significantly higher content of water in the sample in the case of spectrofluorimetric analyses. The 1:1 stoichiometry of the complexes and *K* values (4.4 ± 0.8 × 10^4^ M^− 1^ and 8.4 ± 2.2 × 10^4^ M^− 1^for sumanene@γCD and sumanene@HP-γCD, respectively) were also confirmed using global fitting to the 1:1 model using Bindfit (non-linear (direct) data treatment; refer to SI, Section S3, for the data)^58–60^. Notably, the *K* value for sumanene@γCD complex is ca. 10-fold higher in comparison to corannulene@γCD complex (3.99 × 10^3^), suggesting a more favorable inclusion of sumanene in γCD cup in comparison to corannulene molecule^35^. Additionally, for the representative sumanene@γCD complex, the spectrofluorimetric titration experiments were additionally performed in the DMSO: H_2_O = 4:1 *vol*/*vol* solvent system. These experiments further revealed that the two-fold increase in DMSO content in the sample lowered the binding parameters (ca. 1.5-fold in terms of *K*; see data in Table 1). This finding is in agreement with the above-discussed differences in *K* between spectrofluorimetric titration in DMSO: H_2_O = 2:1 *vol*/*vol* solvent system and ^1^H DOSY NMR studies in 5 *vol*% D_2_O in DMSO-*d*_6_).

Phase solubility diagrams, which enabled the estimation of the effect of the presence of γCD/HP-γCD in solution on the solubility of sumanene, featured typical behaviors for γCD and HP-γCD containing complexes. In the case of γCD, the initial increase in sumanene solubility was observed, followed by the quasi-plateau region and a final slight decrease in solubility (B_S_-type solubility curve; refer to SI, Section S4)^61^. It corresponded to the limited solubility of the formed complexes. On the other hand, HP-γCD featured a linear increase of sumanene solubility for increasing concentrations of HP-γCD (refer to SI, Section S4), following A_L_ classification by Higuchi and Connors^61^. Notably, the *K* value determined from phase solubility studies for interactions between sumanene and HP-γCD (9.86 × 10^4^ M^− 1^) was consistent with the respective value from fluorescence titration experiments (refer to SI, Section S1 for details of calculation).

**Table 1:** Summary of binding parameters (at 297.15 K) for sumanene@γCD and sumanene@HP-γCD inclusion complexes (stoichiometry 1:1).

Complex	Solvent	*K* [M^− 1^]	Δ*G* [kJ × mol^− 1^]
sumanene@γCD	DMSO-*d*_6_:D_2_O = 95:5 *vol*/*vol*	53.10^[a], [b]^	−9.81^[b]^
	DMSO: H_2_O = 2:1 *vol*/*vol*	3.17 × 10^4 [c], [d]^	−25.61
	DMSO: H_2_O = 4:1 *vol*/*vol*	2.09 × 10^4 [c], [e]^	−24.04
sumanene@HP-γCD	DMSO-*d*_6_:D_2_O = 95:5 *vol*/*vol*	62.70^[a], [f]^	−10.23^[e]^
	DMSO: H_2_O = 2:1 *vol*/*vol*	1.05 × 10^5 [c], [g]^ 9.86 × 10^4 [h]^	−28.56 −28.41

^[a]^Estimated from ^1^H DOSY NMR. ^[b]^11.76 M^− 1^ (− 6.52 kJ × mol^− 1^) at 318.15 K. ^[c]^Estimated from fluorescence spectroscopy with the Benesi-Hildebrand method. ^[d]^4.4 ± 0.8 × 10^4^ M^− 1^ from global fitting to the 1:1 model using Bindfit. ^[e]^3.2 ± 0.3 × 10^4^ M^− 1^ from global fitting to the 1:1 model using Bindfit. ^[f]^30.51 M^− 1^ (− 9.04 kJ × mol^− 1^) at 318.15 K. ^[g]^8.4 ± 2.2 × 10^4^ M^− 1^ from global fitting to the 1:1 model using Bindfit. ^[h]^Estimated from phase solubility studies.

**Fig. 3:**
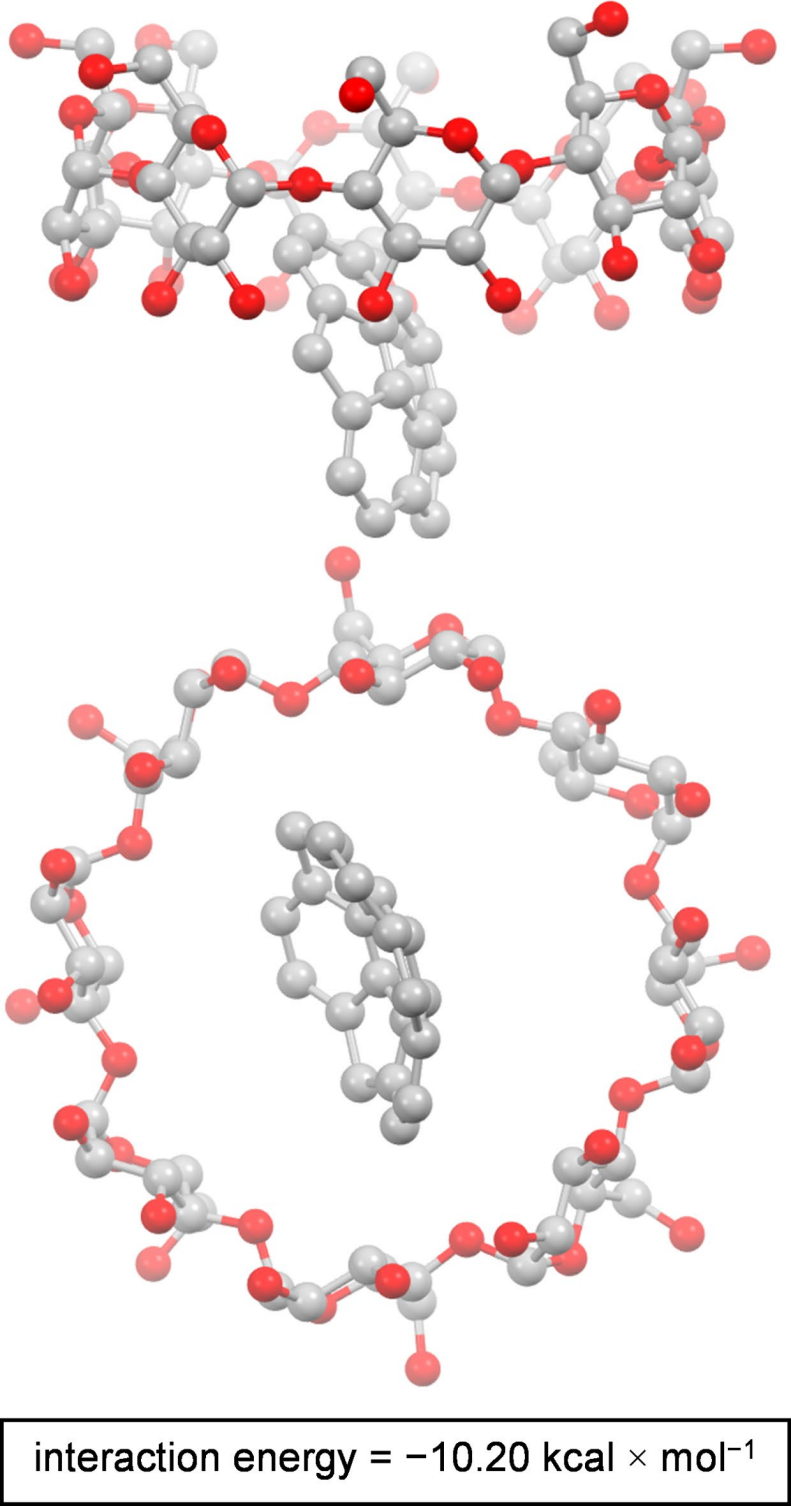
DFT optimized (B3LYP/6–31g(d, p)) structure of exampled sumanene@γCD complex with views presented from two different perspectives (hydrogen atoms are omitted for the clarity of the image). Interaction energy (Δ*G*) for this complex arrangement (in a gas phase) is also provided.

Density functional theory (DFT) computations, performed using Gaussian software^62^ for 1:1 sumanene@γCD complex with B3LYP functional^63^ and 6–31g(d, p) basis set^64^ (in a gas phase), revealed that interaction of sumanene with inner part of γCD cup is more favorable than interaction with γCD’s outer part, further supporting host-guest inclusion complexation phenomenon (refer to SI, Section S5 for full details on DFT computations on five representative complex arrangements). Calculated interaction energies (Δ*G*) for various inclusion complex orientations have negative values ranging from − 10.20 to − 5.50 kcal × mol^− 1^, suggesting spontaneous complexation reaction (one of DFT computed complex structures is presented in Fig. 3). On the other hand, the respective value for the non-inclusion complex is positive (+ 0.41 kcal × mol^− 1^), what points on the thermodynamically unfavorable direction of the process. Notably, the interaction energies for the inclusion complexes in which the sumanene molecule was vertically oriented with regard to γCD cup were found to be higher (− 10.20 to − 10.15 kcal × mol^− 1^) in comparison to the respective values for complexes in which the sumanene molecule was horizontally oriented (− 5.60 to − 5.50 kcal × mol^− 1^; refer to SI, Table S2 for data). We believe this trend is associated with the width of the sumanene molecule (ca. 6.6Å) which is only ca. 2.9Å lower than the width of the wider site of γCD cup (9.5Å; refer to Fig. 1a for the sizes of sumanene and γCD). On the other hand, the value of sumanene’s bowl depth (ca. 1.11Å) is ca. 8Å lower, which enables better fitting between molecules toward the formation of the inclusion complex.

Next, the samples of solid sumanene@γCD and sumanene@HP-γCD inclusion complexes were prepared using the environmentally friendly mechanochemical method, namely water-assisted grinding with RETSCH MM 400 ball mill (refer to SI, Section S1, for the full experimental details)^65–67^. In brief, sumanene (0.043 mmol) and γCD/HP-γCD (0.043 mmol) were placed in a grinding jar with a few grinding balls, followed by the addition of 10 mL of distilled water and intense grinding (2 h, 30 Hz). The target complexes were effectively obtained (87% and 60% yield for sumanene@γCD and sumanene@HP-γCD, respectively) by means of filtration and washing off with proper, cold solvents.

^1^H and {^1^H}^13^C NMR spectra in DMSO-*d*_6_ of the as-prepared complexes revealed the presence of signals coming from both sumanene^68^ and γCD/HP-γCD^45^ (refer to SI, Section S6, for the spectra). For representative sumanene@γCD complex, the 1:1 (sumanene: γCD) composition of the sample was supported with ^1^H NMR and UV-vis spectroscopy analyzes (refer to SI, Section S6, for the spectra and data), whilst at least 97% purity was supported with ^1^H quantitative NMR (qNMR) experiment^69–71^. Additionally, ESI-MS (TOF) experiment with representative sumanene@γCD inclusion complex further verified that sumanene forms a 1:1 complex with γCD, since the experimental and simulated isotopic patters well matched (refer to SI, Section S6, for the spectrum).

**Fig. 4:**
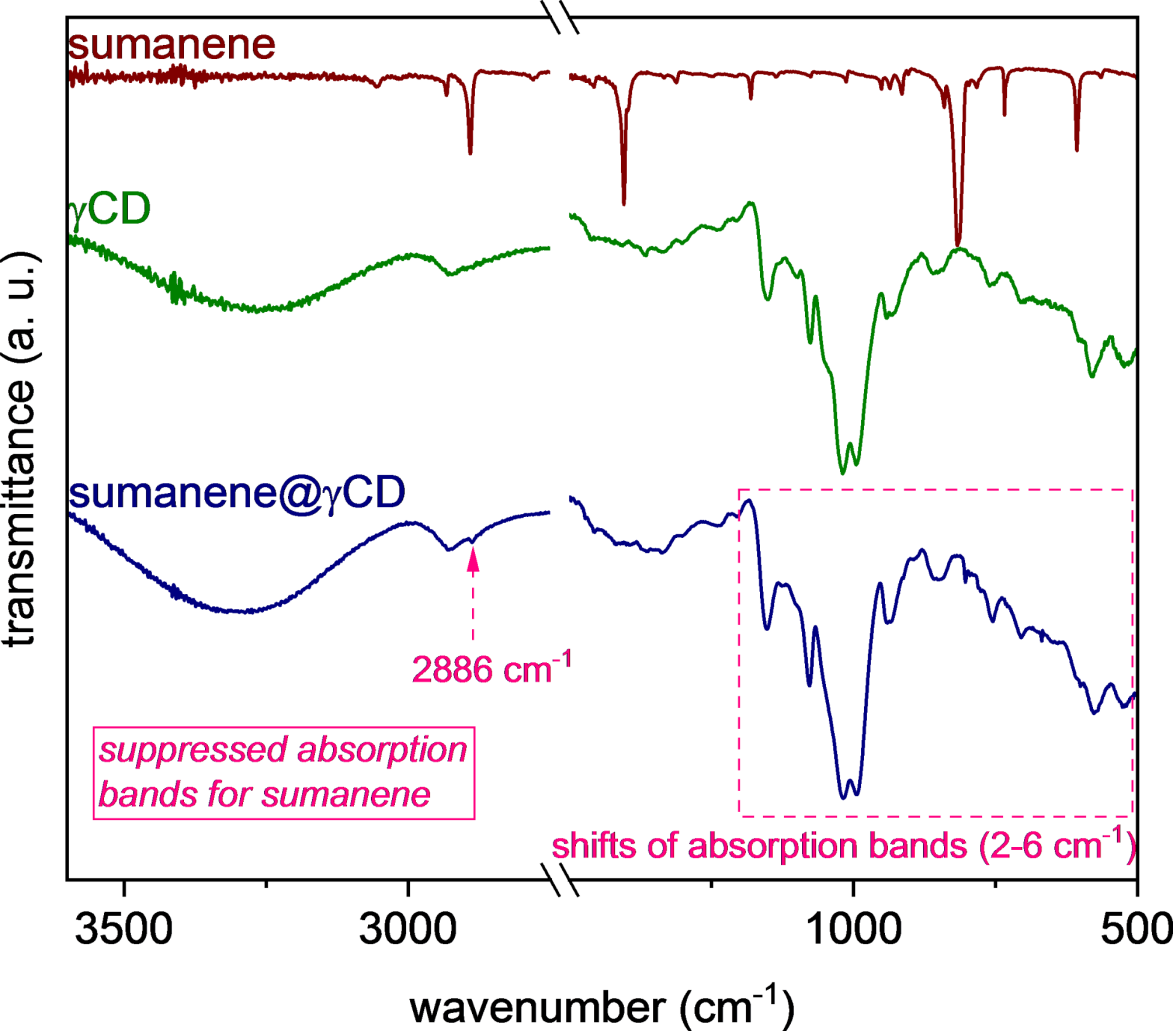
FT-IR (ATR) spectra of sumanene, γCD, and mechanochemically-prepared sumanene@γCD inclusion complex. The key features of the FT-IR spectrum of sumanene@γCD are marked in pink.

Finally, Fourier-transform infrared spectroscopy (FT-IR) were acquired for the solid inclusion complex samples. The absorption bands coming from the sumanene skeleton were suppressed in the FT-IR spectrum of the complex, which is a typical behavior for the cyclodextrin-based inclusion complexes (see Fig. 4 for the stacked FT-IR spectra regarding sumanene@γCD complex; refer to SI, Section S6, for other FT-IR spectra)^72–74^. Additionally, slight shifts (2–6 cm^− 1^) of the absorption bands coming from the vibrations of structural moieties within γCD/HP-γCD skeleton were observed, mostly in the 1200 –550 cm^− 1^ region. Notably, in the FT-IR spectrum of sumanene@γCD, the presence of an additional low-intensity absorption band at 2886 cm^− 1^ was observed, which could be ascribed to aromatic/benzylic C-H stretching vibrations coming from sumanene^75,76^. It further suggested that some structural parts of sumanene might not deeply penetrate the γCD cup, which can be considered consistent with the outcome from the ^1^H-^1^H ROESY NMR experiment.

Having established host-guest supramolecular interactions between sumanene and γCD/HP-γCD, as well as successful synthesizing solid inclusion complexes, our attention in the final stage of our research program focused on the preliminary biological evaluation of formed supramolecular assemblies in order to estimate their possible anticancer potential. Breast cancer has been the cause of millions of deaths over the years, with more than half a million each year^77,78^. Taking this into account, cytotoxicity studies with sumanene@γCD and sumanene@HP-γCD complexes were performed in vitro with human breast adenocarcinoma cells (MDA-MB-231). Respective studies were also performed with healthy human mammary fibroblasts (HMF) to estimate the specificity of cytotoxic action of considered supramolecular assemblies.

**Fig. 5:**
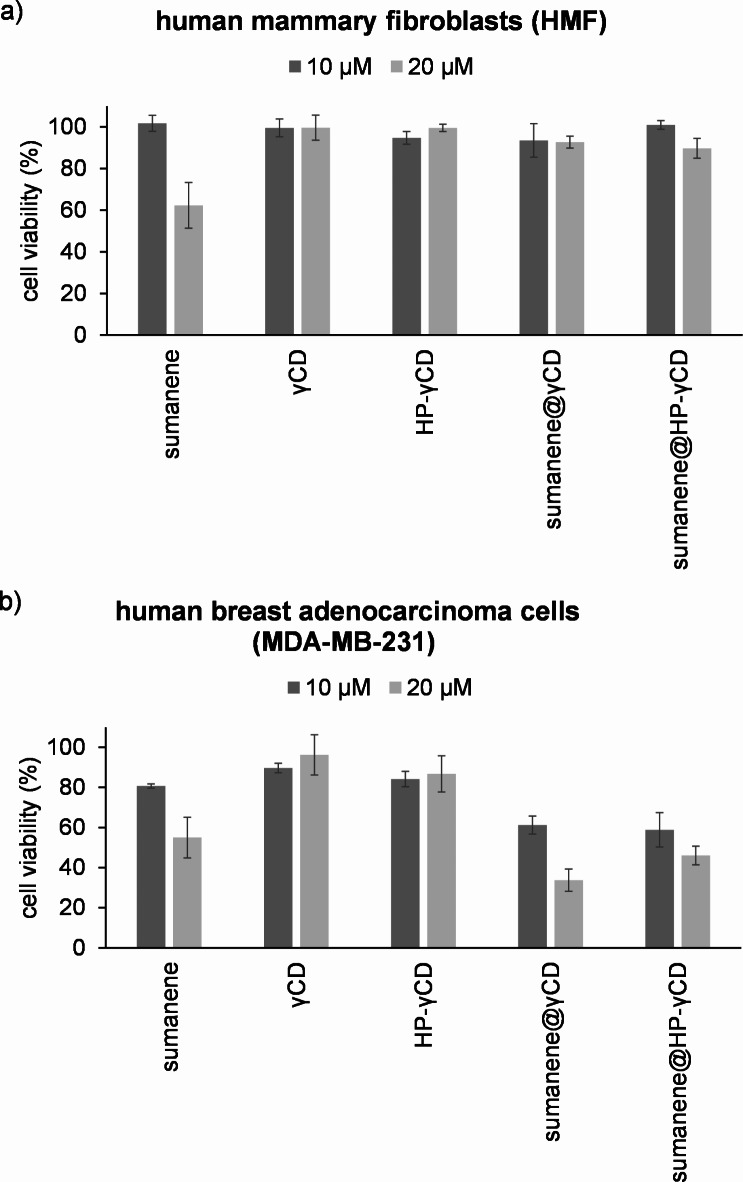
Cell viabilities (24 h) after treatment with sumanene, γCD, HP-γCD, sumanene@γCD inclusion complex and sumanene@HP-γCD inclusion complex: (**a**) human mammary fibroblasts (HMF, normal cells), (**b**) human breast adenocarcinoma cells (MDA-MB-231 cells).

Figure 5 shows the results of biological assays. The compounds were tested at two concentrations, namely 10 µM and 20 µM, and the incubation time was 24 h. Generally speaking, cell viabilities were lower for the higher concentration. In the used model, at 20 µM, sumanene molecule was found to feature similar cytotoxicity for both healthy cells (HMF; cell viability 62%) and breast cancer cells (MDA-MB-231; cell viability 57%), whereas for 10 µM slightly higher cytotoxicity against cancer cells was observed than for the normal cells, standing for some selective anticancer action of sumanene (cell viability 84% for cancer cells *versus* 98% for normal cells). γCD and HP-γCD were found to be nontoxic for healthy cells, which is in agreement with their biocompatible nature^32–34^, whereas only slight toxicity was concluded for cancer cells. On the contrary, sumanene@γCD and sumanene@HP-γCD complexes featured improved anticancer potential. For both concentrations of these supramolecular assemblies, significantly higher cell viabilities of normal cells were found in comparison to cancer cells (difference of ca. 32–42% and ca. 45–60% for concertation 10 µM and 20 µM, respectively). It means that sumanene@γCD and sumanene@HP-γCD complexes exhibited good selectivity of anticancer action, especially beneficial at the concentration of 20 µM. Specifically for the concentration of 20 µM, viability of normal (HMF) and cancer (MDA-MB-231) cells for sumanene@γCD were 93% and 33%, respectively, whereas for sumanene@HP-γCD these values were 90% and 46%, respectively. Notably, the anticancer effect for sumanene@γCD and sumanene@HP-γCD was found to be improved in comparison to native sumanene, elucidated by ca. 1.4- fold lower MDA-MB-231 cancer cell viabilities. It was also followed by the beneficial, ca. 1.5-fold higher viabilities of HMF normal cells in comparison to native sumanene.

Noteworthy, slightly better selectivity of anticancer action toward MDA-MB-231 cancer cells among all tested compounds together with high viabilities of HMF normal cells could be concluded for sumanene@γCD than for sumanene@HP-γCD. It could be explained by the plausible existence of the Warburg effect (increased glucose uptake by cancer cells)^79–81^ for this system related to the presence of repeating glucose units in γCD (higher content of unfunctionalized glucose units for this macrocycle in comparison to HP-γCD). Additionally, generally speaking, the existence of the Warburg effect for the studied supramolecular assemblies might be considered the origin of their selective toxicity to cancer cells and low toxicity to healthy cells.

Finally, *in sillico* modeling of pharmacokinetic (ADME-Tox) properties for sumanene@γCD and sumanene@HP-γCD complexes together with the comparison with the respective properties for native sumanene was performed with the usage of pkCSM^82–84^. Notably, *in sillico* methods have a continuously growing importance in the light of the preliminary evaluation of the medicinal potential of novel drug candidates^85,86^. In general, our *in sillico* analyses revealed significantly more beneficial ADME-Tox properties for sumanene@γCD and sumanene@HP-γCD complexes in comparison to native sumanene (refer to SI, Section S7, for all the modeled parameters). Briefly, sumanene@γCD and sumanene@HP-γCD inclusion complexes featured not only improved water solubility and log P value but also in terms of modulation of P-glycoprotein transport (absorption parameter). γCD or HP-γCD complexation also influenced the predicted permeability (distribution parameter) and metabolism factors of the sumanene in terms of its biological action, as well as improved total clearance (excretion parameter). These findings supported improved bioavailability for the inclusion complexes, observed during in vitro cytotoxicity studies. Predicted values for toxicity (Tox) parameters for sumanene@γCD and sumanene@HP-γCD complexes were also improved, elucidated by, e.g., lack of hepatotoxicity or higher maximal tolerated dose in humans in comparison to sumanene. Notably, in comparison to sumanene for which modeled Minnow toxicity suggested high acute toxicity for this molecule (predicted value, log mM = − 2.012), low acute toxicity was predicted for sumanene@γCD and sumanene@HP-γCD complexes (predicted positive values, at the level of log mM = ca. 40). Lastly, in general, sumanene@γCD and sumanene@HP-γCD complexes featured relatively similar values of modeled pharmacokinetic (ADME-Tox) parameters. Sumanene@HP-γCD complexes featured slightly lower Blood-Brain Barrier (BBB) and central nervous system (CNS) permeability, slightly higher total clearance and Minnow toxicity values, together with lower predicted value of lowest observed adverse effect level dose (LOAEL; Oral Rat Chronic Toxicity parameter).

## Conclusions

In conclusion, this work introduced a new sumanene *buckybowl* containing supramolecular assemblies based on the host-guest complexation with γ-cyclodextrin (γCD) or (2-hydroxypropyl)-γ-cyclodextrin (HP-γCD). Binding constant values for such 1:1 inclusion complexes are on the satisfactory level (10^4^ to 10^5^ M^− 1^ in DMSO: H_2_O mixtures, 2:1 *vol*/*vol*). On the basis of our preliminary biological studies, we demonstrated that complexation by γCD/HP-γCD have a beneficial effect on cytotoxic properties of sumanene, elucidated by both the improvement of biocompatibility of this bowl-shaped molecule and the enhanced cytotoxic action toward cancer cells, as elucidated by in vitro studies with human mammary fibroblasts (HMF) and human breast adenocarcinoma cells (MDA-MB-231). Sumanene@γCD complex feature improved cytotoxic properties in comparison to sumanene@HP-γCD complex, plausibly due to the effective existence of the Warburg effect. *In sillico* modeling of pharmacokinetic (ADME-Tox) properties further supported the benefits of γCD or HP-γCD complexation of sumanene toward improving pharmacokinetic properties of this *buckybowl*. Taking into account the biocompatibility of the designed systems together with the targeted cytotoxic action of sumanene *buckybowl* against cancer cells, we believe this work opens new avenues for yet unexplored field of medicinal applications of sumanene and its supramolecular assemblies, both from the viewpoint of fundamental research within this new area of sumanene science and its future bio-applications.

## Methods

The Supporting Information (SI) contains the following data: Materials and methods, experimental procedures, spectra, data on the supramolecular interactions between sumanene and γCD/HP-γCD, DFT computational details, characterization of solid complexes, data on *in sillico* modeling of pharmacokinetic (ADME-Tox) properties. The following additional references were cited within the SI^87–92^.

## Electronic supplementary material

Below is the link to the electronic supplementary material.

Supplementary Material 1

## Data Availability

The data supporting this article have been included as part of the supporting information.
