# Supplementary material for: Complexation by γ-cyclodextrin as a way of improving anticancer potential of sumanene
Source: Sci Rep. 2024 Nov 7;14:27158. doi: 10.1038/s41598-024-78110-1 (PMC11543856; doi:10.1038/s41598-024-78110-1)
Supplement: Supplementary file 1 — Supplementary Material 1 [file 41598_2024_78110_MOESM1_ESM.pdf]

SUPPORTING INFORMATION (SI) FOR

Complexation by γ-Cyclodextrin as a Way of Improving Anticancer Potential of Sumanene

Artur Kasprzak[a]*, Agnieszka Żuchowska[a], Hidehiro Sakurai[b,c]

[a] Faculty of Chemistry, Warsaw University of Technology, Noakowskiego Str. 3, 00-664 Warsaw, Poland

* Corresponding author e-mail: artur.kasprzak@pw.edu.pl (A.K.)

[b] Division of Applied Chemistry, Graduate School of Engineering, Osaka University, 2-1 Yamadaoka, Suita, 565-0871 Osaka, Japan

[c] Innovative Catalysis Science Division, Institute for Open and Transdisciplinary Research Initiatives (ICS-OTRI), Osaka University, Suita, Osaka 565-0871, Japan

Table of contents

S1. Materials and methods 2
S2. NMR analyzes on the supramolecular interactions 7
S3. Fluorescence spectroscopy analyzes on the supramolecular interactions 20
S4. Phase solubility studies on the supramolecular interactions 30
S5. DFT computations 34
S6. Analyzes of mechanochemically-obtained inclusion complexes 62
S7. *In sillico* modeling of pharmacokinetic (ADME-Tox) properties 69
S8. Supporting references 71

S1. Materials and methods

S1.1. Materials

Materials. Chemical reagents and solvents were of the highest possible purity and were commercially purchased and purified according to the standard methods, if necessary. Sumanene was synthesized following the literature procedure.[1] Following cyclodextrins were used (Merck catalog numbers are provided): γ-cyclodextrin (γCD; Merck no. C4892), (2-hydroxypropyl)-γ-cyclodextrin (HP-γCD; Merck no. 390704), α-cyclodextrin (αCD; Merck no. C4642), β-cyclodextrin (βCD; Merck no. C4767).

S.1.2. Spectroscopic experiments on supramolecular interactions

The NMR experiments were carried out using a JEOL 600 MHz spectrometer (^{1}H NMR and {^{1}H}^{13}C NMR) equipped with a multinuclear z-gradient inverse probe head or Varian VNMRS 500 MHz spectrometer (^{1}H DOSY NMR and ^{1}H-^{1}H ROESY NMR) equipped with a multinuclear z-gradient inverse probe head. ^{1}H NMR, {^{1}H}^{13}C NMR and ^{1}H-^{1}H ROESY NMR spectra were recorded at 297.15 K. Standard 5 mm NMR tubes were used. ^{1}H NMR (δ_H) and {^{1}H}^{13}C NMR (δ_C) chemical shifts were reported in parts per million (ppm) relative to the solvent signal, *i.e.*, DMSO-d_6, δ_H (residual DMSO) 2.50 ppm, δ_C (residual DMSO) 39.52 ppm. ^{1}H DOSY (Diffusion Ordered SpectroscopY) experiments were performed at 297.15 K or 318.15 K (temperature indicated in the figure caption) using a stimulated echo sequence incorporating bipolar gradient pulses[2] and with convection compensation.[3] The gradient strength was logarithmically incremented in 15 steps from 25% up to 95% of the maximum gradient strength. ^{1}H NMR spectra were analyzed with the MestReNova v12.0 software (Mestrelab Research S.L), whereas ^{1}H DOSY NMR spectra were analyzed with the specialized DOSYToolbox[4] software.

UV-vis spectra were recorded with a WVR UV-1600PC spectrometer, with a spectral resolution of 2 cm^{-1} (297.15 K). For the UV-Vis measurements, the wavelengths for the absorption maxima λ_{max} were reported in nm.

Fluorescence spectra were recorded with a HITACHI F-7100 FL spectrometer; parameters for the spectra acquisition: scan speed: 1200 nm/min, delay: 0.0 s, EX slit: 5.0 nm, EM slit: 5.0 nm, PMT voltage: 400 V, excitation wavelength 280 nm (297.15 K). The wavelengths for the emission maxima (λ_{em}) were reported in nm.

^{1}H NMR analyses on the supramolecular interactions were performed in DMSO-d_6:D_2O = 95:5 *vol*/*vol* solvent system as follows. At first, DMSO-d_6 solutions (0.855 mL) containing (*i*) sumanene (0.00284 mol), (*ii*) γCD/HP-γCD (0.00284 mol), or (*iii*) sumanene (0.00284 mol) and γCD/HP-γCD (0.00284 mol), were prepared and well mixed in NMR tubes. Next, D_2O (0.045 mL) was added to these solutions and the contents of the NMR tubes were well mixed (no precipitation occurred in the samples, clear solution was obtained). The final sample volume was 0.900 mL, and the final concentration of sumanene/γCD/HP-γCD (contents depended on the sample) was 3.15

mM. It should be noted that this volume of D_2O was one of the maximum values that did not cause the precipitation of sumanene in an NMR tube.

Fluorescence spectra titrations on the supramolecular interactions were performed in the DMSO:H_2O = 2:1 *vol*/*vol* solvent system as follows. To a solution of sumanene (0.02 mM; 3 mL; DMSO:H_2O = 2:1 *vol*/*vol*) further portions of γCD/HP-γCD of stock solutions (1.50 mM or 20.00 mM; DMSO:H_2O = 2:1 *vol*/*vol*;) were added to reach given sumanene-to-γCD/HP-γCD molar ratio (from 0 equiv. to 52 equiv. of γCD/HP-γCD were added). Before the addition of γCD/HP-γCD, as well as after the addition of each portion of γCD/HP-γCD, the sample in the spectrophotometer cuvette was well mixed with magnetic stirring bar and a magnetic stirrer (1300 rpm, *ca*. 60 sec. of mixing; no precipitation occurred in the samples, clear solution was obtained each time). Fluorescence spectra were measured using λ_{ex} = 280 nm. Data were analyzed for λ_{em} = 379 nm. The association constant (*K*) value was estimated using a Benesi-Hildebrand method[5], namely from the linear $1/\Delta I = f(1/C_{\gamma CD/HP\text{-}\gamma CD})$ plot, where ΔI is the difference between fluorescence intensity for sumanene sample and fluorescence intensity of the sample after addition of given molar equiv. of γCD/HP-γCD, $C_{\gamma CD/HP\text{-}\gamma CD}$ is the concentration of γCD/HP-γCD in the sample for the given molar equiv. The values of *K* were taken as the intercept/slope ratio. Stoichiometry of the complexes, as well as K values, were also studied using a global fitting to 1:1 model (non-linear (direct) data treatment) with Bindfit.[6–8] For the representative sumanene@γCD complex, the respective spectrofluorimetric titration experiment was additionally performed in the DMSO:H_2O = 4:1 *vol*/*vol* system.

Fluorescence spectroscopy Job's plot[9,10] analyzes on the supramolecular interactions were performed in DMSO-d_6:D_2O = 2:1 *vol*/*vol* solvent system with samples keeping the total sum of moles of sumanene and γCD/HP-γCD ($n_{sumanene} + n_{\gamma CD/HP\text{-}\gamma CD}$) of 0.00000024 mol (total volume of the each sample was 4.0 mL; initial concentration of sumanene sample without γCD/HP-γCD added was 0.06 mM; no precipitation occurred in the samples, clear solution was obtained in the each case). The samples featured the following molar fractions of γCD/HP-γCD ($x_{\gamma CD/HP\text{-}\gamma CD}$): 0.00, 0.20, 0.25, 0.33, 0.50, 0.67 and 0.75.

^{1}H NMR Job's plot analyses on the supramolecular interactions were performed in DMSO-d_6:D_2O = 95:5 *vol*/*vol* solvent system with samples keeping the total sum of moles of sumanene and γCD ($n_{sumanene} + n_{\gamma CD/HP\text{-}\gamma CD}$) of 0.00284 mol (total volume of each sample was 0.9 mL; no precipitation occurred in the samples, the clear solution was obtained in the each case). The samples featured the following molar fractions of γCD ($x_{\gamma CD/HP\text{-}\gamma CD}$): 0.00, 0.20, 0.25, 0.33, 0.50, 0.67 and 0.75.

The values of association constant (*K*) from ^{1}H DOSY NMR experiments were estimated based on the literature method.[11–14a] All the diffusion coefficient values (*D*) were provided in m^2/s, and were viscosity corrected as follows using the following equation: $D_{cor} = D_{meas} \cdot (D_{solvent(ref)} \cdot D_{solvent(obs)}^{-1})$, where D_{cor} is a viscosity-corrected diffusion coefficient value for the molecule, D_{meas} is a diffusion coefficient for each

molecule taken from the ^{1}H DOSY NMR spectrum, $D_{solvent(ref)}$ is a diffusion coefficient for the solvent in pure solution (at 297.15 K: $4.960\cdot10^{-10}$ m^2/s, value taken from our previous studies[12]; at 318.15 K: $7.624\cdot10^{-10}$ m^2/s, the spectrum was measured additionally), and $D_{solvent(obs)}$ is diffusion coefficient observed for the solvent in the given sample. For the determination of K, at first, the molar fraction of the complexed guest (x_b) from ^{1}H DOSY NMR analyses was calculated using the following equation: $x_b = (D_{free} - D_{obs}) \cdot [(D_{free} - D_{bound})^{-1}]$, where D_{free} stands for a diffusion coefficient value for the native sumanene, D_{obs} stands for a diffusion coefficient value for the sumanene in the complex, D_{bound} is a diffusion coefficient value for γCD/HP-γCD in the complex. Finally, K values were calculated using the equation: $K = x_b \cdot [(1- x_b) \cdot (0.00315\ M - x_b \cdot 0.00315\ M)]^{-1}$, where 0.00315 M is a concentration of γCD/HP-γCD and sumanene in the sample.

The hydrodynamic radii of the complexes from ^{1}H DOSY NMR experiments were calculated using the Stokes-Einstein equation (unmodified), given by the equation[14b]:

$$r_{H,solv} = \frac{k_B \mathrm{T}}{6\pi\eta D}$$

where D is the measured diffusion coefficient of the complex (assumed as the hydrodynamic radii for γCD/HP-γCD in the complex taking into account host-guest nature of the studied complexation with sumanene), k_B is the Boltzmann constant ($1.3806485\cdot10^{-23}$ kg·s^{-2}K^{-1}), T is the temperature for the ^{1}H DOSY NMR spectrum acquisition (298.15 K), $r_{H,solv}$ is the calculated hydrodynamic radius, η is the viscosity of the solvent (0.0014405 kg·m^{-1}s^{-1}) at temperature T (η was taken as weighted arithmetic mean with weights equal to vol% of H_2O (0.00089 kg·m^{-1}s^{-1}) and DMSO (0.001991 kg·m^{-1}s^{-1}) in the mixture). The estimated $r_{H,solv}$ were ca. 1.79 nm and 1.81 nm sumanene@γCD and for sumanene@HP-γCD, respectively.

S.1.3. Phase solubility studies

Phase solubility studies were performed as follows. At first, a calibration curve was constructed by measuring the UV-vis spectra of sumanene in DMSO:H_2O = 90:10 *vol*/*vol* at concentrations ranging from 0.002 mM to 0.04 mM. Target phase solubility studies were initialized by the precise weighting of 1.00 mg of sumanene (0.00378 mmol) in the Falcon (15 mL) tubes. To these tubes, an aqueous solution containing given concentration of γCD/HP-γCD (corresponding to the molar equiv. γCD/HP-γCD given in bracket) was added: 0.00 mM (0 equiv.), 1.51 mM (2 equiv.), 3.78 mM (5 equiv.), 7.56 mM (10 equiv.), 15.12 mM (20 equiv.), 75.60 mM (100 equiv.). The tubes were tightly closed and placed in a heating thermostat (50°C) with shaking (300 rpm) for 72 hours. The contents of the tubes were cooled to room temperature, and filtered through a syringe Nylon filter (0.22 μM). An aliquot was taken to dilute the sample 10x with DMSO. After shaking the sample, it was subjected to the UV-vis spectra analysis. The concentration of sumanene in each sample was calculated based on the as-constructed calibration curve. The K value for sumanene@HP-γCD, for which linear dependence was found for the $C_{sumanene} = f(C_{HP\text{-}\gamma CD})$ plot, was calculated using the following equation:

K = (slope/$C_{0\text{-sumanene}}$) × (1-slope)$^{-1}$, where $C_{0\text{-sumanene}}$ is the estimated solubility of sumanene in water without the presence of HP-γCD in the phase solubility test experiment (estimated from the calibration curve as 0.00000005 M).[15]

S.1.4. Density functional theory (DFT) computations

Density functional theory (DFT) computations for sumanene@γCD complexes were performed with Gaussian software[16] with B3LYP functional[17] and 6-31g(d,p) basis set (in a gas phase)[18]. The initial structures of sumanene and γCD were adopted from their crystal structures, which were further modified with Avogadro[19] software, and then subjected to calculation. Structures of the complexes were initially constructed in GaussView[20] software using DFT-optimized sumanene and γCD structures, and then subjected to calculation. At first, structure optimization was performed, and then, vibrational frequencies were calculated to provide thermal free energy correction to the electronic energy. Interaction energies (Gibbs free energies of association, ΔG) values were provided in kcal/mol. It should be noted that it was confirmed that all optimized complexes were stable geometric structures since no imaginary frequencies were detected. In order to speed up these highly demanding computations (in terms of required computational power) the computations were performed with the support of the Interdisciplinary Centre for Mathematical and Computational Modelling University of Warsaw (ICM UW) under computational allocation no G98-2059.

S.1.5. Synthesis and analysis of solid complexes

Grinding-induced synthesis of complexes was performed with a RETSCH MM 400 ball mill. The synthesis was performed as follows. Sumanene (11.42 mg, 0.043 mmol) and γCD/HP-γCD (56.00 mg/68.00 mg, 0.043 mmol) were placed in a 5 mL grinding jar and three grinding balls ϕ 5 mm were placed in the jar. 10 μL of distilled water was added, and the content of the jar was subjected to intensive grinding for 2 hours at 30 Hz. The resultant yellowish mixture was suspended in distilled water (in total *ca*. 10 mL of water was used to transfer the solid). Then, it was filtrated off on a Nylon membrane (0.45 μm), washed with cold distilled water (*ca*. 50 mL), a slight amount of cold THF (ca. 0.5 mL), and cold acetone (*ca*. 30 mL). Finally, the solids were dried on air for several days. In both cases, bright yellow solids were obtained. The isolated yield for sumanene@γCD and sumanene@HP-γCD was 87% (58.39 mg) and 60% (47.58 mg), respectively. The lower yield for sumanene@HP-γCD could be reasoned with the higher water solubility of HP-γCD than γCD resulting in greater weight losses during the washing stage.

^{1}H qNMR analyses with mechanochemically-prepared sumanene@γCD inclusion complex were performed with *N,N*-dimethylformamide (anhydrous, Merck, purity ≥99.8%) as the reference material.[21–23] The formula applied for the calculation of the sample purity (P_{sample}) and collected data (provided in bracket) were as follows:

$$P_{sample} = P_{ref} \cdot (I_{sample}/I_{ref}) \cdot (N_{ref}/N_{sample}) \cdot (M_{sample}/M_{ref}) \cdot (m_{ref}/m_{sample})$$

, where P_{sample} is the estimated purity of the sample (**≥97%**), P_{sample} is the purity of reference sample (provided above), I_{sample} is the integral value for sample signals (41.03; for signals observed at 4.50–7.18 ppm), I_{ref} is the integral value for reference signal (1.03; for signal observed at 7.95 ppm, singlet), N_{ref} is the number of reference nuclei (1 for selected signal), N_{sample} is the number of sample nuclei (41 for selected signals), M_{sample} is the molecular mass of the sample (1560.7426 g/mol), M_{ref} is the molecular mass of the reference (73.095 g/mol), m_{ref} is the mass of the reference (0.0001106 g; this value was calculated taking into account the exact mass of the precisely weighted 0.00479 M stock solution of the reference and the dilution factor during sample preparation), m_{sample} is the precisely weighted mass of the sample (0.00237 g).

ESI-MS (TOF) measurements with sumanene@γCD inclusion complex were performed with a Q-Exactive ThermoScientific spectrometer. The simulated isotope pattern was generated with Isotope Pattern Calculator v4.0, www: https://yanjunhua.tripod.com/pattern.htm.

Fourier-transform infrared (FT-IR) spectra were recorded in an attenuated total reflection (ATR) mode with a Thermo Scientific Nicolet iS5 spectrometer with a spectral resolution of 2 cm^{-1}.

S.1.6. *In sillico* modeling of pharmacokinetic properties (ADME-Tox)

***In sillico* modeling of pharmacokinetic properties** (**ADME-Tox**) for sumanene, sumanene@γCD complex and sumanene@HP-γCD complex (representative tetra-substitution of γCD with HP units was subjected to modeling) was performed with pkCSM.[24–26]

S.1.7. Biological studies

Biological studies were performed as follows. Breast cancer cells (MDA-MB 231, ATCC Europe Collection) or human mammary fibroblasts (HMF, ScienCell) were seeded on 96 well plates with a density of 10^4 cells/well. Then, breast cells were incubated with tested compounds within 24 hours (solutions of tested compounds were performed by dissolving solid compounds in a minimal amount of DMSO and sterile distilled water was added to such DMSO solution in the maximum possible volume that did not cause the precipitation in the sample). Cells incubated with non-compounds were used as a control. After 24 hours of incubation, alamarBlue® (AB, Serotec Ltd., Oxford, UK) assay was performed. For this purpose, 10% of AB solution, prepared in a cell culture medium, was added to each well and incubated for 1 hour (5% CO_2, 37°C). After this, the fluorescence intensity was measured using a plate reader (Cytation™3, BioTek) at the excitation and emission wavelength of 552 nm and 583 nm, respectively. The results were presented as a % of control.

S2. NMR analyzes on the supramolecular interactions

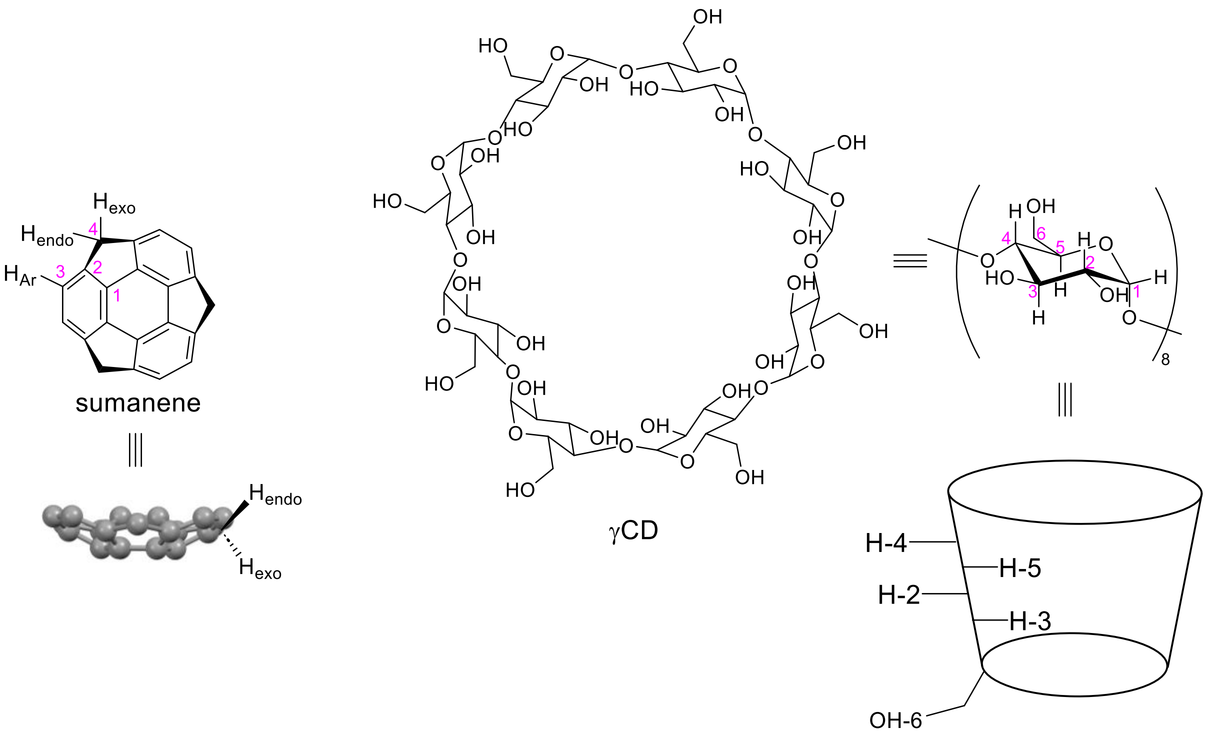

Scheme. S1. Atom numbering (labels) for sumanene[27] and γCD[28] together with the graphical representation of their spatial structures.

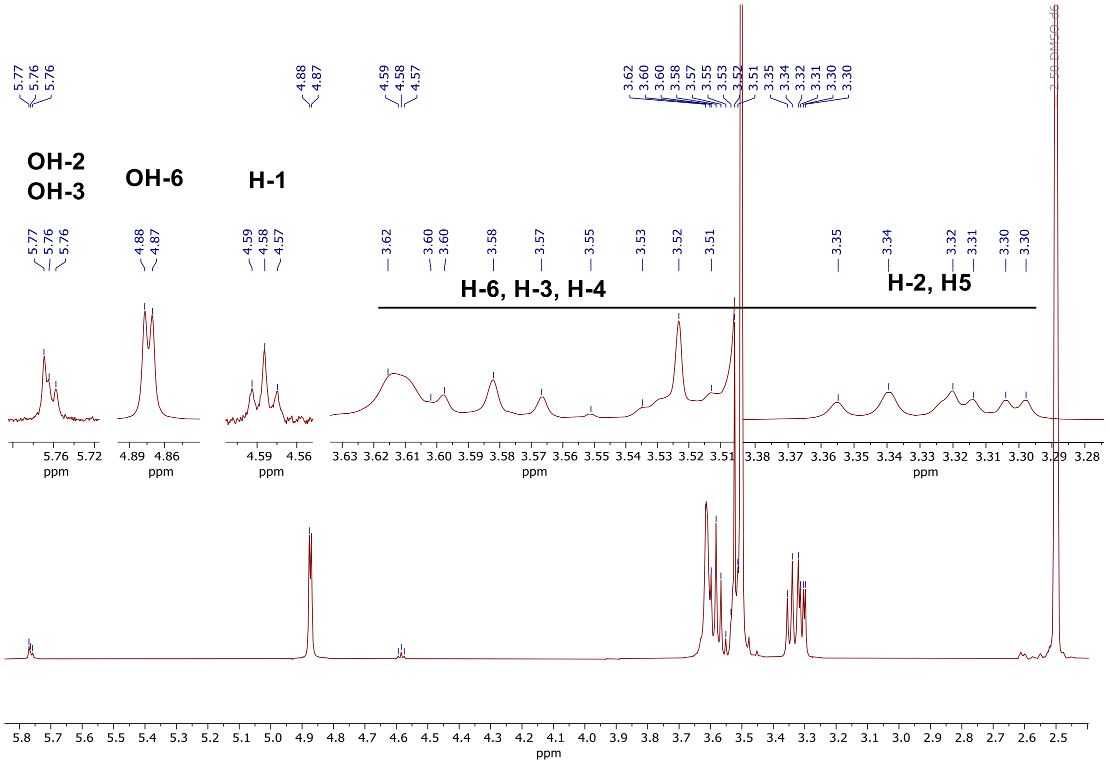

Fig. S1. ^{1}H NMR spectrum (600 MHz, 5 *vol*% D_2O in DMSO-d_6; $C_{\gamma CD}$ = 3.15 mM) of γCD with marked plausible signals assignments (these were based on the literature references[28,29]).

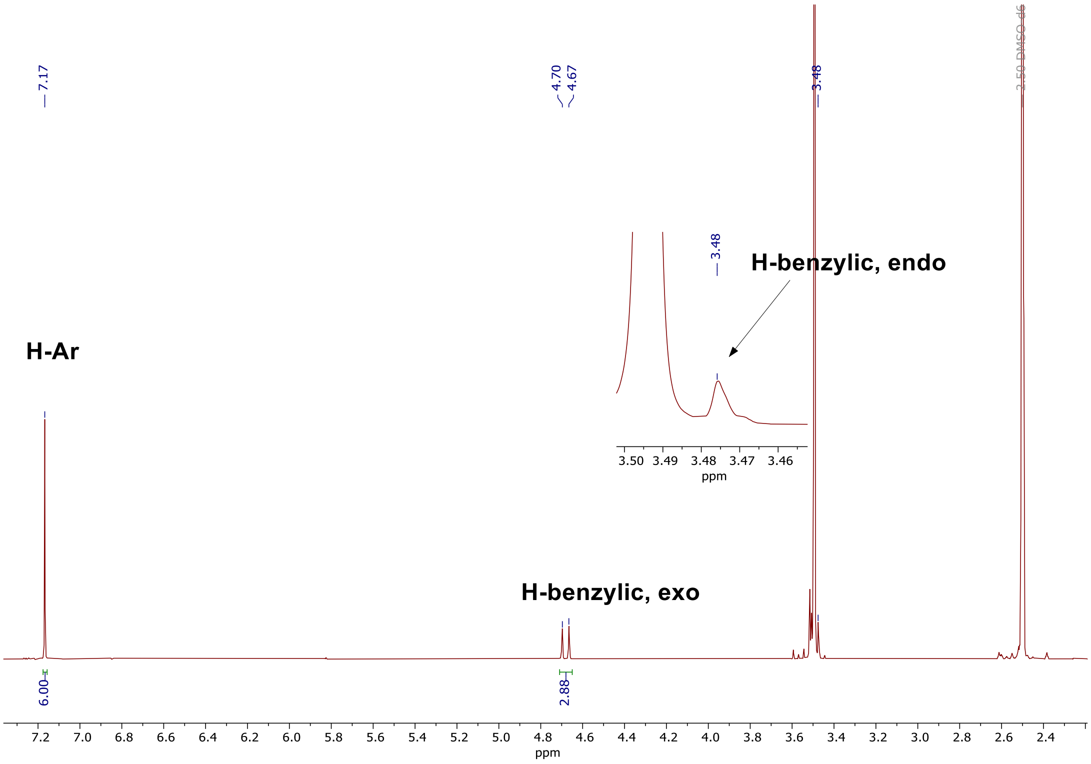

Fig. S2. ^{1}H NMR spectrum (600 MHz, 5 *vol*% D_2O in DMSO-d_6; $C_{sumanene}$ = 3.15 mM) of sumanene with marked signals assignments.

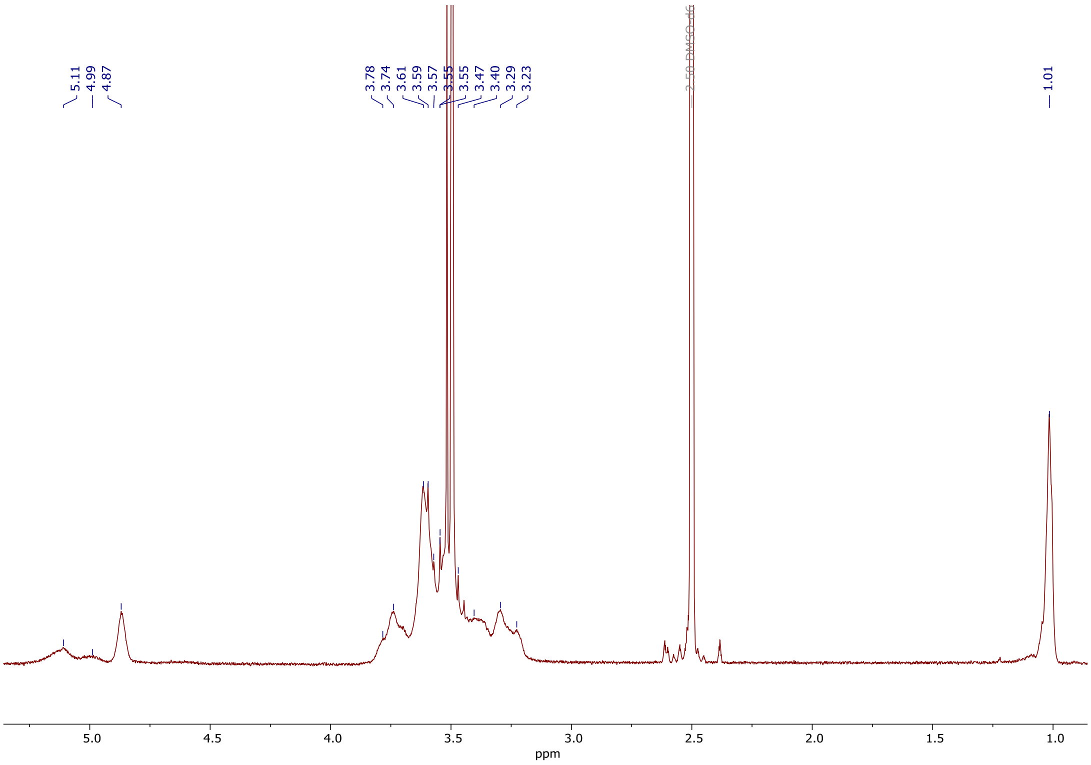

Fig. S3. ^{1}H NMR spectrum (600 MHz, 5 *vol*% D_2O in DMSO-d_6; $C_{\gamma CD}$ = 3.15 mM) of HP-γCD.

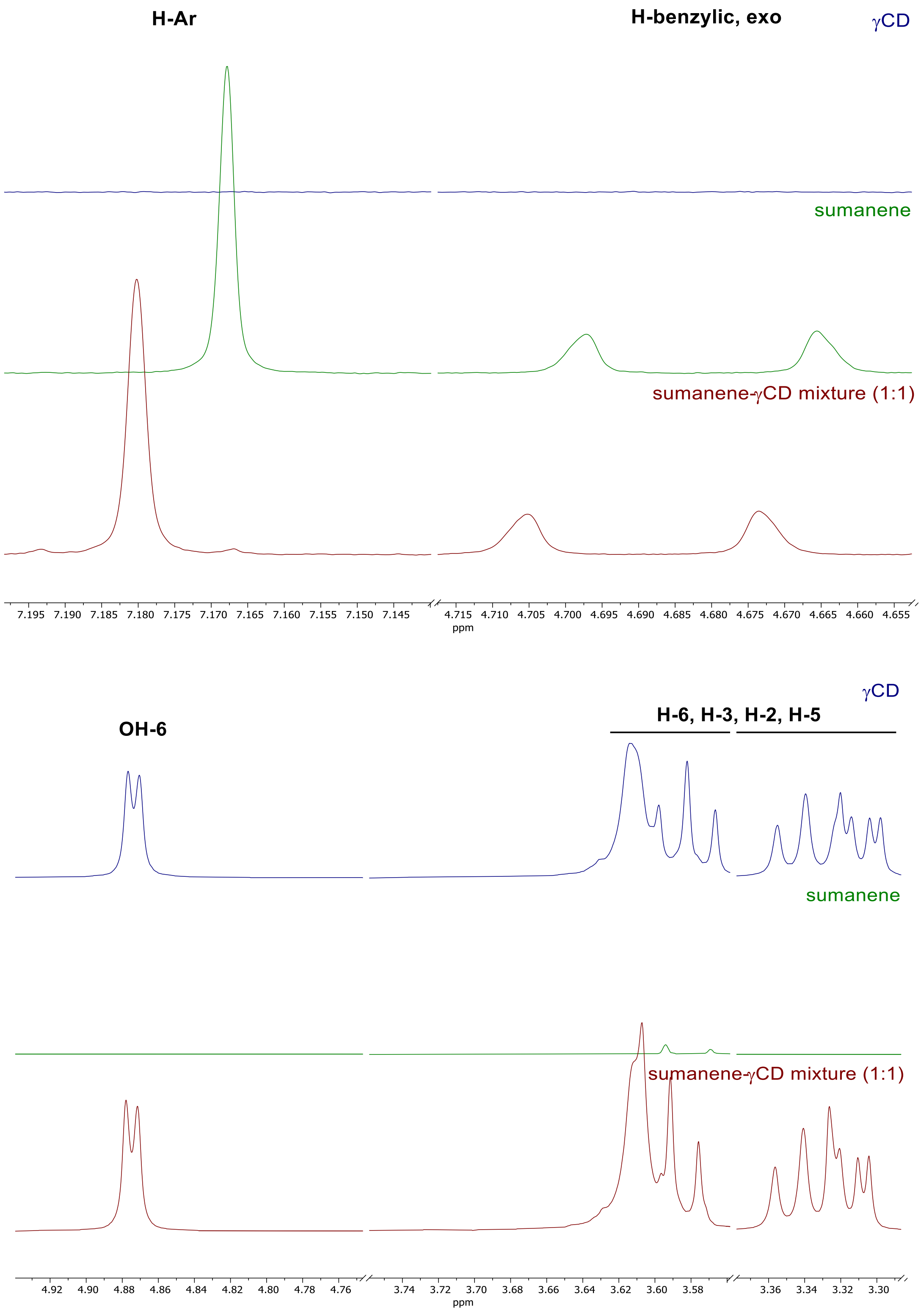

Fig. S4. Selected key-insets of ^{1}H NMR spectra (600 MHz, 5 *vol*% D_2O in DMSO-d_6; $C_{sumanene}$ = 3.15 mM; $C_{\gamma CD}$ = 3.15 mM) of sumanene, γCD and their 1:1 mixture: (top) for sumanene, (bottom) for γCD.

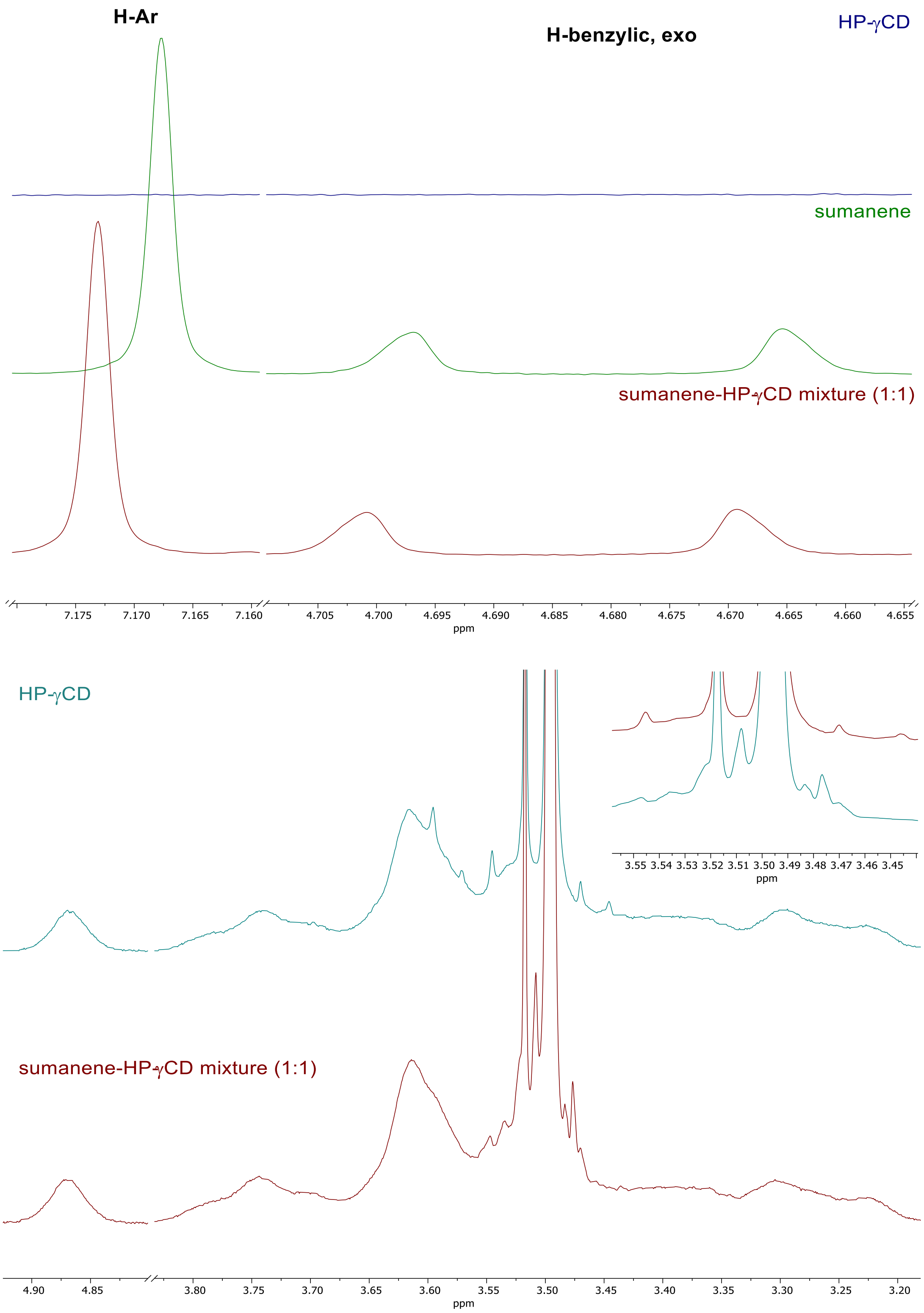

Fig. S5. Selected key insets of ^{1}H NMR spectra (600 MHz, 5 *vol*% D_2O in DMSO-d_6; $C_{sumanene}$ = 3.15 mM; $C_{HP-\gamma CD}$ = 3.15 mM) of sumanene, HP-γCD and their 1:1 mixture: (top) for sumanene, (bottom) for HP-γCD (in the bottom stacked spectra sumanene spectrum was subtracted for the clarity of the image; inset presenting the changes in the chemical shift is also presented).

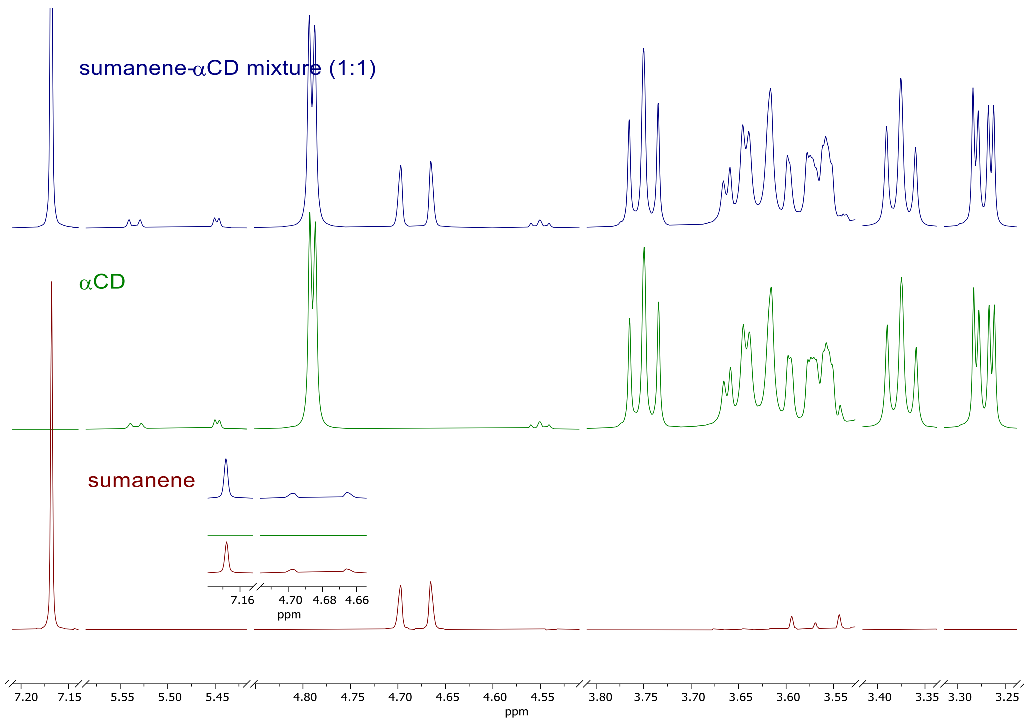

Fig. S6. Selected key-insets of ^{1}H NMR spectra (600 MHz, 5 *vol*% D_2O in DMSO-d_6; C_{sumanene} = 3.15 mM; $C_{\alpha\text{CD}}$ = 3.15 mM) of sumanene, αCD and their 1:1 mixture: (top) for sumanene, (bottom) for αCD (the inset for H-sumanene is also presented).

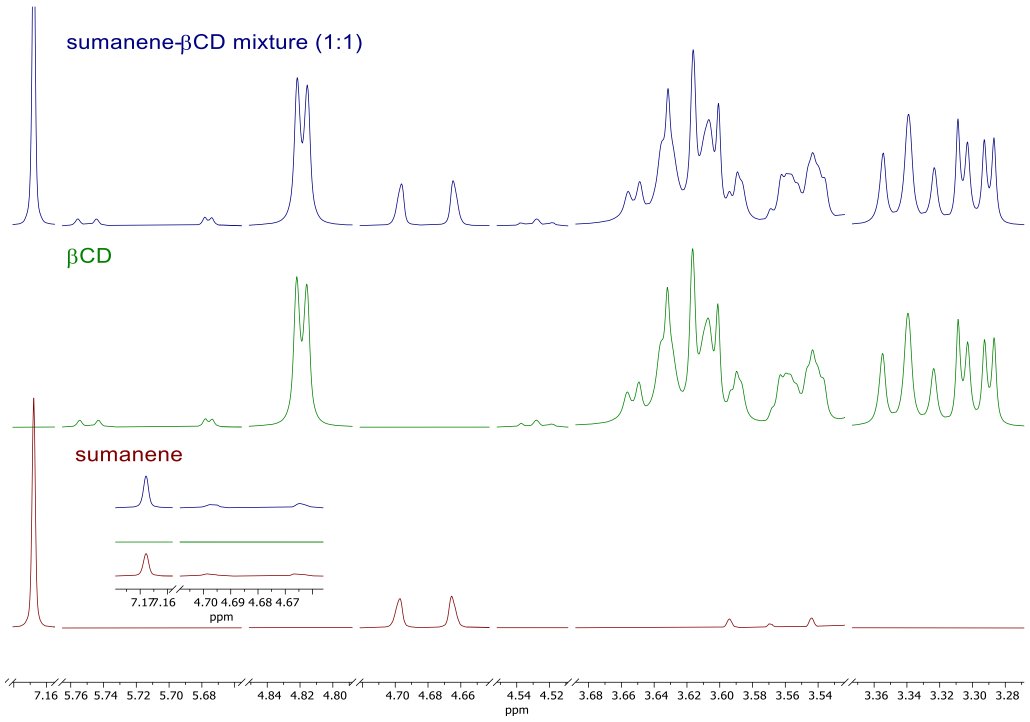

Fig. S7. Selected key-insets of ^{1}H NMR spectra (600 MHz, 5 *vol*% D_2O in DMSO-d_6; C_{sumanene} = 3.15 mM; $C_{\beta\text{CD}}$ = 3.15 mM) of sumanene, βCD and their 1:1 mixture: (top) for sumanene, (bottom) for βCD (the inset for H-sumanene is also presented).

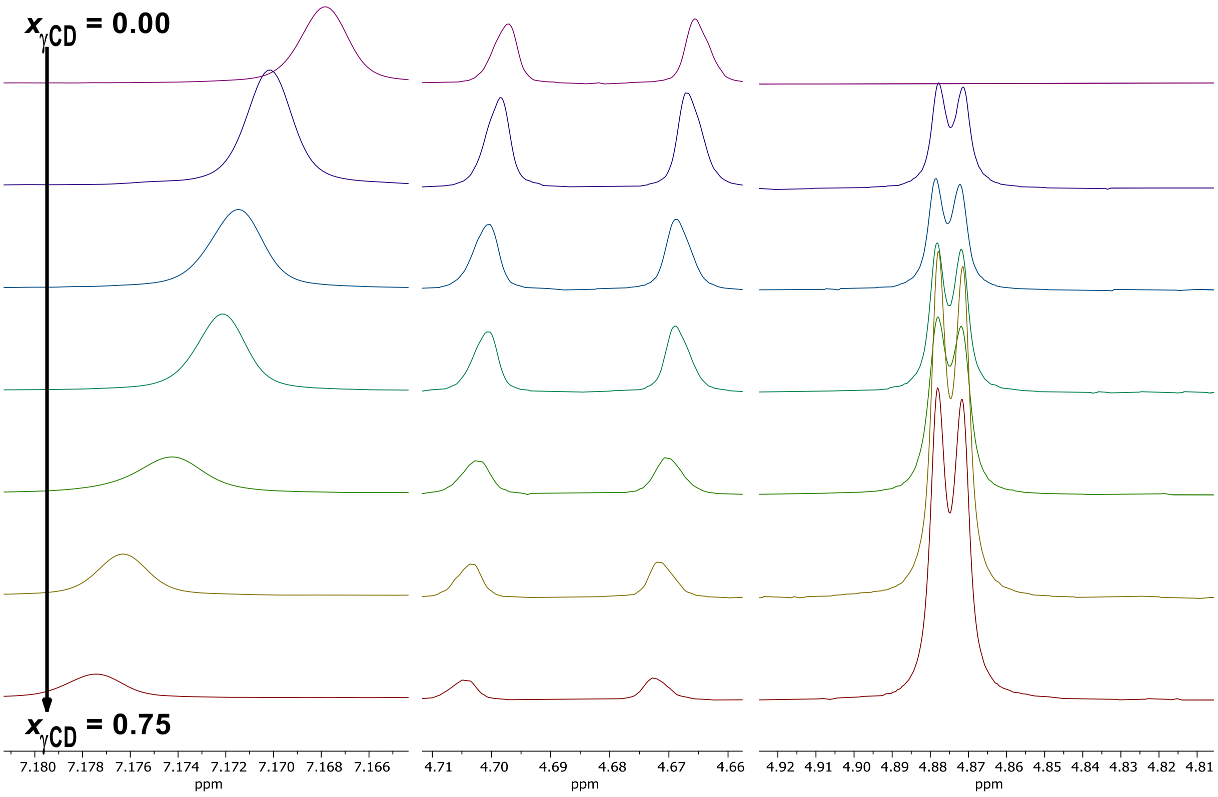

Fig. S8. Evolution of selected key-insets of ^1H NMR spectra (600 MHz, 5 *vol*% D_2O in DMSO-d_6) regarding Job's plot analyzes with sumanene and γCD.

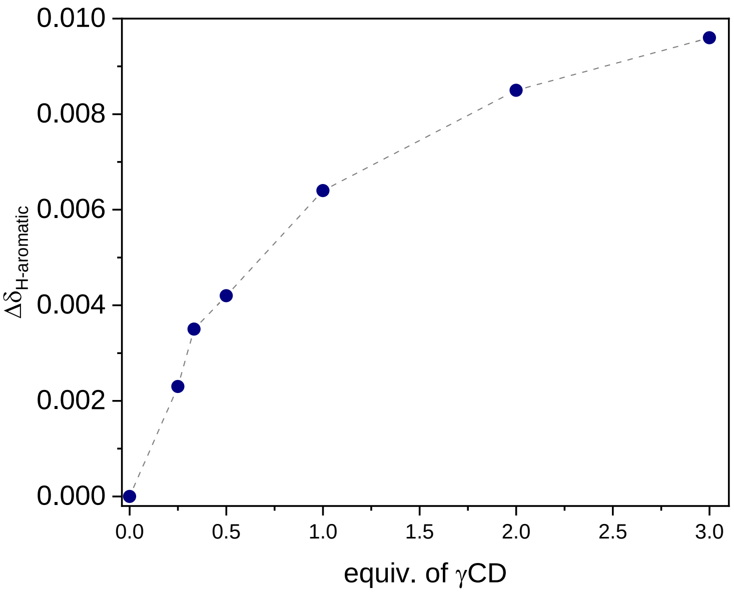

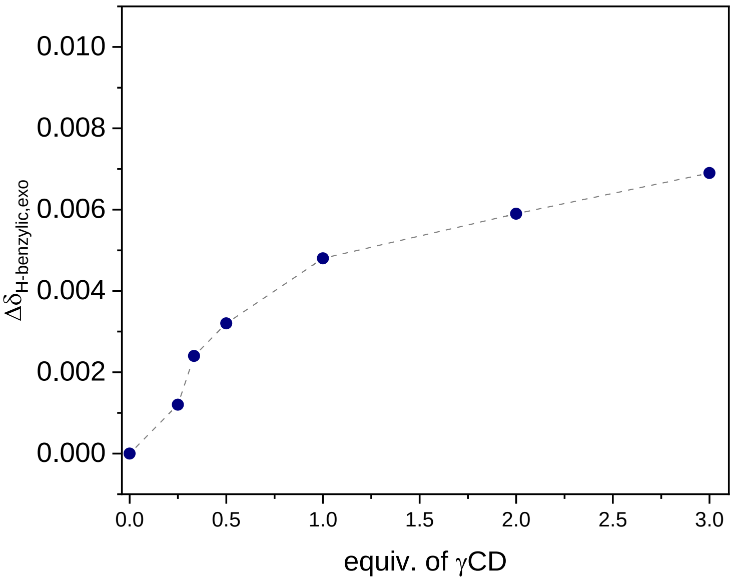

Fig. S9. Changes in the δ_H for H_{Ar} (top) and $H_{benzylic,exo}$ (bottom) of sumanene regarding ^1H NMR spectra (600 MHz, 5 *vol*% D_2O in DMSO-d_6) Job's plot analyzes with sumanene and γCD.

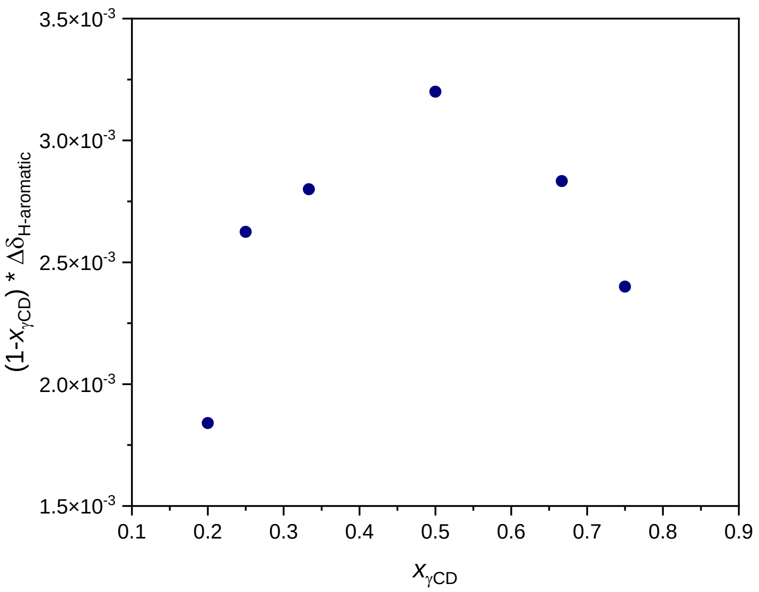

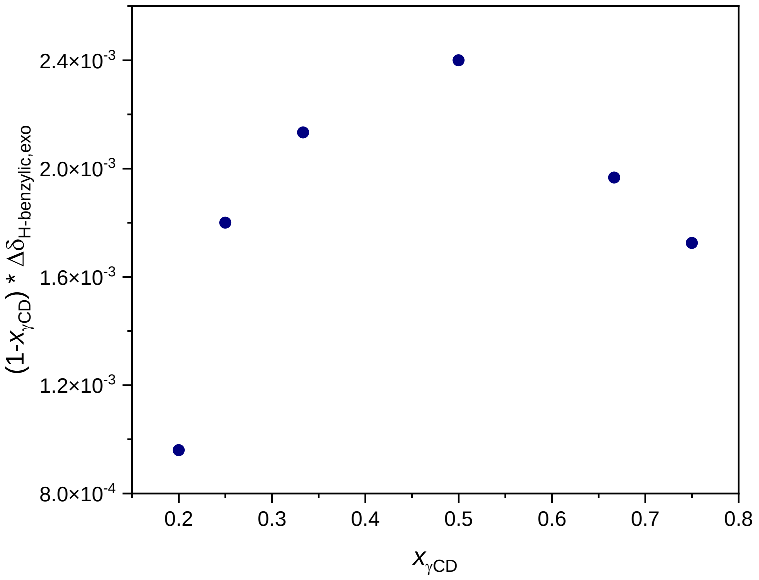

Fig. S10. Job's plots regarding ^{1}H NMR spectra (600 MHz, 5 *vol*% D_2O in DMSO-d_6) analyzes with sumanene and γCD: for δ_H of H_{Ar} (top) and $H_{benzylic,exo}$ (bottom) of sumanene.

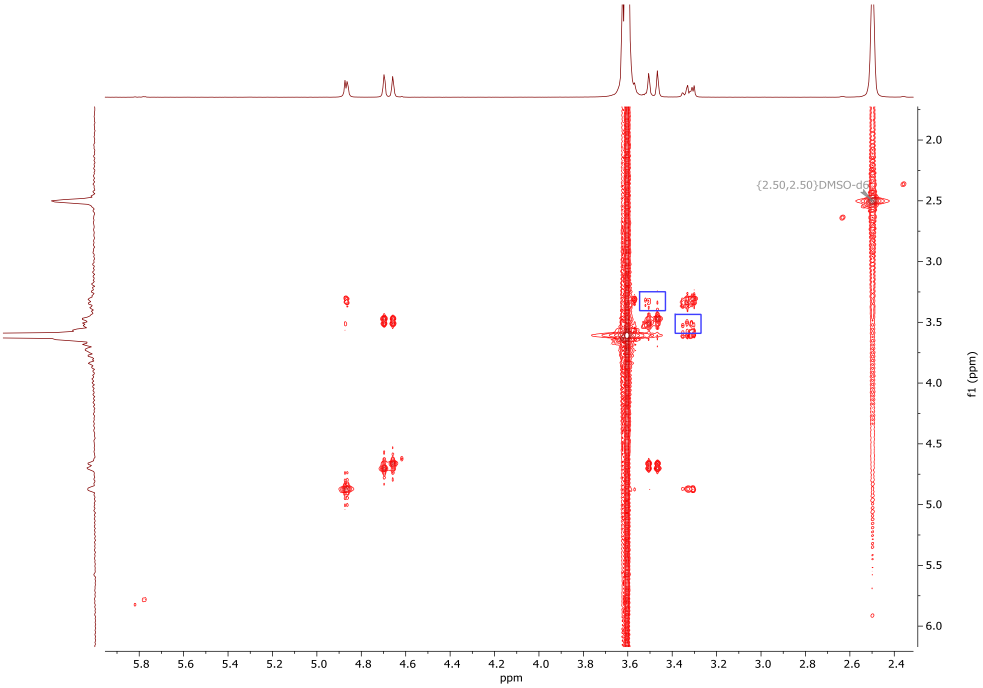

Fig. S11. The key-inset of ^{1}H-^{1}H ROESY NMR spectrum (500 MHz, 8 *vol*% D_2O in DMSO-d_6; $C_{sumenene}$ = 3.15 mM; $C_{\gamma CD}$ = 3.15 mM) of 1:1 mixture of sumanene and γCD presenting the crucial cross-correlation (marked blue).

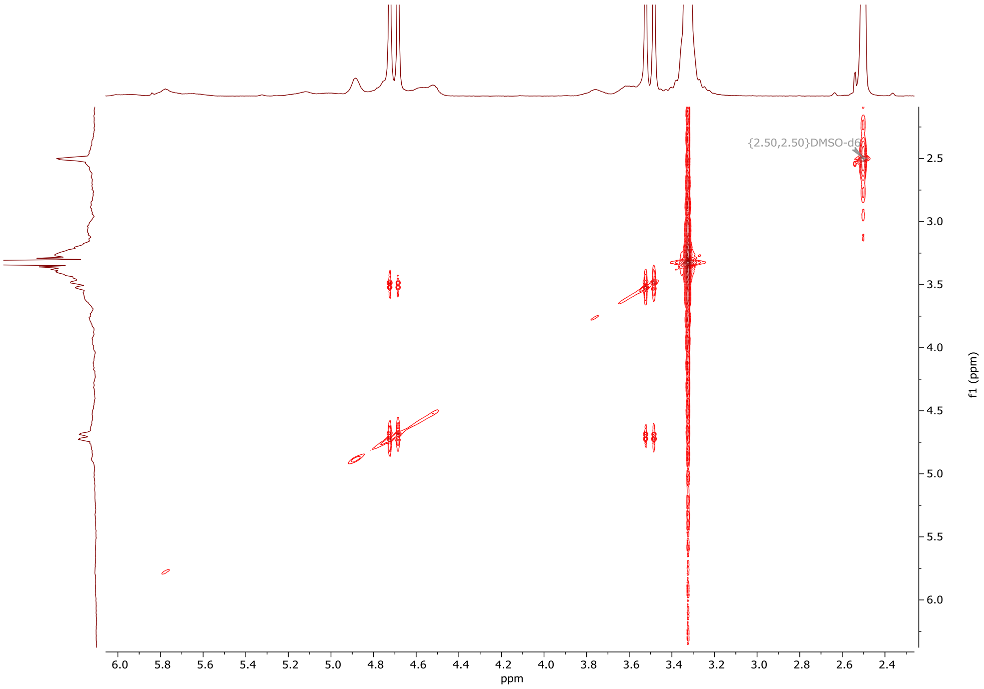

Fig. S12. The key-inset of ^{1}H-^{1}H ROESY NMR spectrum (500 MHz, DMSO-d_6; $C_{sumanene}$ = 3.15 mM; $C_{\gamma CD}$ = 3.15 mM) of 1:1 mixture of sumanene and γCD presenting the lack of crucial cross-correlation observed in the spectrum acquired in 5 *vol*% D_2O in DMSO-d_6.

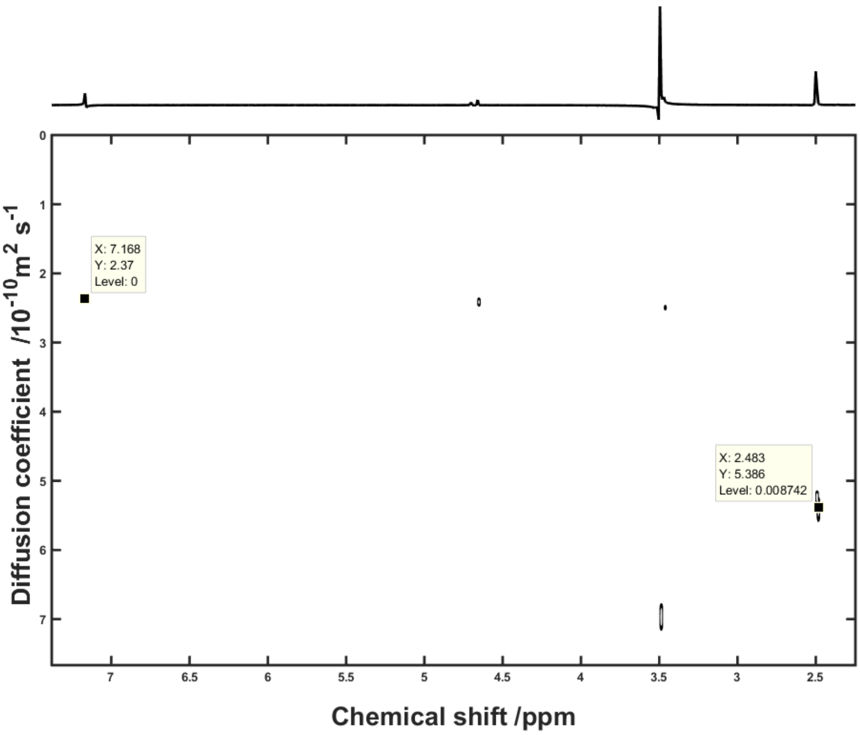

Fig. S13. ^{1}H DOSY NMR spectrum (500 MHz, 5 *vol*% D_2O in DMSO-d_6; C_{sumanene} = 3.15 mM; 297.15 K) of sumanene.

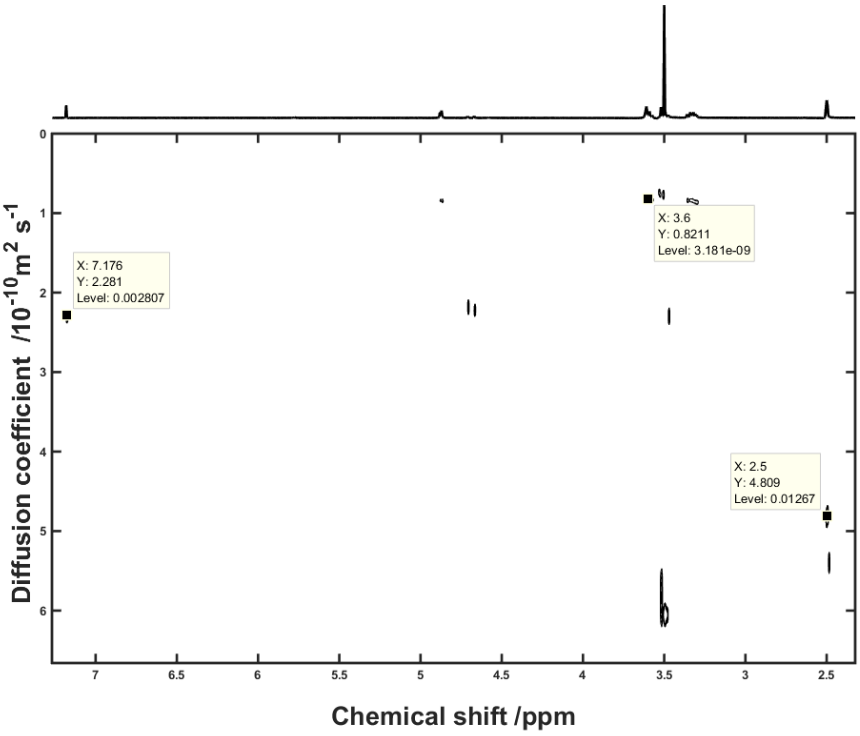

Fig. S14. ^{1}H DOSY NMR spectrum (500 MHz, DMSO-d_6; C_{sumanene} = 3.15 mM; $C_{\gamma\text{CD}}$ = 3.15 mM; 297.15 K) of 1:1 mixture of sumanene and γCD.

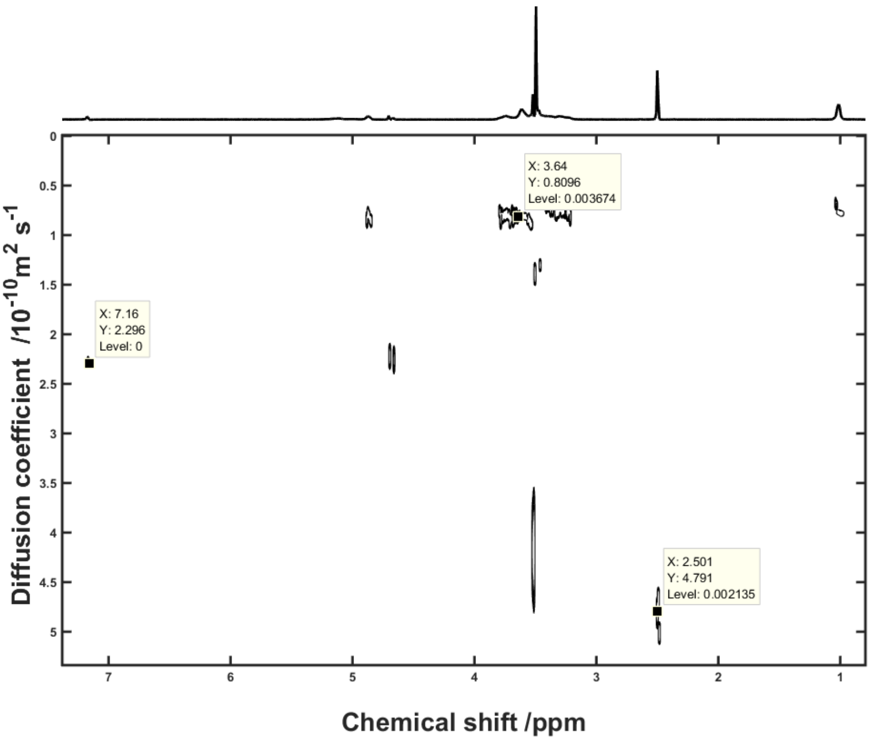

Fig. S15. ^{1}H DOSY NMR spectrum (500 MHz, DMSO-d_6; $C_{sumanene}$ = 3.15 mM; $C_{HP\text{-}\gamma CD}$ = 3.15 mM; 297.15 K) of 1:1 mixture of sumanene and HP-γCD.

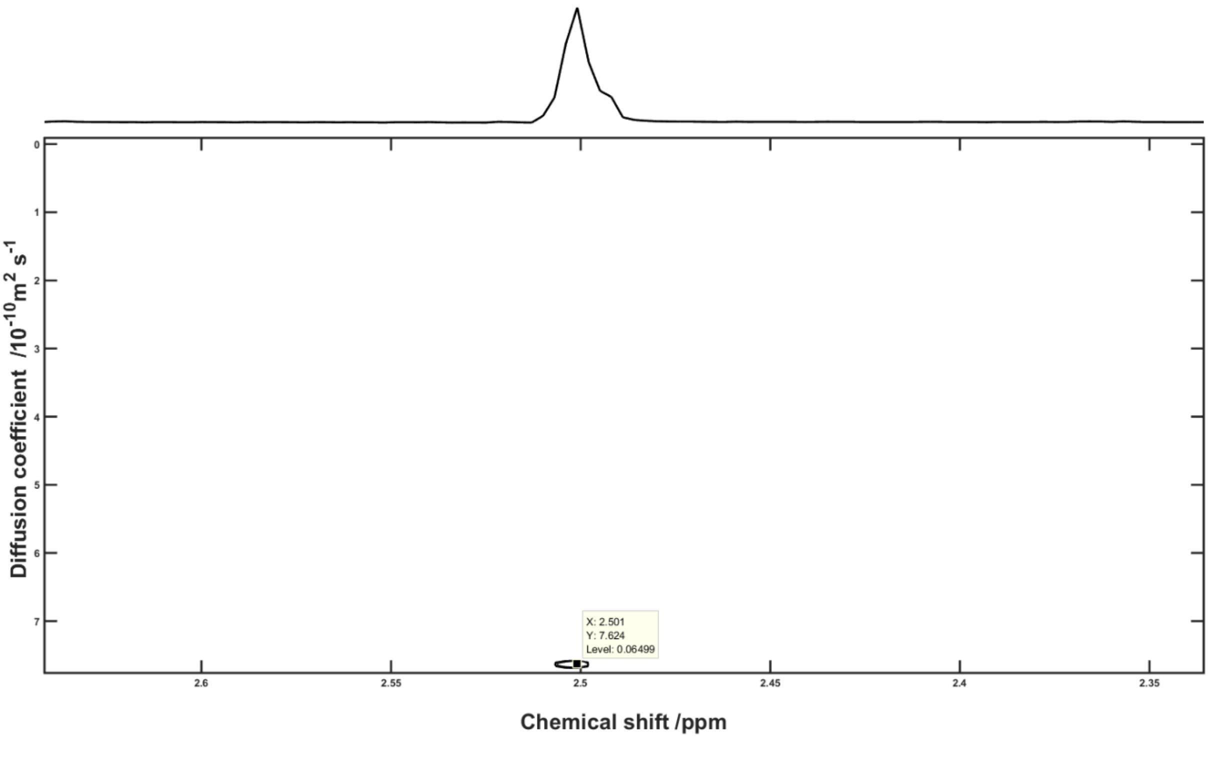

Fig. S16. ^{1}H DOSY NMR spectrum (500 MHz, 318.15 K) of 5 *vol*% D_2O/DMSO-d_6.

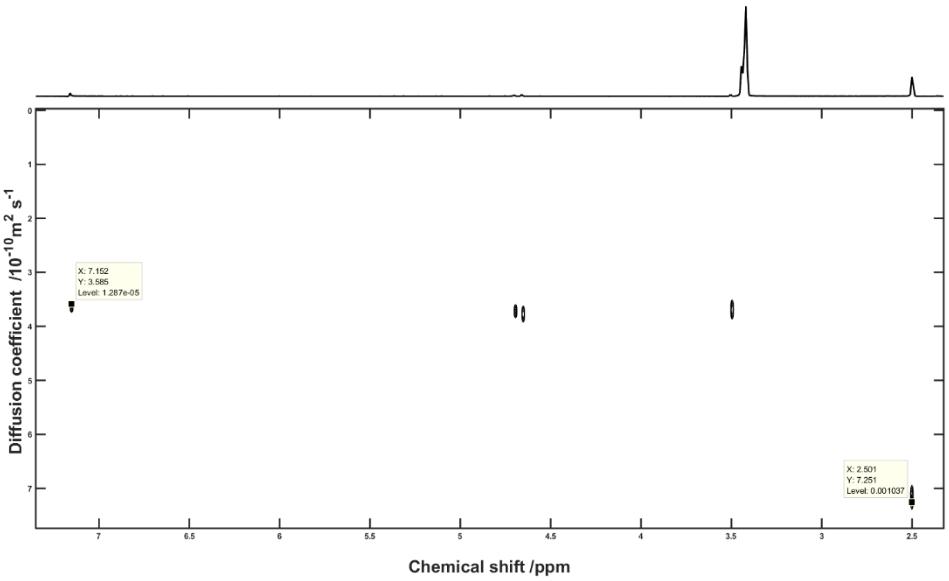

Fig. S17. ^{1}H DOSY NMR spectrum (500 MHz, 5 *vol*% D_2O in DMSO-d_6; $C_{sumanene}$ = 3.15 mM; 318.15 K) of sumanene.

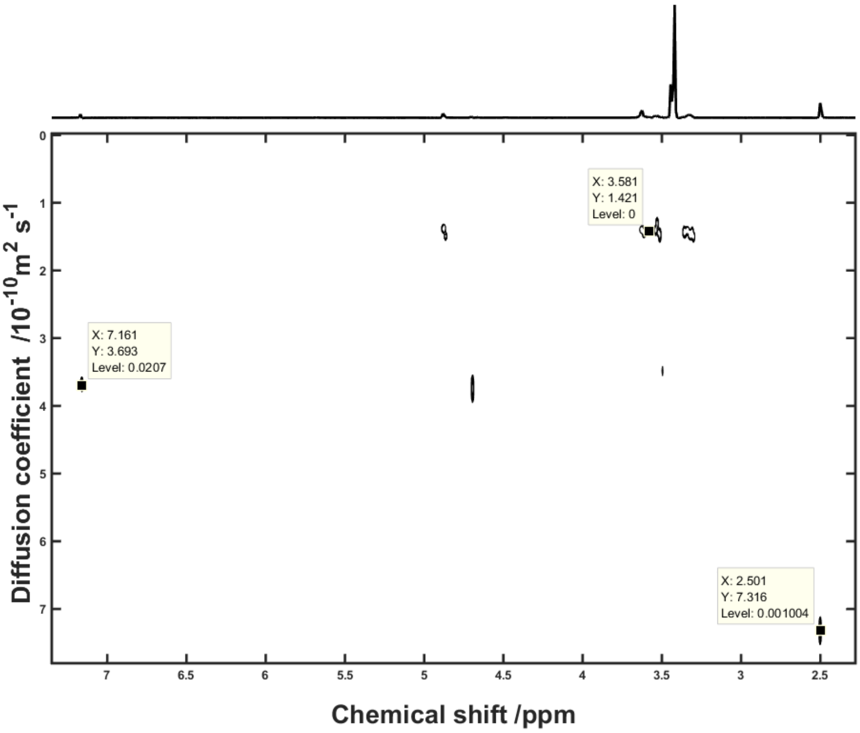

Fig. S18. ^{1}H DOSY NMR spectrum (500 MHz, DMSO-d_6; $C_{sumanene}$ = 3.15 mM; $C_{\gamma CD}$ = 3.15 mM; 318.15 K) of 1:1 mixture of sumanene and γCD.

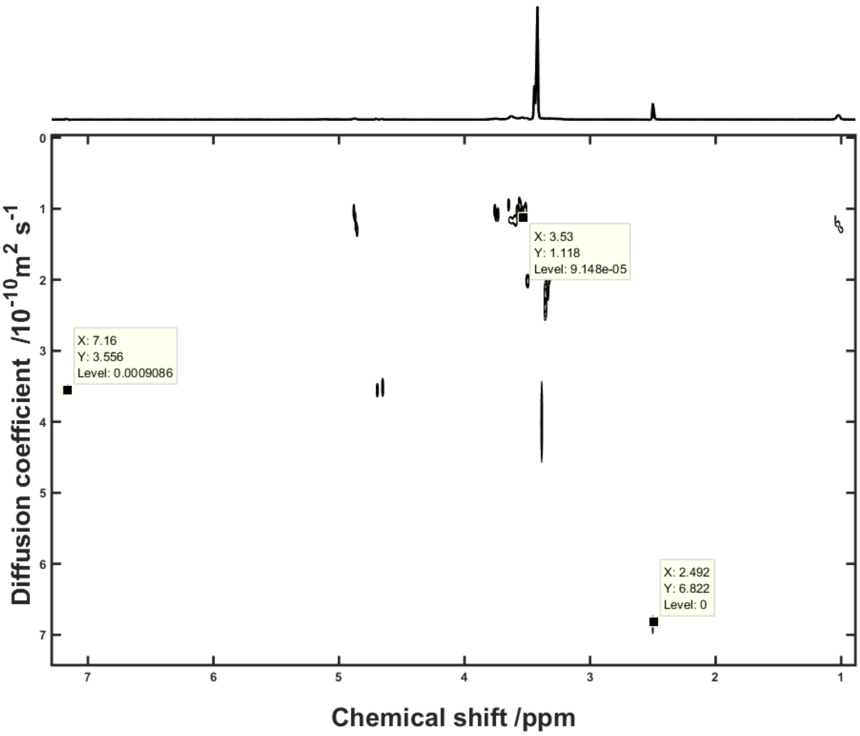

Fig. S19. ^{1}H DOSY NMR spectrum (500 MHz, DMSO-d_6; $C_{sumanene}$ = 3.15 mM; $C_{HP\text{-}\gamma CD}$ = 3.15 mM; 318.15 K) of 1:1 mixture of sumanene and HP-γCD.

Table S1. Diffusion coefficient parameters and calculated apparent constant (K) values from ^{1}H DOSY NMR experiments. Diffusion coefficient values (D) were provided in the table in 10^{-10} m^2/s. The method of calculation and description parameters are provided in Section S1.2.

Complex	Cpd.	D_{free}	D_{obs}	D_{bound}	x_b	K (M^{-1})	ΔG (kJ × mol^{-1})
at 297.15 K							
sumanene@γCD	γCD	N/A	N/A	0.848	0.127	**53.10**	**−9.81**
	sumanene	2.172	2.353	N/A			
sumanene@HP-γCD	HP-γCD	N/A	N/A	0.838	0.145	**62.70**	**−10.23**
	sumanene	2.172	2.377	N/A			
at 318.15 K							
sumanene@γCD	γCD	N/A	N/A	1.481	0.035	**11.76**	**−6.52**
	sumanene	3.769	3.845	N/A			
sumanene@HP-γCD	HP-γCD	N/A	N/A	1.249	0.081	**30.51**	**−9.04**
	sumanene	3.769	3.974	N/A			

S3. Fluorescence spectroscopy analyzes on the supramolecular interactions

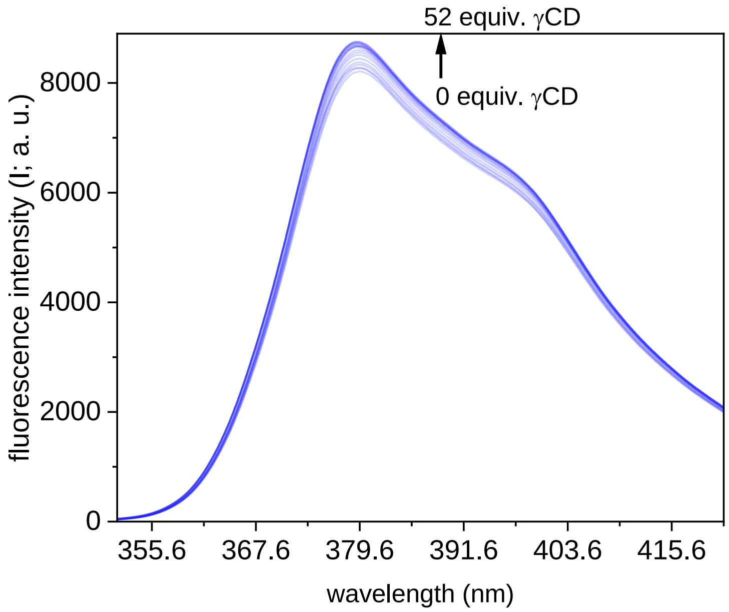

Fig. S20. Evolution of the fluorescence spectra titration (DMSO:H_2O = 2:1 *vol/vol;* $C_{sumenene}$ = 0.02 mM; λ_{ex} = 280 nm) on the supramolecular interactions between sumanene and γCD.

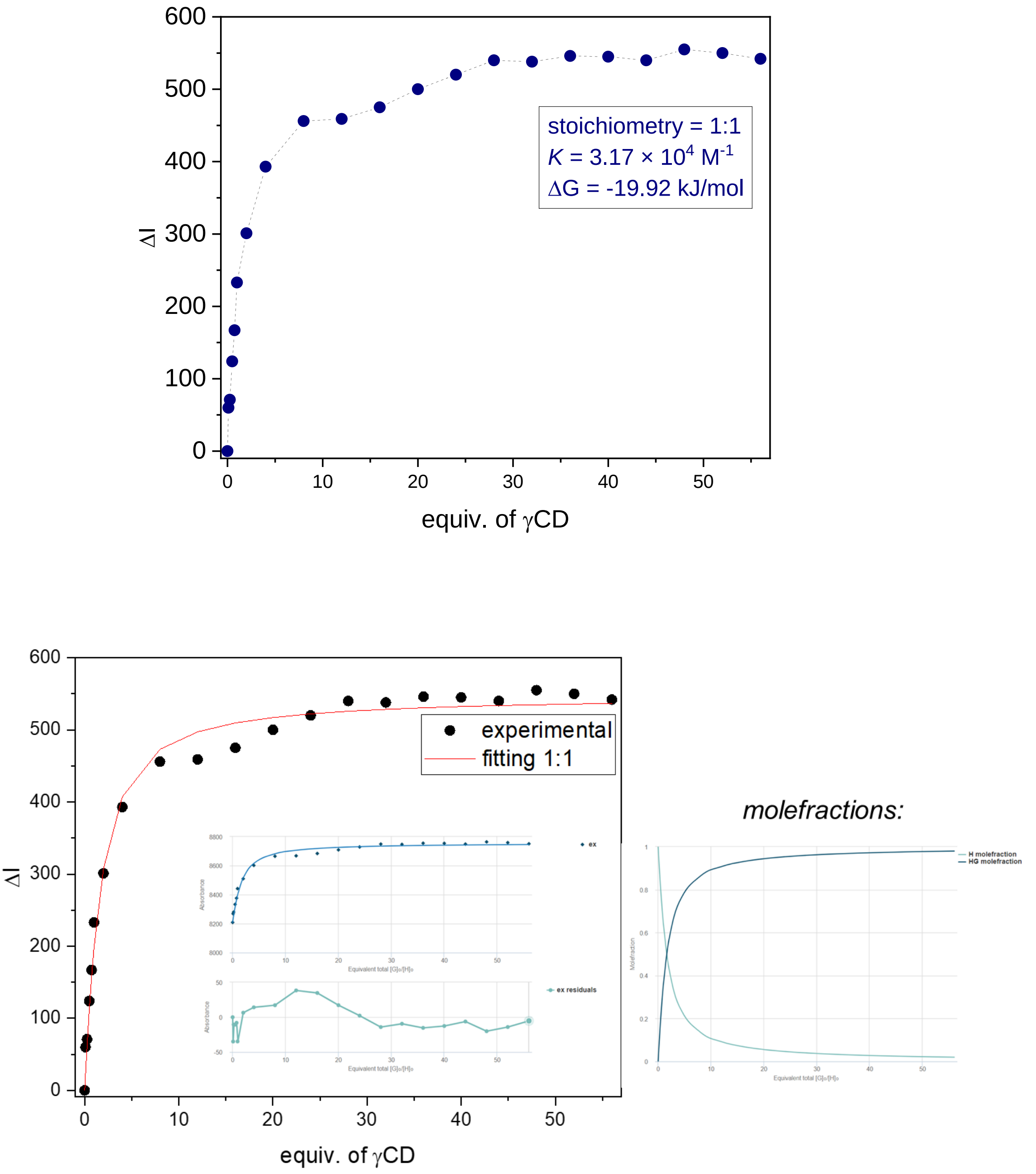

Fig. S21. Fluorescence titration curve (DMSO:H_2O = 2:1 *vol/vol;* $C_{sumenene}$ = 0.02 mM; λ_{em} = 379 nm) for supramolecular interactions between sumanene and γCD, binding parameters are also presented in the frame; , binding parameters are also presented in the frame; (bottom) Global fitting to the 1:1 binding model with Bindfit, K = 4.4±0.8 × 10^4 M^{-1}, the raw images from the Bindfit fitting are also presented.

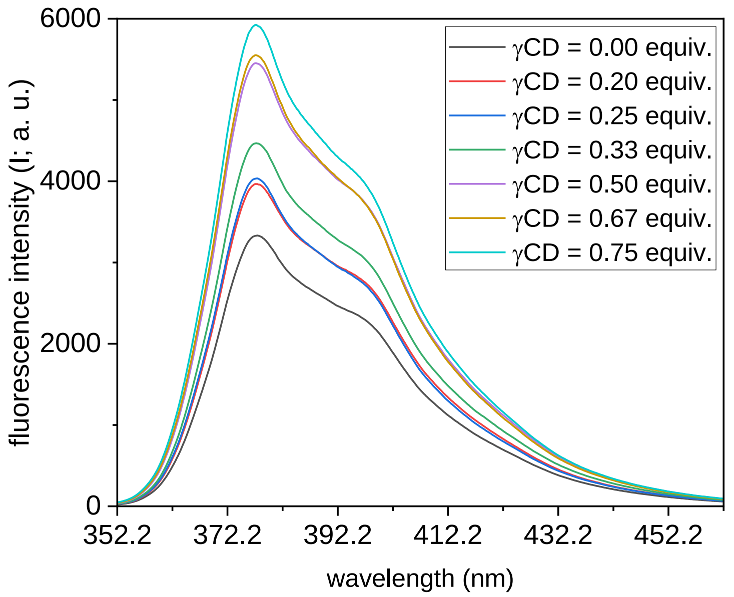

Fig. S22. Fluorescence spectra for Job's plot analyzes on supramolecular interactions between sumanene and γCD (DMSO:H_2O = 2:1 *vol/vol;* λ_{ex} = 280 nm; λ_{em} = 379 nm).

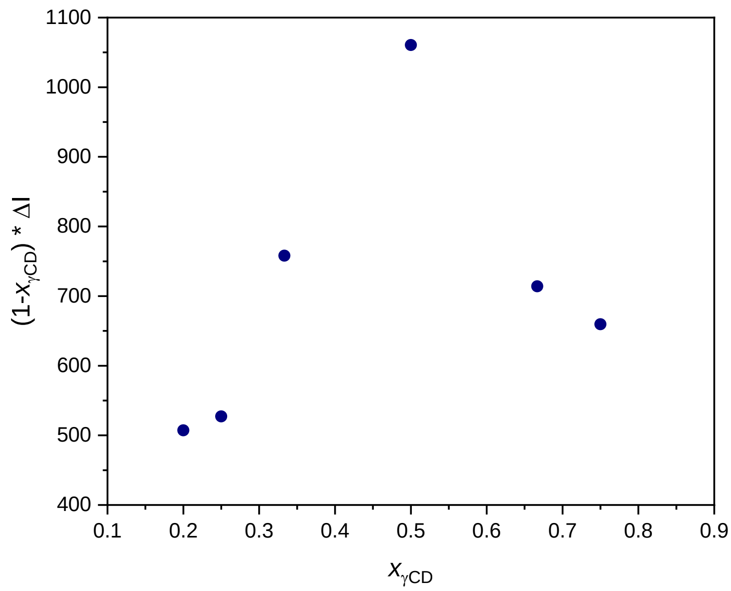

Fig. S23. Job's plot for supramolecular interactions between sumanene and γCD probed with fluorescence spectroscopy (DMSO:H_2O = 2:1 *vol/vol;* λ_{ex} = 280 nm; λ_{em} = 379 nm).

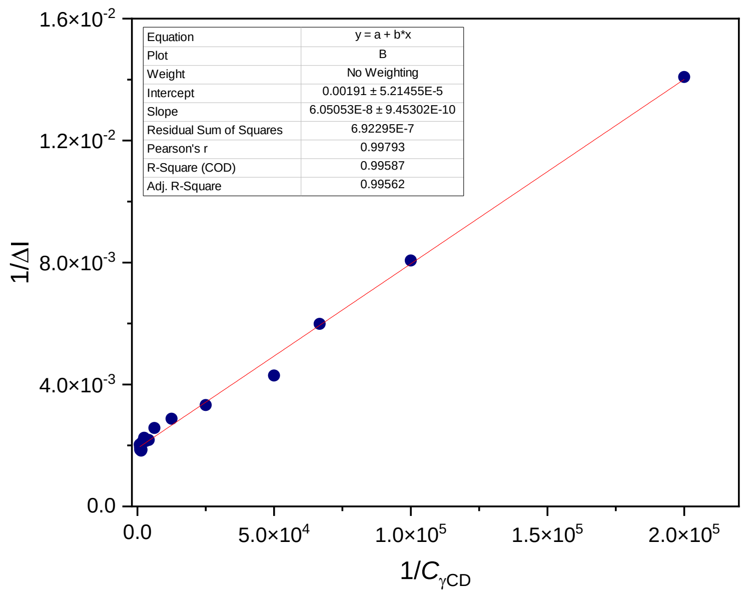

Fig. S24. Benesi-Hildebrand plot for supramolecular interactions between sumanene and γCD probed with fluorescence spectroscopy (DMSO:H_2O = 2:1 *vol/vol;* $C_{sumenene}$ = 0.02 mM; λ_{em} = 379 nm). Linear regression parameters are also presented.

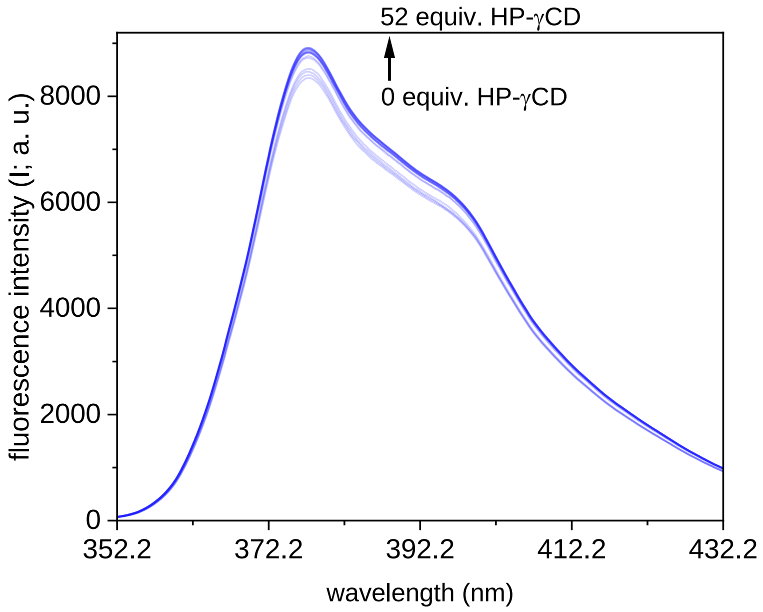

Fig. S25. Evolution of the fluorescence spectra titration (DMSO:H_2O = 2:1 *vol/vol;* $C_{sumenene}$ = 0.02 mM; λ_{ex} = 280 nm) on the supramolecular interactions between sumanene and HP-γCD.

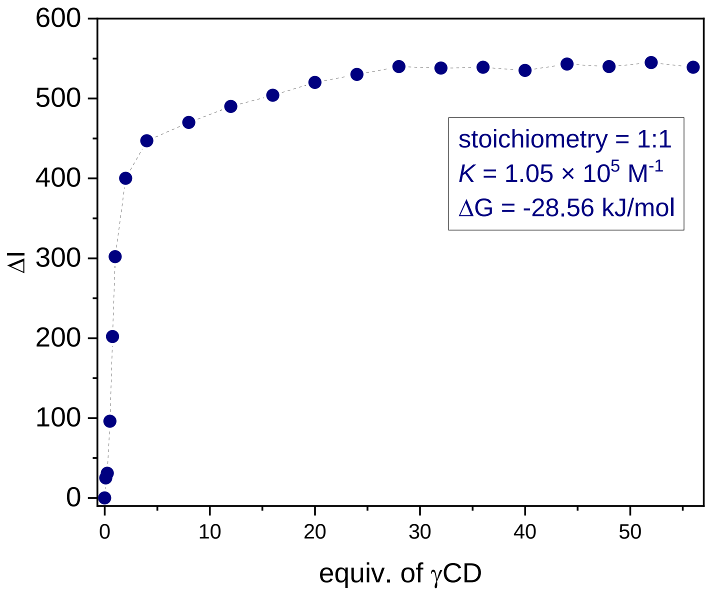

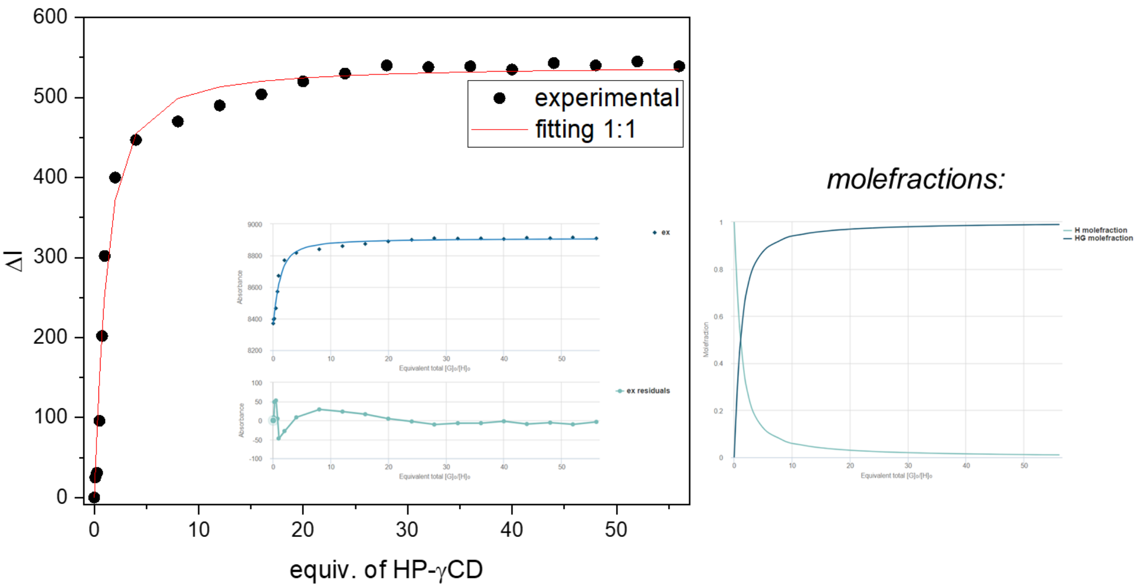

Fig. S26. (top) Fluorescence titration curve (DMSO:H_2O = 2:1 *vol/vol;* C_{sumenene} = 0.02 mM; λ_{em} = 379 nm) for supramolecular interactions between sumanene and HP-γCD, binding parameters are also presented in the frame; (bottom) Global fitting to the 1:1 binding model with Bindfit, K = 8.4±2.2 × 10^4 M^{-1}, the raw images from the Bindfit fitting are also presented.

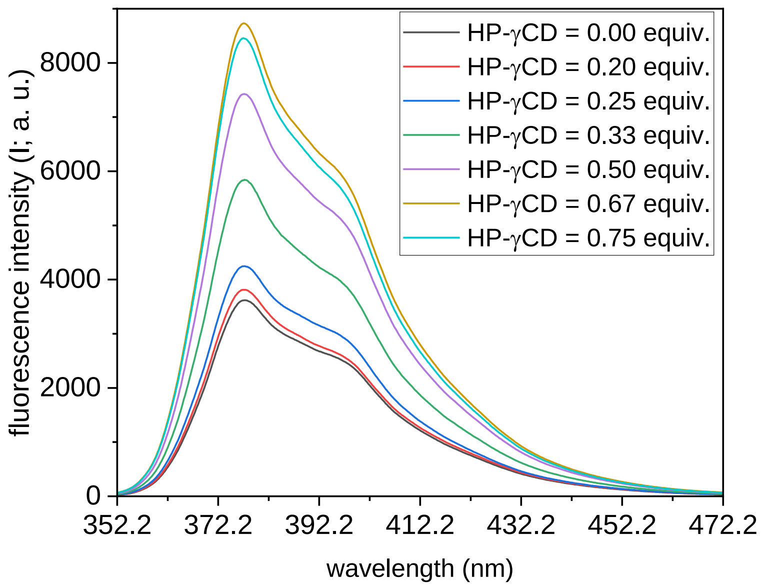

Fig. S27. Fluorescence spectra for Job's plot analyzes on supramolecular interactions between sumanene and HP-γCD (DMSO:H_2O = 2:1 *vol/vol;* λ_{ex} = 280 nm; λ_{em} = 379 nm).

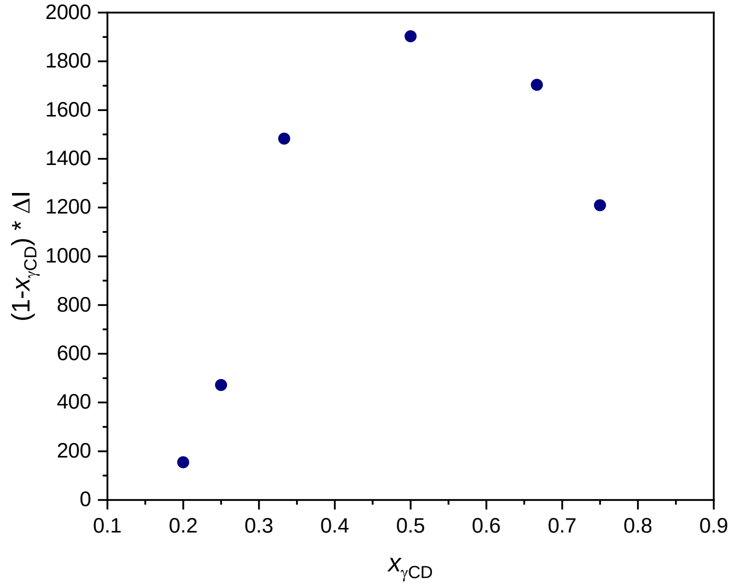

Fig. S28. Job's plot for supramolecular interactions between sumanene and HP-γCD probed with fluorescence spectroscopy (DMSO:H_2O = 2:1 *vol/vol;* λ_{ex} = 280 nm; λ_{em} = 379 nm).

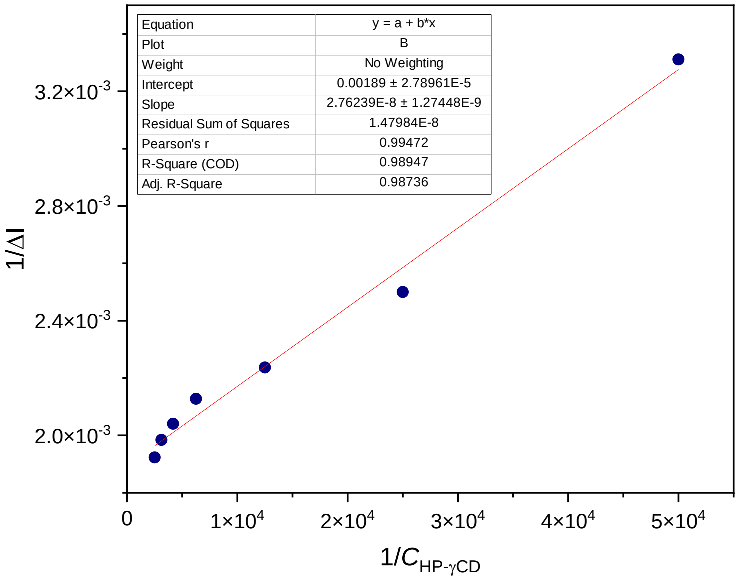

Fig. S29. Benesi-Hildebrand plot for supramolecular interactions between sumanene and HP-γCD probed with fluorescence spectroscopy (DMSO:H_2O = 2:1 *vol/vol;* $C_{sumenene}$ = 0.02 mM; λ_{em} = 379 nm). Linear regression parameters are also presented.

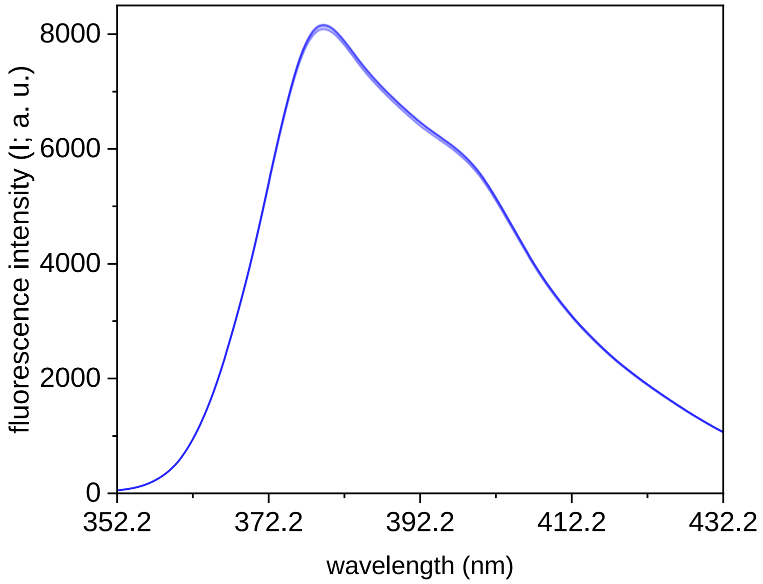

Fig. S30. Evolution of the fluorescence spectra titration (DMSO:H_2O = 2:1 *vol/vol;* $C_{sumenene}$ = 0.02 mM; λ_{ex} = 280 nm) for the reference spectra of sumanene in the presence of increasing molar equivalents of αCD (from 0 equiv. to 12 eqiuv.).

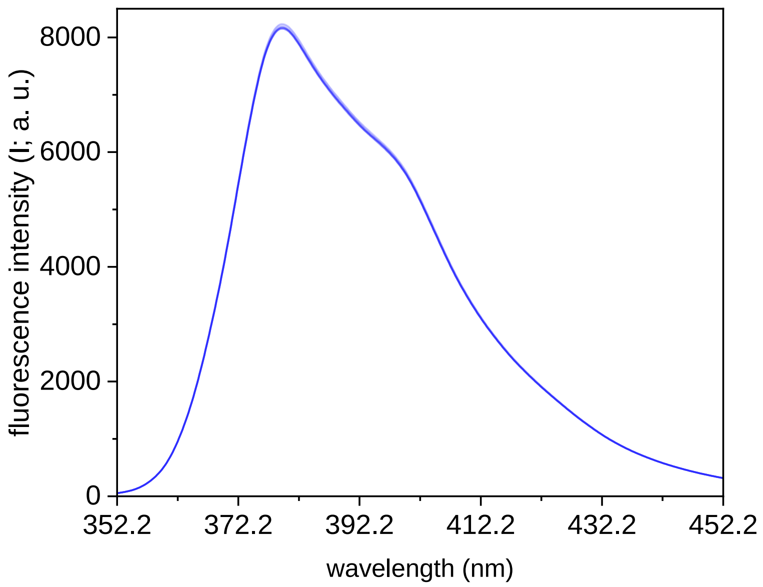

Fig. S31. Evolution of the fluorescence spectra titration (DMSO:H_2O = 2:1 *vol/vol; C*$_{sumenene}$ = 0.02 mM; λ_{ex} = 280 nm) for the reference spectra of sumanene in the presence of increasing molar equivalents of βCD (from 0 equiv. to 12 eqiuv.)

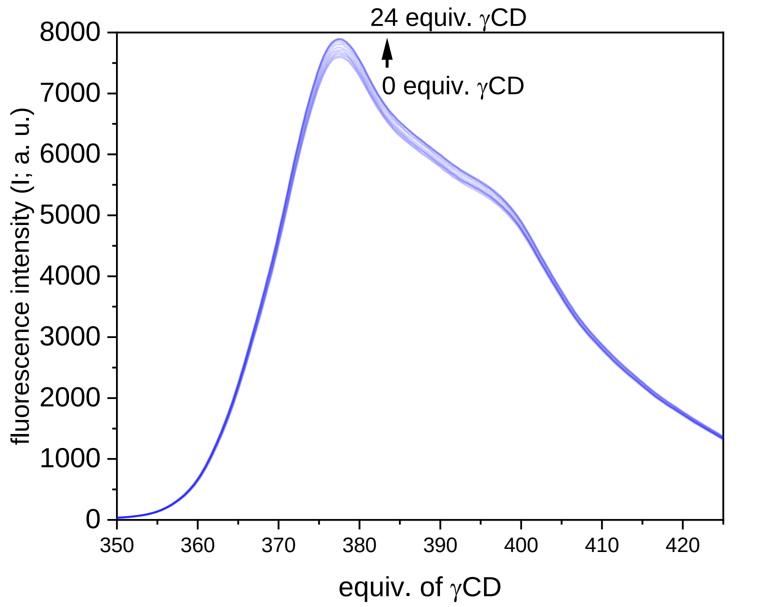

Fig. S32. Evolution of the fluorescence spectra titration (DMSO:H_2O = 4:1 *vol/vol; C*$_{sumenene}$ = 0.02 mM; λ_{ex} = 280 nm) on the supramolecular interactions between sumanene and γCD.

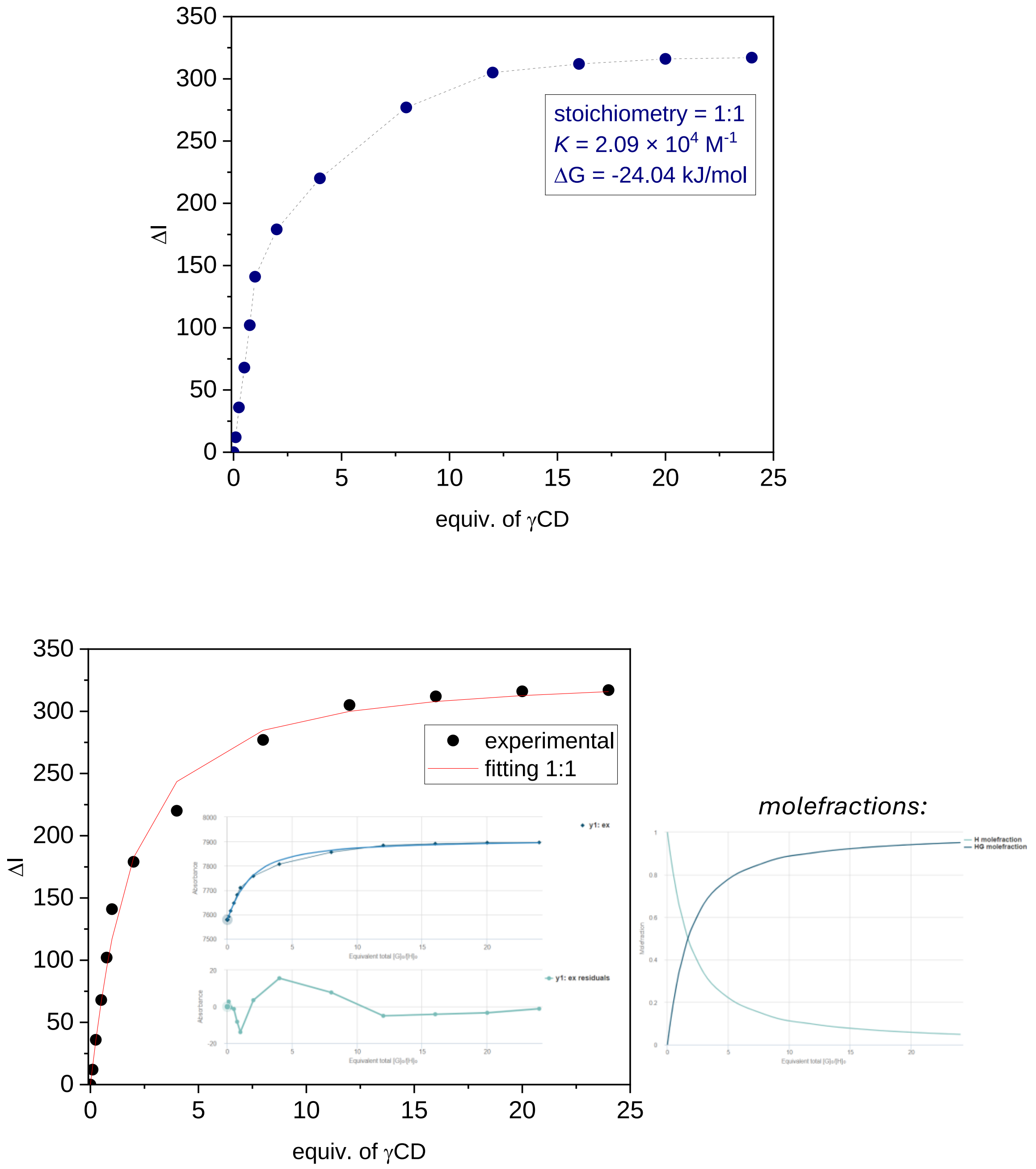

Fig. S33. (top) Fluorescence titration curve (DMSO:H_2O = 4:1 *vol/vol;* $C_{sumenene}$ = 0.02 mM; λ_{em} = 379 nm) for supramolecular interactions between sumanene and γCD, binding parameters are also presented in the frame; (bottom) Global fitting to the 1:1 binding model with Bindfit, $K = 3.2 \pm 0.3 \times 10^4$ M^{-1}, the raw images from the Bindfit fitting are also presented.

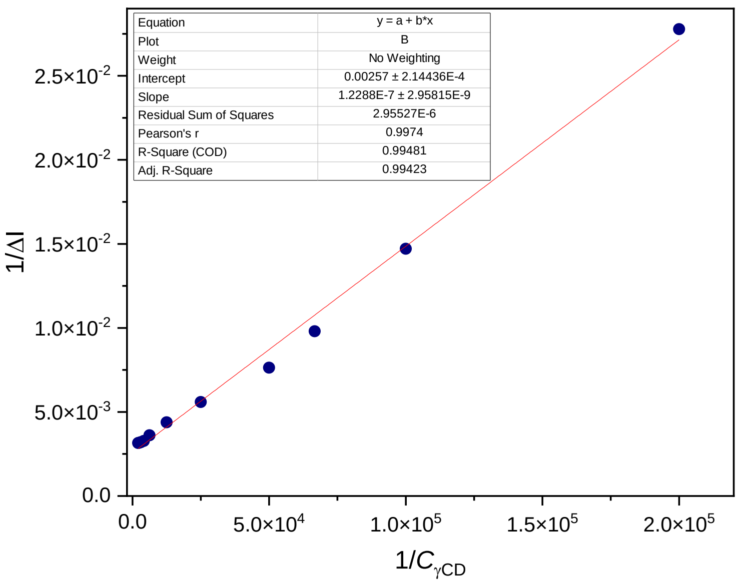

Fig. S34. Benesi-Hildebrand plot for supramolecular interactions between sumanene and γCD probed with fluorescence spectroscopy (DMSO:H_2O = 4:1 *vol/vol;* C_{sumenene} = 0.02 mM; λ_{em} = 379 nm). Linear regression parameters are also presented.

S4. Phase solubility studies on the supramolecular interactions

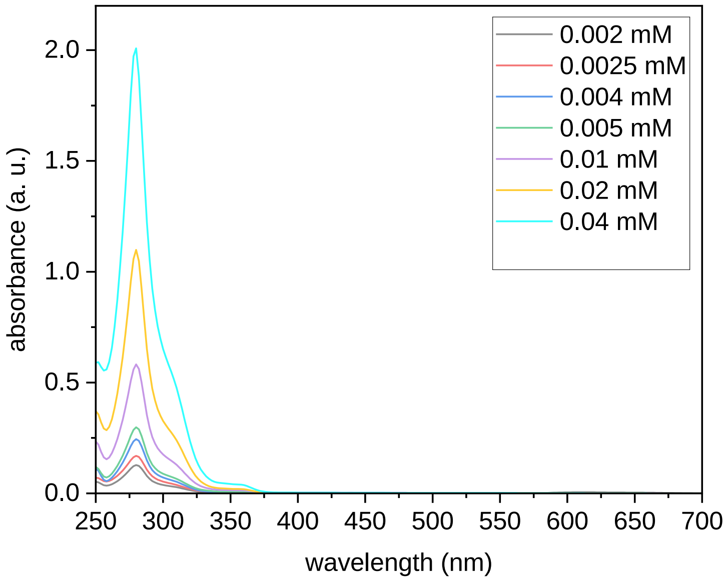

Fig. S35. UV-vis spectra (DMSO:H_2O = 90:10 *vol*/vol) of sumanene for the calibration curve for phase solubility studies.

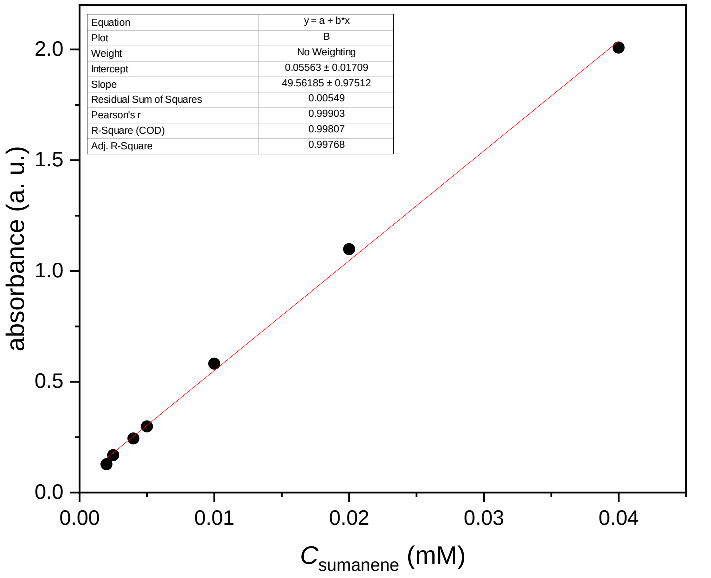

Fig. S36. Calibration curve for sumanene for phase solubility studies. Linear regression parameters are also presented (absorbance at 280 nm was taken for calculation).

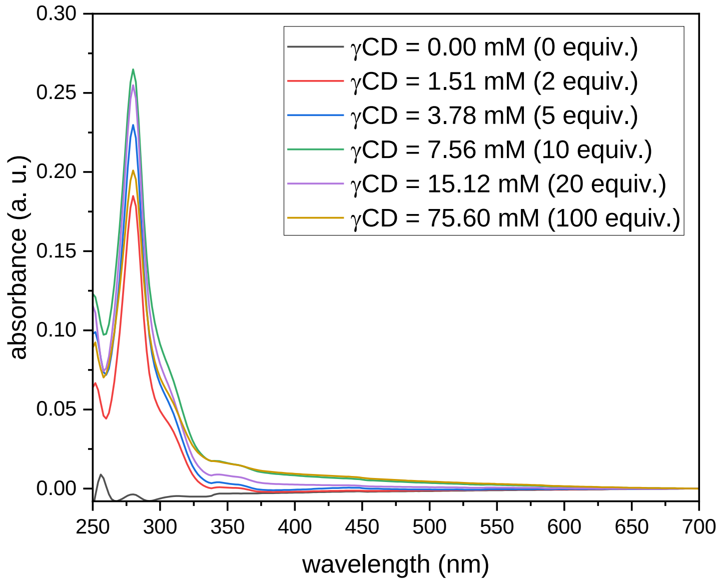

Fig. S37. UV-vis spectra (DMSO:H_2O = 90:10 *vol*/vol) for phase solubility studies with sumanene and γCD.

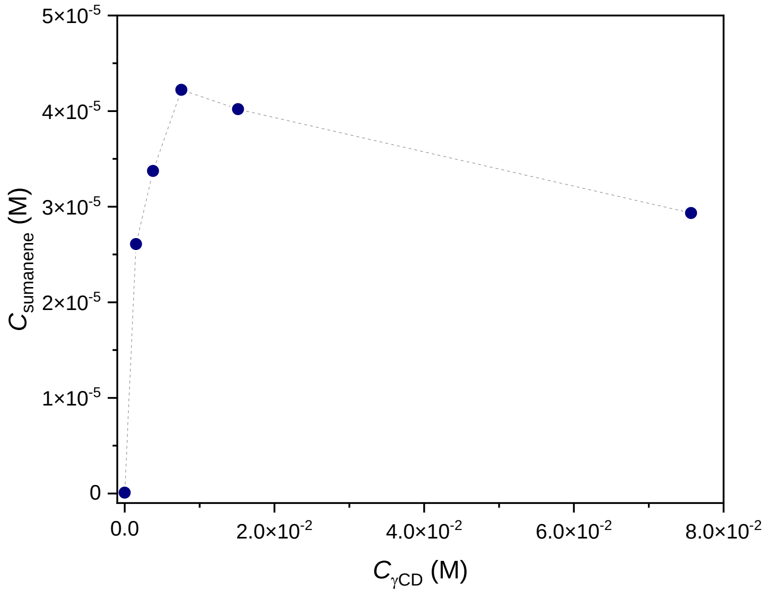

Fig. S38. Obtained phase solubility diagram for sumanene and γCD (absorbance at 280 nm was taken for calculation).

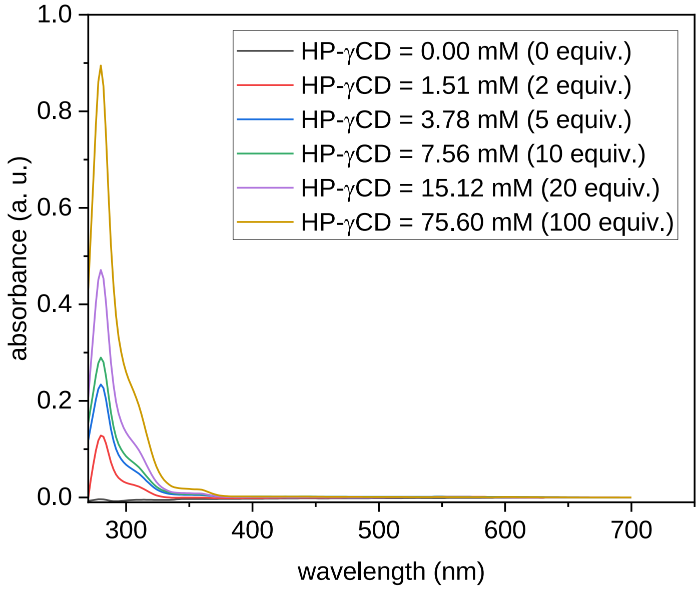

Fig. S39. UV-vis spectra (DMSO:H_2O = 90:10 *vol*/vol) for phase solubility studies with sumanene and HP-γCD.

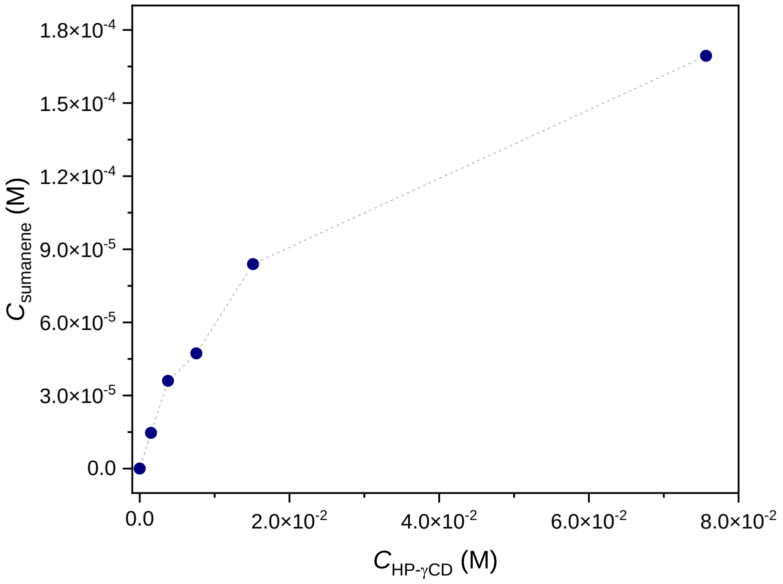

Fig. S40. Obtained phase solubility diagram for sumanene and HP-γCD (absorbance at 280 nm was taken for calculation).

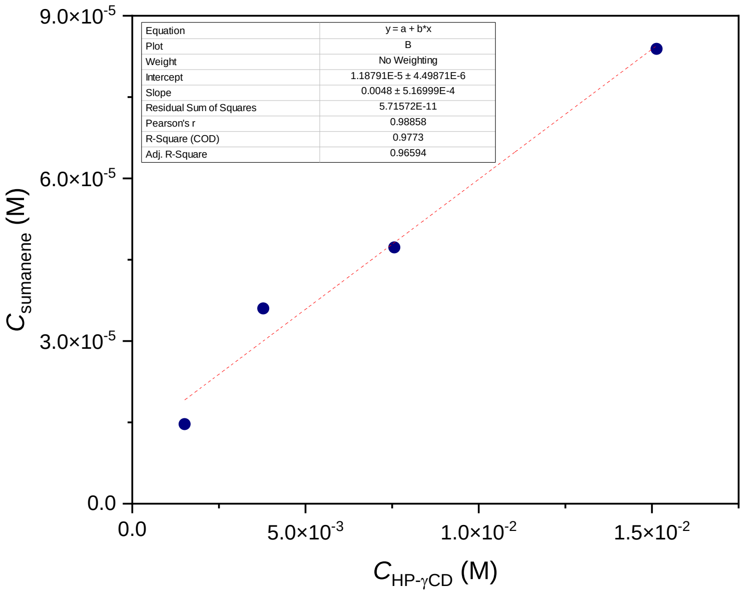

Fig. S41. Plot used for the estimation of binding constant (*K*) from phase solubility studies with sumanene and HP-γCD. Linear regression parameters are also presented.

S5. DFT computations

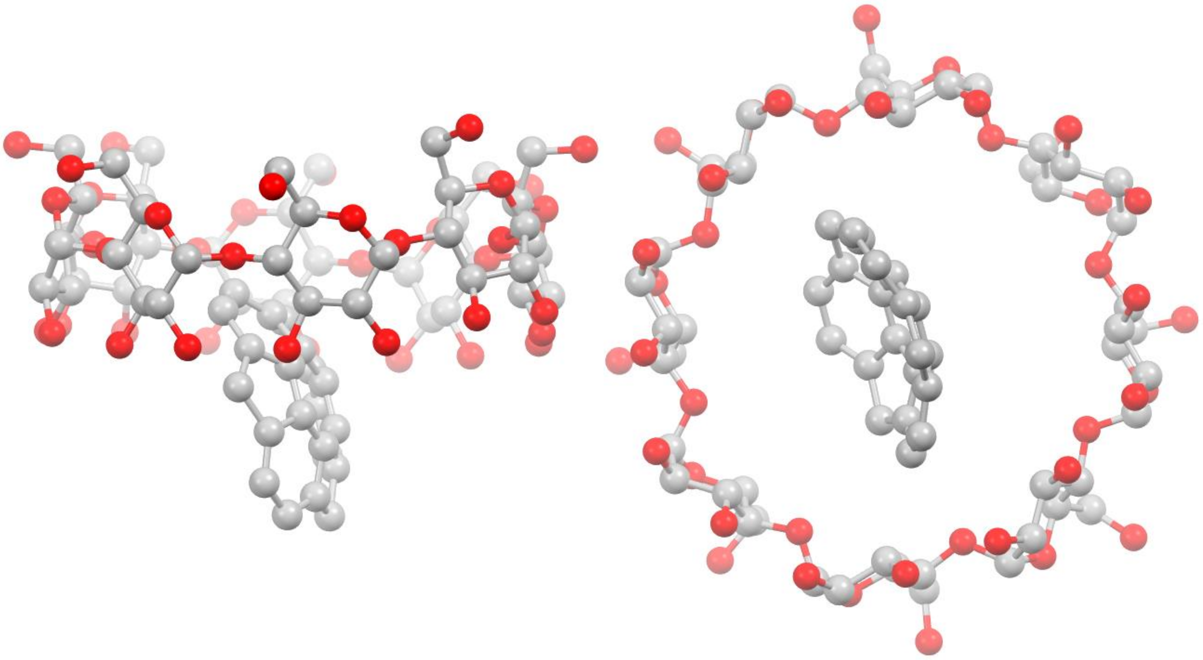

Fig. S42. DFT optimized (B3LYP/6-31g(d,p)) structure of 1:1 sumanene@γCD inclusion complex labelled as **sumanene@γCD_1** (views from two different perspectives are presented; hydrogen atoms are omitted for the clarity of the image).

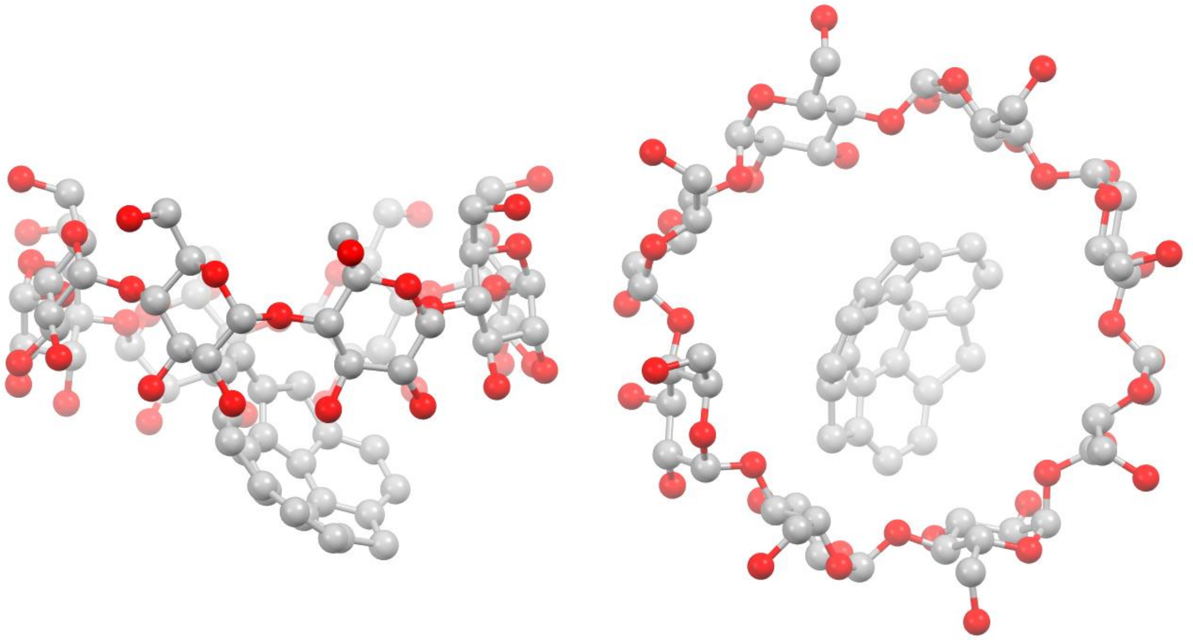

Fig. S43. DFT optimized (B3LYP/6-31g(d,p)) structure of 1:1 sumanene@γCD inclusion complex labelled as **sumanene@γCD_2** (views from two different perspectives are presented; hydrogen atoms are omitted for the clarity of the image).

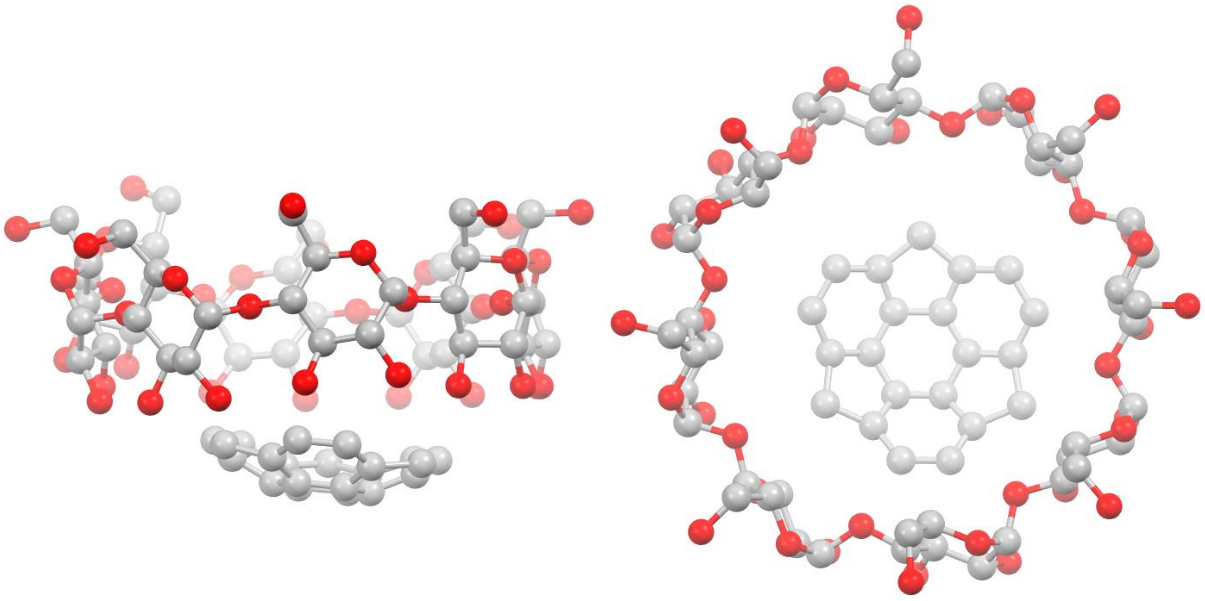

Fig. S44. DFT optimized (B3LYP/6-31g(d,p)) structure of 1:1 sumanene@γCD inclusion complex labelled as **sumanene@γCD_3** (views from two different perspectives are presented; hydrogen atoms are omitted for the clarity of the image).

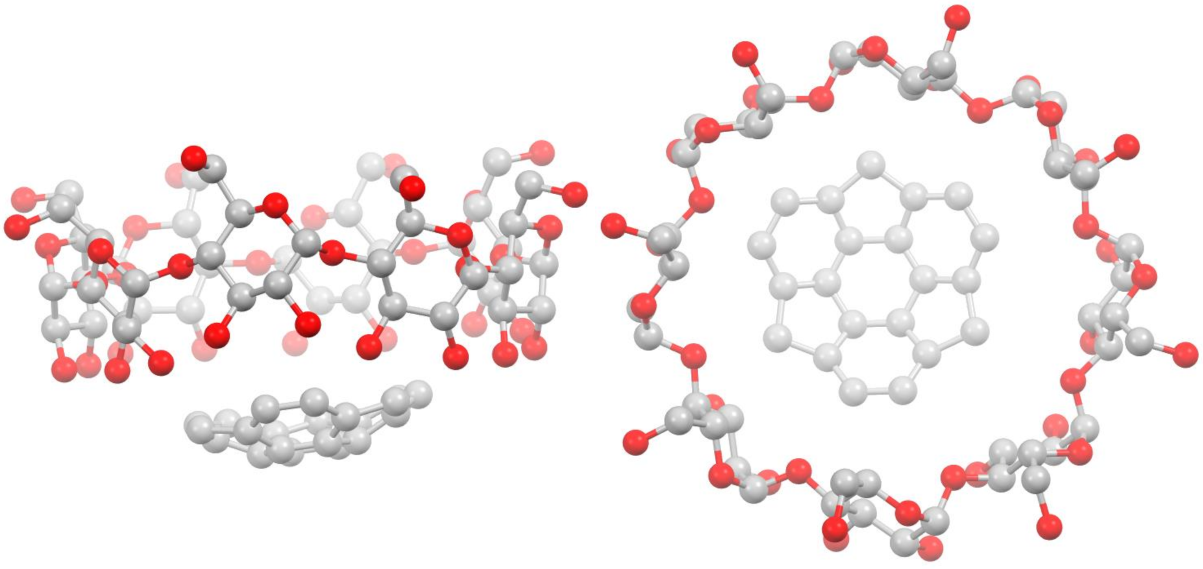

Fig. S45. DFT optimized (B3LYP/6-31g(d,p)) structure of 1:1 sumanene@γCD inclusion complex labelled as **sumanene@γCD_4** (views from two different perspectives are presented; hydrogen atoms are omitted for the clarity of the image).

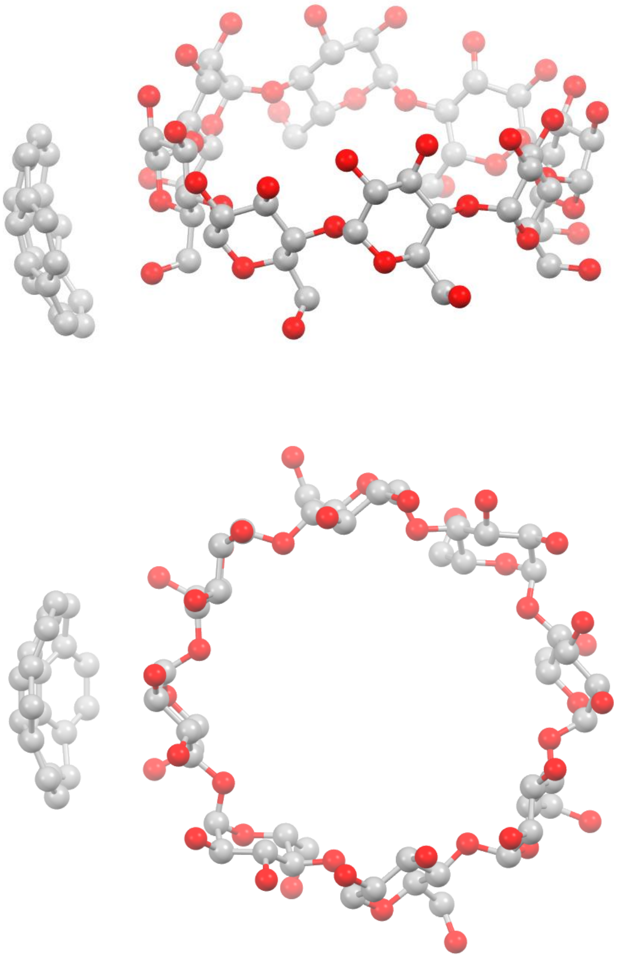

Fig. S46. DFT optimized (B3LYP/6-31g(d,p)) structure of 1:1 sumanene@γCD non-inclusion complex labelled as **sumanene@γCD_5** (views from two different perspectives are presented; hydrogen atoms are omitted for the clarity of the image).

Table S2. Summary of interaction energies for DFT-optimized structure (B3LYP/6-31g(d,p)) 1:1 sumanene@γCD complexes labelled as **sumanene@γCD_1-5**.

Note	Entry	Complex label	Sumanene orientation with regard to γCD cup	Interaction energy (kcal × mol^{-1})
inclusion complexes	1	sumanene@γCD_1	vertical	−10.20
	2	sumanene@γCD_2	vertical	−10.15
	3	sumanene@γCD_3	horizontal	−5.50
	4	sumanene@γCD_4	horizontal	−5.60
non-inclusion complex	5	sumanene@γCD_5	-	+0.41

Table S3. Atomic coordinates for the DFT-optimized structure (B3LYP/6-31g(d,p)) of 1:1 sumanene@γCD inclusion complex labelled as **sumanene@γCD_1**.

	x	*y*	*z*
C	1.8847000000	-6.6487000000	-1.0958000000
C	2.8098000000	-6.6465000000	0.1192000000
C	3.5157000000	-5.3095000000	0.3104000000
C	4.2679000000	-4.9372000000	-0.9622000000
C	3.3332000000	-4.9303000000	-2.1775000000
C	4.0761000000	-4.8250000000	-3.5044000000
C	6.2495000000	-3.4363000000	-0.7921000000
C	6.7080000000	-2.7822000000	0.5093000000
C	6.1909000000	-1.3550000000	0.6331000000
O	-0.9481000000	-7.6374000000	-3.5626000000
O	0.9002000000	7.8312000000	-3.5452000000
C	-0.8722000000	-6.1933000000	-3.4343000000
C	0.9049000000	6.3800000000	-3.4730000000
C	-1.3678000000	-5.7438000000	-2.0655000000
C	1.3481000000	5.9032000000	-2.0968000000
C	-0.5759000000	-6.3568000000	-0.9050000000
C	0.4876000000	6.4584000000	-0.9561000000
O	-2.7648000000	-6.2413000000	-2.0012000000
O	2.0595000000	-6.9724000000	1.3201000000
O	4.3772000000	-5.5175000000	1.4460000000
O	2.6338000000	-6.2363000000	-2.2584000000
O	4.9721000000	-5.9521000000	-3.6835000000
O	4.8199000000	-3.5915000000	-0.7857000000
O	6.2521000000	-3.5620000000	1.6480000000
O	6.7229000000	-0.8740000000	1.8860000000
O	5.9755000000	0.7747000000	-0.4919000000
O	6.6688000000	-2.6101000000	-1.8989000000
C	6.6314000000	-0.5327000000	-0.5727000000
C	6.2061000000	-1.2002000000	-1.8869000000
C	6.8684000000	-0.5814000000	-3.1126000000
O	2.7271000000	6.4307000000	-1.9438000000
O	0.7666000000	-5.7706000000	-0.8735000000
O	-0.8450000000	5.8546000000	-1.0096000000
O	-0.6554000000	-6.7914000000	1.5345000000
O	0.4318000000	6.7764000000	1.5024000000
O	-3.3514000000	-6.2058000000	1.6482000000
O	3.1309000000	6.2236000000	1.7257000000
O	-3.6973000000	-4.5756000000	-0.5991000000
O	8.3107000000	-0.7231000000	-3.0452000000
O	3.5857000000	4.6963000000	-0.5839000000
O	-6.5978000000	-5.1605000000	-2.7966000000
O	6.5959000000	5.3492000000	-2.6058000000

C	-1.9507000000	6.6999000000	-1.3767000000
C	-2.9213000000	6.8258000000	-0.2052000000
C	-3.6040000000	5.5025000000	0.1160000000
C	-4.3069000000	4.9624000000	-1.1268000000
C	-3.3233000000	4.8345000000	-2.2962000000
C	-4.0005000000	4.5510000000	-3.6315000000
C	-6.2146000000	3.5157000000	-0.4719000000
C	-6.3586000000	2.7980000000	0.8707000000
C	-5.8723000000	1.3543000000	0.7940000000
O	-6.7963000000	-2.6853000000	-1.5048000000
O	6.7135000000	2.8276000000	-1.4141000000
O	-4.9938000000	-4.0356000000	2.0028000000
O	4.7724000000	4.0417000000	2.0522000000
O	-6.6622000000	-1.8535000000	2.0933000000
O	6.3485000000	1.7820000000	2.1161000000
O	-8.8299000000	0.8518000000	-2.2774000000
C	-1.2452000000	-6.0717000000	0.4342000000
C	1.0926000000	6.1222000000	0.4006000000
O	-2.2166000000	7.3007000000	0.9750000000
O	-4.5113000000	5.8111000000	1.1950000000
O	-2.6513000000	6.1416000000	-2.5069000000
O	-4.8895000000	5.6346000000	-4.0030000000
O	-4.8298000000	3.6244000000	-0.8375000000
O	-5.6243000000	3.4987000000	1.9080000000
O	-6.0870000000	0.8142000000	2.1176000000
O	-6.0602000000	-0.7186000000	-0.4238000000
O	-6.9457000000	2.7584000000	-1.4647000000
C	-6.6381000000	0.6205000000	-0.2999000000
C	-6.5150000000	1.3516000000	-1.6434000000
C	-7.4450000000	0.7981000000	-2.7145000000
C	6.7978000000	1.9352000000	-0.2821000000
C	-6.9380000000	-1.8546000000	-0.3329000000
C	6.3527000000	2.6824000000	0.9752000000
C	-6.5806000000	-2.6824000000	0.8996000000
C	4.9746000000	3.3180000000	0.8232000000
C	-5.1809000000	-3.2763000000	0.7901000000
C	4.9551000000	4.2099000000	-0.4117000000
C	-5.0818000000	-4.1109000000	-0.4793000000
C	5.3835000000	3.4377000000	-1.6643000000
C	-5.4525000000	-3.2812000000	-1.7156000000
C	5.5970000000	4.3308000000	-2.8794000000
C	-5.6009000000	-4.1193000000	-2.9782000000
C	3.3869000000	6.1165000000	-0.6982000000
C	-3.4830000000	-5.9889000000	-0.7770000000
C	2.5321000000	6.6233000000	0.4615000000
C	-2.6912000000	-6.5548000000	0.3994000000

H	-4.8028000000	1.3407000000	0.5495000000
H	-7.4246000000	2.7919000000	1.1319000000
H	-9.0567000000	1.7904000000	-2.1086000000
H	-5.4734000000	1.3298000000	-1.9819000000
H	-1.5896000000	7.6716000000	-1.7202000000
H	5.8339000000	2.2141000000	2.8318000000
H	-6.2973000000	-2.3854000000	2.8336000000
H	-3.6917000000	7.5586000000	-0.4769000000
H	4.0718000000	4.7426000000	1.9624000000
H	-4.3154000000	-4.7572000000	1.8944000000
H	4.2169000000	2.5310000000	0.7063000000
H	-4.4500000000	-2.4603000000	0.7318000000
H	-4.3606000000	6.4602000000	-4.0198000000
H	5.6327000000	5.0593000000	-0.2707000000
H	-5.7530000000	-4.9748000000	-0.4188000000
H	4.6573000000	2.6471000000	-1.8834000000
H	-4.7210000000	-2.4793000000	-1.8609000000
H	-3.2338000000	4.3884000000	-4.4003000000
H	-4.6238000000	3.6597000000	-3.5503000000
H	2.4912000000	6.4636000000	2.4308000000
H	-2.7490000000	-6.4825000000	2.3727000000
H	-6.2453000000	-0.1694000000	2.1056000000
H	4.3482000000	6.6320000000	-0.7510000000
H	-4.4396000000	-6.4998000000	-0.9041000000
H	2.5107000000	7.7193000000	0.4105000000
H	-2.6773000000	-7.6476000000	0.3009000000
H	1.0788000000	5.0316000000	0.5321000000
H	-1.2194000000	-4.9888000000	0.6187000000
H	-6.6874000000	4.4997000000	-0.4578000000
H	-5.5485000000	2.8957000000	2.6782000000
H	-2.5698000000	4.0731000000	-2.0676000000
H	-5.1300000000	5.6226000000	-1.4237000000
H	-2.8492000000	4.7714000000	0.4348000000
H	-4.8836000000	4.9777000000	1.5966000000
H	-2.8501000000	7.2161000000	1.7221000000
H	6.6708000000	0.4912000000	-3.1423000000
H	6.4672000000	-1.0506000000	-4.0200000000
H	7.7199000000	-0.4031000000	-0.5744000000
H	6.5396000000	0.0997000000	2.0184000000
H	4.7060000000	-3.9343000000	-3.5116000000
H	3.3495000000	-4.7633000000	-4.3248000000
H	4.4338000000	-6.7709000000	-3.6491000000
H	3.5752000000	-7.4167000000	-0.0400000000
H	1.5327000000	-7.6546000000	-1.3341000000
H	5.1145000000	-1.1772000000	-1.9752000000
H	8.5146000000	-1.6802000000	-2.9843000000

H	5.0950000000	-1.3706000000	0.6581000000
H	7.8050000000	-2.7602000000	0.5103000000
H	6.7375000000	-4.3991000000	-0.9575000000
H	6.4096000000	-3.0156000000	2.4482000000
H	2.5943000000	-4.1279000000	-2.0755000000
H	5.0817000000	-5.6482000000	-1.1452000000
H	2.7691000000	-4.5300000000	0.5188000000
H	-7.6985000000	0.5408000000	-0.0352000000
H	1.8103000000	8.1470000000	-3.3632000000
H	-1.8838000000	-7.9026000000	-3.4388000000
H	1.3716000000	4.8089000000	-2.0518000000
H	-1.3681000000	-4.6509000000	-1.9879000000
H	-7.3027000000	1.3621000000	-3.6445000000
H	-7.2264000000	-0.2559000000	-2.8945000000
H	7.8506000000	1.6490000000	-0.2273000000
H	-7.9827000000	-1.5362000000	-0.3312000000
H	7.0771000000	3.4853000000	1.1609000000
H	-7.3058000000	-3.5006000000	0.9884000000
H	7.4342000000	4.8962000000	-2.3753000000
H	-7.4544000000	-4.7287000000	-2.5950000000
H	5.8890000000	3.7127000000	-3.7379000000
H	-5.8618000000	-3.4650000000	-3.8194000000
H	4.6770000000	4.8672000000	-3.1171000000
H	-4.6646000000	-4.6344000000	-3.1992000000
H	-0.5492000000	6.8548000000	1.3564000000
H	0.3394000000	-6.7538000000	1.5173000000
H	5.0020000000	-4.7548000000	1.5884000000
H	2.6581000000	-6.8006000000	2.0798000000
H	0.4038000000	7.5446000000	-1.0720000000
H	-0.4997000000	-7.4382000000	-1.0650000000
H	1.5651000000	5.9493000000	-4.2365000000
H	-1.4606000000	-5.6955000000	-4.2156000000
H	-0.1221000000	6.0657000000	-3.6663000000
H	0.1809000000	-5.9350000000	-3.5553000000
C	-0.1034000000	-1.7032000000	0.4764000000
C	3.5985000000	-0.0564000000	3.4899000000
C	-1.4529000000	-1.7776000000	0.9643000000
C	-1.8891000000	-0.9609000000	2.0201000000
C	-0.9778000000	0.0075000000	2.4731000000
C	-3.0138000000	-1.0643000000	3.1008000000
C	2.6101000000	-0.0154000000	2.4929000000
C	-2.4885000000	-0.4150000000	5.7088000000
C	2.3796000000	-0.7723000000	1.1450000000
C	3.3012000000	0.3307000000	4.8408000000
C	2.0173000000	0.7617000000	5.2083000000
C	0.8229000000	-0.8098000000	1.0350000000

C	0.3244000000	0.0817000000	1.9987000000
C	-2.3860000000	-0.3083000000	4.3136000000
C	-1.2716000000	0.3928000000	3.8251000000
C	1.3770000000	0.5475000000	2.8581000000
C	-1.4355000000	0.0477000000	6.5689000000
C	1.2034000000	0.7722000000	6.5415000000
C	1.0922000000	0.9208000000	4.1644000000
C	-0.2698000000	0.6252000000	6.0434000000
C	-0.2546000000	0.8412000000	4.6562000000
H	0.2109000000	-2.4552000000	-0.2433000000
H	4.5706000000	-0.4997000000	3.2929000000
H	-2.0821000000	-2.5729000000	0.5739000000
H	-3.2597000000	-2.1060000000	3.3342000000
H	-3.9421000000	-0.5767000000	2.7704000000
H	-3.3067000000	-0.9637000000	6.1671000000
H	2.8053000000	-1.7819000000	1.1679000000
H	2.8435000000	-0.2481000000	0.2971000000
H	4.0659000000	0.1552000000	5.5929000000
H	-1.5165000000	-0.1784000000	7.6287000000
H	1.3568000000	1.6993000000	7.1116000000
H	1.4921000000	-0.0582000000	7.1956000000

Table S4. Atomic coordinates for the DFT-optimized structure (B3LYP/6-31g(d,p)) of 1:1 sumanene@γCD inclusion complex labelled as **sumanene@γCD_2**.

	x	*y*	*z*
C	3.9546000000	-5.5420000000	-1.6941000000
C	5.0197000000	-5.1793000000	-0.6628000000
C	5.1646000000	-3.6723000000	-0.4935000000
C	5.4587000000	-3.0223000000	-1.8409000000
C	4.3924000000	-3.3983000000	-2.8787000000
C	4.7757000000	-3.0039000000	-4.2997000000
C	6.6379000000	-0.8228000000	-1.8655000000
C	7.0139000000	-0.0689000000	-0.5919000000
C	5.9753000000	0.9885000000	-0.2370000000
O	1.5202000000	-7.9195000000	-3.3079000000
O	-2.9113000000	7.5546000000	-3.2047000000
C	0.9580000000	-6.5799000000	-3.3360000000
C	-2.1805000000	6.3102000000	-3.3845000000
C	0.5379000000	-6.1334000000	-1.9429000000
C	-1.5052000000	5.8733000000	-2.0935000000
C	1.6791000000	-6.1873000000	-0.9197000000
C	-2.4691000000	5.7836000000	-0.9013000000
O	-0.5066000000	-7.1059000000	-1.5344000000
O	4.6893000000	-5.7789000000	0.6213000000
O	6.2274000000	-3.5180000000	0.4710000000
O	4.2621000000	-4.8761000000	-2.9348000000
O	5.9978000000	-3.6713000000	-4.7087000000
O	5.4286000000	-1.5695000000	-1.6564000000
O	7.1654000000	-0.9948000000	0.5173000000
O	6.4757000000	1.6058000000	0.9680000000
O	4.7485000000	2.9004000000	-1.1086000000
O	6.4648000000	0.1336000000	-2.9321000000
C	5.8332000000	1.9624000000	-1.4038000000
C	5.4811000000	1.2217000000	-2.7009000000
C	5.6041000000	2.0960000000	-3.9437000000
O	-0.5012000000	6.9282000000	-1.8058000000
O	2.6546000000	-5.1375000000	-1.2217000000
O	-3.4426000000	4.7181000000	-1.1243000000
O	2.1417000000	-6.1445000000	1.5225000000
O	-2.5069000000	5.4554000000	1.5732000000
O	-0.5050000000	-6.6123000000	2.1286000000
O	0.0830000000	6.3446000000	1.8003000000
O	-1.7651000000	-5.6321000000	-0.1930000000
O	6.9703000000	2.5486000000	-4.1231000000
O	1.0782000000	5.5391000000	-0.7210000000
O	-4.4229000000	-7.9404000000	-0.8449000000
O	3.5148000000	7.5393000000	-2.6996000000

C	-4.7879000000	5.1402000000	-1.4546000000
C	-5.7081000000	4.9707000000	-0.2495000000
C	-5.8939000000	3.5099000000	0.1364000000
C	-6.3676000000	2.6961000000	-1.0643000000
C	-5.4432000000	2.8897000000	-2.2730000000
C	-6.0262000000	2.3372000000	-3.5681000000
C	-7.5713000000	0.5833000000	-0.4992000000
C	-7.6179000000	-0.0161000000	0.9049000000
C	-6.5251000000	-1.0605000000	1.0934000000
O	-5.4936000000	-5.4078000000	-0.0683000000
O	4.6311000000	5.1657000000	-1.7880000000
O	-2.3103000000	-4.5727000000	2.5101000000
O	2.5910000000	5.2706000000	1.8127000000
O	-4.7185000000	-3.2862000000	2.8598000000
O	4.9048000000	3.6804000000	1.5803000000
O	-8.4921000000	-3.0173000000	-2.1891000000
C	1.1574000000	-5.9390000000	0.4893000000
C	-1.6964000000	5.4854000000	0.3765000000
O	-5.1680000000	5.6980000000	0.8945000000
O	-6.8464000000	3.5419000000	1.2177000000
O	-5.2859000000	4.3411000000	-2.5394000000
O	-7.2641000000	3.0135000000	-3.9072000000
O	-6.3304000000	1.2779000000	-0.6977000000
O	-7.4651000000	1.0184000000	1.9123000000
O	-6.6958000000	-1.5371000000	2.4446000000
O	-5.5087000000	-3.0485000000	0.1283000000
O	-7.7048000000	-0.4930000000	-1.4536000000
C	-6.6689000000	-2.1578000000	0.0426000000
C	-6.6892000000	-1.5715000000	-1.3752000000
C	-7.1335000000	-2.5737000000	-2.4341000000
C	5.1037000000	4.2520000000	-0.7742000000
C	-5.6626000000	-4.2895000000	0.8347000000
C	4.4977000000	4.6448000000	0.5759000000
C	-4.6101000000	-4.4031000000	1.9389000000
C	2.9769000000	4.7452000000	0.5247000000
C	-3.1927000000	-4.4592000000	1.3701000000
C	2.5366000000	5.6504000000	-0.6223000000
C	-3.1014000000	-5.6292000000	0.3989000000
C	3.1604000000	5.2313000000	-1.9561000000
C	-4.1588000000	-5.5012000000	-0.7059000000
C	2.9493000000	6.2531000000	-3.0663000000
C	-4.2351000000	-6.7199000000	-1.6134000000
C	0.2827000000	6.7291000000	-0.6062000000
C	-1.0767000000	-6.9033000000	-0.2280000000
C	-0.6688000000	6.5989000000	0.5829000000
C	0.0398000000	-6.9245000000	0.8123000000

H	-5.5443000000	-0.5834000000	0.9681000000
H	-8.5955000000	-0.4969000000	1.0352000000
H	-9.0670000000	-2.2230000000	-2.1785000000
H	-5.7036000000	-1.1579000000	-1.6144000000
H	-4.7785000000	6.1657000000	-1.8305000000
H	4.3371000000	3.7598000000	2.3777000000
H	-3.9159000000	-3.2846000000	3.4237000000
H	-6.6881000000	5.3932000000	-0.5032000000
H	1.6525000000	5.6023000000	1.8125000000
H	-1.6044000000	-5.2614000000	2.3652000000
H	2.5521000000	3.7453000000	0.3632000000
H	-2.9777000000	-3.5261000000	0.8360000000
H	-7.0718000000	3.9726000000	-3.9747000000
H	2.8154000000	6.6890000000	-0.4113000000
H	-3.2652000000	-6.5704000000	0.9347000000
H	2.7830000000	4.2465000000	-2.2521000000
H	-3.9744000000	-4.5962000000	-1.2952000000
H	-5.2869000000	2.4395000000	-4.3729000000
H	-6.2808000000	1.2837000000	-3.4432000000
H	-0.5716000000	6.1145000000	2.4953000000
H	0.2606000000	-6.5550000000	2.7421000000
H	-5.9326000000	-2.1188000000	2.7159000000
H	0.9184000000	7.6139000000	-0.5314000000
H	-1.7870000000	-7.7219000000	-0.0933000000
H	-1.2043000000	7.5507000000	0.6934000000
H	0.4661000000	-7.9348000000	0.8389000000
H	-1.1784000000	4.5248000000	0.2765000000
H	0.7614000000	-4.9157000000	0.5382000000
H	-8.4192000000	1.2440000000	-0.6923000000
H	-7.2653000000	0.5565000000	2.7566000000
H	-4.4611000000	2.4532000000	-2.0638000000
H	-7.3882000000	2.9816000000	-1.3441000000
H	-4.9299000000	3.1013000000	0.4698000000
H	-7.0356000000	2.6276000000	1.5674000000
H	-5.7511000000	5.4776000000	1.6550000000
H	4.9997000000	2.9965000000	-3.8276000000
H	5.2558000000	1.5344000000	-4.8202000000
H	6.7675000000	2.5159000000	-1.5550000000
H	5.8684000000	2.3360000000	1.2813000000
H	4.9845000000	-1.9340000000	-4.3473000000
H	3.9481000000	-3.2431000000	-4.9798000000
H	5.8483000000	-4.6380000000	-4.6412000000
H	5.9799000000	-5.5852000000	-1.0049000000
H	3.9577000000	-6.6073000000	-1.9342000000
H	4.4774000000	0.7907000000	-2.6194000000
H	7.5414000000	1.7538000000	-4.1823000000

H	5.0060000000	0.5063000000	-0.0558000000
H	7.9756000000	0.4303000000	-0.7660000000
H	7.4419000000	-1.4812000000	-2.2023000000
H	7.2198000000	-0.4501000000	1.3322000000
H	3.4293000000	-2.9613000000	-2.5942000000
H	6.4434000000	-3.3293000000	-2.2122000000
H	4.2266000000	-3.2531000000	-0.1066000000
H	-7.5889000000	-2.7307000000	0.2073000000
H	-2.2735000000	8.2552000000	-2.9542000000
H	0.8333000000	-8.5319000000	-2.9702000000
H	-0.9924000000	4.9154000000	-2.2340000000
H	0.1044000000	-5.1280000000	-1.9700000000
H	-7.0402000000	-2.1182000000	-3.4284000000
H	-6.5092000000	-3.4670000000	-2.3863000000
H	6.1894000000	4.3711000000	-0.7713000000
H	-6.6765000000	-4.3878000000	1.2277000000
H	4.8927000000	5.6329000000	0.8441000000
H	-4.8012000000	-5.3351000000	2.4863000000
H	4.4731000000	7.4108000000	-2.5367000000
H	-5.2775000000	-7.8780000000	-0.3695000000
H	3.3947000000	5.8770000000	-3.9959000000
H	-5.0476000000	-6.5813000000	-2.3370000000
H	1.8828000000	6.4254000000	-3.2206000000
H	-3.2934000000	-6.8467000000	-2.1503000000
H	-3.4791000000	5.4837000000	1.3586000000
H	3.0648000000	-5.9299000000	1.2160000000
H	6.5291000000	-2.5699000000	0.5485000000
H	5.3292000000	-5.4182000000	1.2734000000
H	-2.9943000000	6.7399000000	-0.8037000000
H	2.1635000000	-7.1681000000	-0.9792000000
H	-1.4254000000	6.4076000000	-4.1741000000
H	0.0934000000	-6.5285000000	-4.0095000000
H	-2.9221000000	5.5690000000	-3.6873000000
H	1.7484000000	-5.9297000000	-3.7150000000
C	2.2531000000	2.4448000000	5.5091000000
C	2.7182000000	-2.4384000000	4.2043000000
C	1.3096000000	3.2848000000	4.8223000000
C	0.1881000000	2.7363000000	4.1757000000
C	-0.0275000000	1.3636000000	4.3629000000
C	-0.7074000000	3.1781000000	2.9768000000
C	2.3140000000	-1.2983000000	4.9187000000
C	-1.5217000000	1.3747000000	1.0893000000
C	3.0576000000	-0.1658000000	5.6987000000
C	1.8966000000	-2.9894000000	3.1627000000
C	0.6703000000	-2.3989000000	2.8129000000
C	2.0871000000	1.0494000000	5.5508000000

C	0.8868000000	0.5525000000	5.0229000000
C	-1.1793000000	1.8156000000	2.3779000000
C	-0.8367000000	0.8214000000	3.3069000000
C	1.0189000000	-0.8285000000	4.6542000000
C	-1.3926000000	-0.0089000000	0.7249000000
C	-0.1974000000	-2.3473000000	1.5134000000
C	0.2312000000	-1.3537000000	3.6385000000
C	-0.9177000000	-0.9650000000	1.6386000000
C	-0.7091000000	-0.5139000000	2.9514000000
H	3.1577000000	2.9098000000	5.8926000000
H	3.7118000000	-2.8604000000	4.3327000000
H	1.5493000000	4.3389000000	4.7142000000
H	-0.1179000000	3.7475000000	2.2523000000
H	-1.5488000000	3.8104000000	3.2934000000
H	-1.7721000000	2.0882000000	0.3074000000
H	4.0290000000	0.0626000000	5.2433000000
H	3.2411000000	-0.4305000000	6.7492000000
H	2.3009000000	-3.8186000000	2.5894000000
H	-1.5535000000	-0.2691000000	-0.3182000000
H	-0.9035000000	-3.1866000000	1.4377000000
H	0.4337000000	-2.3841000000	0.6173000000

Table S5. Atomic coordinates for the DFT-optimized structure ((B3LYP/6-31g(d,p)) of 1:1 sumanene@γCD inclusion complex labelled as **sumanene@γCD_3**.

	x	*y*	*z*
C	3.8903000000	-5.9113000000	-0.4355000000
C	4.3111000000	-5.2550000000	0.8801000000
C	4.5212000000	-3.7524000000	0.7270000000
C	5.5467000000	-3.4975000000	-0.3700000000
C	5.1258000000	-4.1665000000	-1.6846000000
C	6.2099000000	-4.1279000000	-2.7526000000
C	6.9537000000	-1.4570000000	-0.5440000000
C	7.0902000000	-0.5360000000	0.6658000000
C	6.1745000000	0.6792000000	0.5766000000
O	1.7532000000	-7.4017000000	-3.9353000000
O	-1.8629000000	7.5837000000	-3.6358000000
C	1.4407000000	-6.0199000000	-3.6259000000
C	-1.4041000000	6.2052000000	-3.6361000000
C	0.7205000000	-5.9092000000	-2.2879000000
C	-0.7100000000	5.8623000000	-2.3248000000
C	1.5545000000	-6.4114000000	-1.1028000000
C	-1.5961000000	6.0940000000	-1.0941000000
O	-0.4688000000	-6.7847000000	-2.4483000000
O	3.3225000000	-5.4997000000	1.9181000000
O	4.9428000000	-3.2958000000	2.0309000000
O	4.8974000000	-5.6088000000	-1.4286000000
O	7.4404000000	-4.7319000000	-2.2689000000
O	5.6411000000	-2.0502000000	-0.5623000000
O	6.7929000000	-1.2798000000	1.8821000000
O	6.4912000000	1.4639000000	1.7470000000
O	5.4174000000	2.4827000000	-0.8336000000
O	7.1665000000	-0.6884000000	-1.7451000000
C	6.4175000000	1.4182000000	-0.7341000000
C	6.2694000000	0.4783000000	-1.9371000000
C	6.7337000000	1.1031000000	-3.2461000000
O	0.4456000000	6.7916000000	-2.2606000000
O	2.6062000000	-5.4221000000	-0.8553000000
O	-2.6777000000	5.1092000000	-1.0764000000
O	1.3854000000	-7.2011000000	1.2422000000
O	-1.5131000000	6.2825000000	1.3861000000
O	-1.3415000000	-7.4874000000	1.0728000000
O	1.1849000000	6.7369000000	1.3638000000
O	-1.8905000000	-5.6058000000	-0.9992000000
O	8.1250000000	1.5110000000	-3.1614000000
O	1.9409000000	5.4326000000	-1.0179000000
O	-4.5709000000	-7.1992000000	-2.7075000000
O	4.3160000000	6.4908000000	-3.7014000000

C	-4.0194000000	5.5892000000	-1.2883000000
C	-4.8591000000	5.3620000000	-0.0345000000
C	-5.0887000000	3.8826000000	0.2452000000
C	-5.7218000000	3.2120000000	-0.9696000000
C	-4.8723000000	3.4417000000	-2.2261000000
C	-5.5800000000	3.0347000000	-3.5131000000
C	-7.0609000000	1.1797000000	-0.4405000000
C	-7.0182000000	0.4391000000	0.8951000000
C	-6.0599000000	-0.7441000000	0.8527000000
O	-5.5462000000	-4.8907000000	-1.3123000000
O	5.3258000000	4.3997000000	-2.2162000000
O	-2.7623000000	-5.2575000000	1.7954000000
O	3.6902000000	5.6932000000	1.3865000000
O	-5.0705000000	-3.8171000000	2.1977000000
O	5.9247000000	4.1039000000	1.4176000000
O	-8.5661000000	-2.1243000000	-2.3458000000
C	0.6973000000	-6.5481000000	0.1530000000
C	-0.7886000000	5.9345000000	0.1872000000
O	-4.2113000000	5.9707000000	1.1182000000
O	-5.9285000000	3.8710000000	1.4177000000
O	-4.6324000000	4.8971000000	-2.3939000000
O	-6.7841000000	3.8185000000	-3.7107000000
O	-5.7821000000	1.7683000000	-0.7244000000
O	-6.6224000000	1.3382000000	1.9658000000
O	-6.1550000000	-1.3370000000	2.1636000000
O	-5.4175000000	-2.7170000000	-0.3832000000
O	-7.4268000000	0.2340000000	-1.4698000000
C	-6.4559000000	-1.6891000000	-0.2764000000
C	-6.5442000000	-0.9488000000	-1.6175000000
C	-7.2065000000	-1.7745000000	-2.7139000000
C	5.8742000000	3.8350000000	-1.0072000000
C	-5.7737000000	-4.0849000000	-0.1320000000
C	5.4340000000	4.6883000000	0.1797000000
C	-4.9229000000	-4.6412000000	1.0097000000
C	3.9186000000	4.8363000000	0.2475000000
C	-3.4431000000	-4.7248000000	0.6363000000
C	3.4051000000	5.4183000000	-1.0654000000
C	-3.3002000000	-5.5736000000	-0.6196000000
C	3.8508000000	4.5654000000	-2.2604000000
C	-4.1338000000	-4.9730000000	-1.7602000000
C	3.5929000000	5.2362000000	-3.6042000000
C	-4.1590000000	-5.8380000000	-3.0108000000
C	1.2742000000	6.7033000000	-1.0796000000
C	-1.3309000000	-6.9058000000	-1.3067000000
C	0.3946000000	6.8976000000	0.1542000000
C	-0.5133000000	-7.4266000000	-0.1276000000

H	-5.0416000000	-0.3802000000	0.6605000000
H	-8.0280000000	0.0646000000	1.1044000000
H	-9.0606000000	-1.2904000000	-2.2000000000
H	-5.5448000000	-0.6197000000	-1.9222000000
H	-4.0031000000	6.6390000000	-1.5885000000
H	5.5194000000	4.6112000000	2.1537000000
H	-4.3886000000	-4.1127000000	2.8394000000
H	-5.8340000000	5.8424000000	-0.1845000000
H	2.7439000000	6.0003000000	1.4411000000
H	-2.2115000000	-6.0552000000	1.5629000000
H	3.4591000000	3.8515000000	0.4035000000
H	-3.0609000000	-3.7171000000	0.4382000000
H	-6.5257000000	4.7643000000	-3.7250000000
H	3.7788000000	6.4399000000	-1.2013000000
H	-3.6509000000	-6.5932000000	-0.4273000000
H	3.3744000000	3.5803000000	-2.2128000000
H	-3.7715000000	-3.9670000000	-1.9988000000
H	-4.8906000000	3.1489000000	-4.3597000000
H	-5.9026000000	1.9947000000	-3.4480000000
H	0.5592000000	6.7407000000	2.1198000000
H	-0.7328000000	-7.7371000000	1.8044000000
H	-5.6872000000	-2.2156000000	2.2126000000
H	2.0017000000	7.5108000000	-1.1874000000
H	-2.1224000000	-7.5967000000	-1.6058000000
H	-0.0004000000	7.9211000000	0.1254000000
H	-0.1706000000	-8.4406000000	-0.3675000000
H	-0.4224000000	4.9015000000	0.2500000000
H	0.3554000000	-5.5482000000	0.4501000000
H	-7.8463000000	1.9384000000	-0.4596000000
H	-6.4079000000	0.7840000000	2.7476000000
H	-3.9114000000	2.9261000000	-2.1237000000
H	-6.7319000000	3.6001000000	-1.1449000000
H	-4.1253000000	3.3951000000	0.4468000000
H	-6.1427000000	2.9442000000	1.7143000000
H	-4.7182000000	5.6759000000	1.9071000000
H	6.1648000000	2.0117000000	-3.4503000000
H	6.5807000000	0.3880000000	-4.0643000000
H	7.4233000000	1.8533000000	-0.7456000000
H	6.2042000000	2.4142000000	1.6522000000
H	6.4590000000	-3.0940000000	-2.9978000000
H	5.8482000000	-4.6375000000	-3.6542000000
H	7.2541000000	-5.6691000000	-2.0506000000
H	5.2628000000	-5.7074000000	1.1863000000
H	3.8806000000	-7.0003000000	-0.3590000000
H	5.2341000000	0.1297000000	-2.0138000000
H	8.6621000000	0.7151000000	-2.9642000000

H	5.1271000000	0.3514000000	0.6041000000
H	8.1280000000	-0.1836000000	0.7128000000
H	7.7230000000	-2.2319000000	-0.5615000000
H	6.7984000000	-0.6334000000	2.6210000000
H	4.2004000000	-3.7117000000	-2.0545000000
H	6.5238000000	-3.8923000000	-0.0705000000
H	3.5726000000	-3.2772000000	0.4470000000
H	-7.4227000000	-2.1591000000	-0.0627000000
H	-1.0803000000	8.1643000000	-3.5295000000
H	0.9128000000	-7.9067000000	-3.9274000000
H	-0.3457000000	4.8291000000	-2.3343000000
H	0.3891000000	-4.8824000000	-2.1024000000
H	-7.1845000000	-1.2127000000	-3.6563000000
H	-6.6761000000	-2.7193000000	-2.8436000000
H	6.9569000000	3.8582000000	-1.1489000000
H	-6.8428000000	-4.1747000000	0.0720000000
H	5.8745000000	5.6866000000	0.0633000000
H	-5.2831000000	-5.6534000000	1.2349000000
H	5.2699000000	6.2963000000	-3.5852000000
H	-5.4867000000	-7.1776000000	-2.3595000000
H	3.8782000000	4.5520000000	-4.4135000000
H	-4.8235000000	-5.3816000000	-3.7545000000
H	2.5358000000	5.4887000000	-3.6999000000
H	-3.1541000000	-5.9162000000	-3.4291000000
H	-2.4882000000	6.0926000000	1.3102000000
H	2.0610000000	-6.5902000000	1.6462000000
H	5.5504000000	-2.5080000000	1.9762000000
H	3.5401000000	-4.9193000000	2.6784000000
H	-2.0201000000	7.1029000000	-1.1509000000
H	2.0079000000	-7.3737000000	-1.3679000000
H	-0.7170000000	6.0179000000	-4.4709000000
H	0.8189000000	-5.5703000000	-4.4109000000
H	-2.2973000000	5.5908000000	-3.7594000000
H	2.3956000000	-5.4951000000	-3.5779000000
C	3.3832000000	1.5908000000	3.6718000000
C	0.7483000000	-2.7115000000	3.3777000000
C	2.7004000000	2.8554000000	3.6412000000
C	1.3611000000	2.9671000000	4.0456000000
C	0.7776000000	1.8145000000	4.5947000000
C	0.1945000000	3.9503000000	3.7124000000
C	1.4591000000	-1.6505000000	3.9614000000
C	-2.3274000000	3.0064000000	3.2225000000
C	2.9180000000	-1.1027000000	3.8451000000
C	-0.6836000000	-2.6747000000	3.2579000000
C	-1.4176000000	-1.5688000000	3.7183000000
C	2.7304000000	0.4261000000	4.1066000000

C	1.4357000000	0.5933000000	4.6238000000
C	-1.0671000000	3.0387000000	3.8381000000
C	-0.6525000000	1.8561000000	4.4706000000
C	0.6865000000	-0.6287000000	4.5360000000
C	-3.0777000000	1.7842000000	3.1326000000
C	-2.7989000000	-0.9352000000	3.3484000000
C	-0.6956000000	-0.5884000000	4.4162000000
C	-2.5734000000	0.5819000000	3.6546000000
C	-1.3760000000	0.6753000000	4.3812000000
H	4.3757000000	1.5423000000	3.2303000000
H	1.2718000000	-3.5277000000	2.8850000000
H	3.2064000000	3.6965000000	3.1737000000
H	0.2911000000	4.3702000000	2.7067000000
H	0.1566000000	4.7970000000	4.4133000000
H	-2.7034000000	3.8741000000	2.6882000000
H	3.3535000000	-1.3101000000	2.8630000000
H	3.5867000000	-1.5665000000	4.5845000000
H	-1.1681000000	-3.4762000000	2.7052000000
H	-3.9911000000	1.7989000000	2.5447000000
H	-3.6276000000	-1.3532000000	3.9365000000
H	-3.0461000000	-1.1005000000	2.2940000000

Table S6. Atomic coordinates for the DFT-optimized structure (B3LYP/6-31g(d,p)) of 1:1 sumanene@γCD inclusion complex labelled as **sumanene@γCD_4**.

	x	*y*	*z*
C	1.2717000000	6.7041000000	-1.0781000000
C	0.3918000000	6.8977000000	0.1557000000
C	-0.7913000000	5.9345000000	0.1881000000
C	-1.5984000000	6.0940000000	-1.0935000000
C	-0.7119000000	5.8630000000	-2.3240000000
C	-1.4057000000	6.2058000000	-3.6354000000
C	-4.0214000000	5.5882000000	-1.2886000000
C	-4.8614000000	5.3606000000	-0.0350000000
C	-5.0904000000	3.8812000000	0.2446000000
O	4.3137000000	6.4941000000	-3.6995000000
O	-4.5675000000	-7.2003000000	-2.7087000000
C	3.5911000000	5.2391000000	-3.6030000000
C	-4.1551000000	-5.8391000000	-3.0112000000
C	3.8491000000	4.5676000000	-2.2595000000
C	-4.1309000000	-4.9744000000	-1.7603000000
C	3.4030000000	5.4198000000	-1.0642000000
C	-3.2983000000	-5.5753000000	-0.6192000000
O	5.3242000000	4.4025000000	-2.2153000000
O	1.1821000000	6.7367000000	1.3652000000
O	-1.5163000000	6.2824000000	1.3867000000
O	0.4433000000	6.7927000000	-2.2592000000
O	-1.8652000000	7.5841000000	-3.6349000000
O	-2.6796000000	5.1087000000	-1.0763000000
O	-4.2142000000	5.9696000000	1.1179000000
O	-5.9307000000	3.8692000000	1.4168000000
O	-5.7824000000	1.7665000000	-0.7251000000
O	-4.6338000000	4.8958000000	-2.3944000000
C	-5.7228000000	3.2102000000	-0.9705000000
C	-4.8730000000	3.4403000000	-2.2266000000
C	-5.5800000000	3.0329000000	-3.5139000000
O	-5.5437000000	-4.8924000000	-1.3136000000
O	1.9388000000	5.4336000000	-1.0168000000
O	-1.8882000000	-5.6072000000	-0.9975000000
O	3.6876000000	5.6932000000	1.3879000000
O	-2.7629000000	-5.2606000000	1.7965000000
O	5.9228000000	4.1049000000	1.4184000000
O	-5.0710000000	-3.8197000000	2.1971000000
O	5.4164000000	2.4848000000	-0.8337000000
O	-6.7845000000	3.8160000000	-3.7119000000
O	-5.4162000000	-2.7189000000	-0.3837000000
O	8.1234000000	1.5150000000	-3.1631000000
O	-8.5645000000	-2.1273000000	-2.3469000000

C	-1.3281000000	-6.9068000000	-1.3054000000
C	-0.5098000000	-7.4272000000	-0.1266000000
C	0.7007000000	-6.5482000000	0.1532000000
C	1.5572000000	-6.4112000000	-1.1030000000
C	0.7224000000	-5.9093000000	-2.2877000000
C	1.4419000000	-6.0196000000	-3.6260000000
C	3.8926000000	-5.9098000000	-0.4354000000
C	4.3126000000	-5.2521000000	0.8798000000
C	4.5217000000	-3.7495000000	0.7255000000
O	7.1661000000	-0.6853000000	-1.7475000000
O	-7.4263000000	0.2314000000	-1.4707000000
O	6.4916000000	1.4651000000	1.7460000000
O	-6.1546000000	-1.3391000000	2.1629000000
O	6.7950000000	-1.2785000000	1.8796000000
O	-6.6225000000	1.3360000000	1.9650000000
O	7.4431000000	-4.7291000000	-2.2683000000
C	3.9165000000	4.8372000000	0.2485000000
C	-3.4424000000	-4.7270000000	0.6369000000
O	-1.3375000000	-7.4880000000	1.0740000000
O	1.3897000000	-7.2008000000	1.2420000000
O	-0.4667000000	-6.7852000000	-2.4475000000
O	1.7549000000	-7.4012000000	-3.9357000000
O	2.6085000000	-5.4213000000	-0.8561000000
O	3.3240000000	-5.4966000000	1.9178000000
O	4.9422000000	-3.2912000000	2.0292000000
O	5.6416000000	-2.0479000000	-0.5646000000
O	4.9000000000	-5.6076000000	-1.4283000000
C	5.5477000000	-3.4950000000	-0.3712000000
C	5.1277000000	-4.1653000000	-1.6854000000
C	6.2121000000	-4.1269000000	-2.7531000000
C	-7.0610000000	1.1773000000	-0.4413000000
C	6.9542000000	-1.4544000000	-0.5467000000
C	-7.0180000000	0.4367000000	0.8943000000
C	7.0911000000	-0.5339000000	0.6635000000
C	-6.0593000000	-0.7462000000	0.8520000000
C	6.1747000000	0.6809000000	0.5754000000
C	-6.4549000000	-1.6913000000	-0.2771000000
C	6.4169000000	1.4206000000	-0.7351000000
C	-6.5432000000	-0.9510000000	-1.6183000000
C	6.2686000000	0.4813000000	-1.9385000000
C	-7.2050000000	-1.7770000000	-2.7148000000
C	6.7322000000	1.1069000000	-3.2474000000
C	-5.7723000000	-4.0869000000	-0.1332000000
C	5.8727000000	3.8374000000	-1.0065000000
C	-4.9224000000	-4.6435000000	1.0091000000
C	5.4320000000	4.6899000000	0.1807000000

H	3.5729000000	-3.2754000000	0.4446000000
H	5.2646000000	-5.7036000000	1.1866000000
H	7.2578000000	-5.6663000000	-2.0493000000
H	4.2021000000	-3.7114000000	-2.0560000000
H	-2.1194000000	-7.5981000000	-1.6042000000
H	-6.4083000000	0.7820000000	2.7469000000
H	6.8008000000	-0.6325000000	2.6188000000
H	-0.1668000000	-8.4410000000	-0.3665000000
H	-5.6870000000	-2.2179000000	2.2118000000
H	6.2036000000	2.4152000000	1.6520000000
H	-5.0411000000	-0.3820000000	0.6601000000
H	5.1275000000	0.3525000000	0.6032000000
H	0.9148000000	-7.9067000000	-3.9274000000
H	-7.4217000000	-2.1615000000	-0.0635000000
H	7.4225000000	1.8560000000	-0.7468000000
H	-5.5439000000	-0.6215000000	-1.9227000000
H	5.2334000000	0.1323000000	-2.0150000000
H	0.8195000000	-5.5702000000	-4.4107000000
H	2.3966000000	-5.4944000000	-3.5785000000
H	-4.3900000000	-4.1157000000	2.8396000000
H	5.5172000000	4.6116000000	2.1547000000
H	5.5513000000	-2.5046000000	1.9741000000
H	-6.8416000000	-4.1771000000	0.0698000000
H	6.9554000000	3.8610000000	-1.1481000000
H	-5.2827000000	-5.6558000000	1.2337000000
H	5.8721000000	5.6883000000	0.0649000000
H	-3.0598000000	-3.7193000000	0.4395000000
H	3.4573000000	3.8521000000	0.4038000000
H	3.8833000000	-6.9987000000	-0.3581000000
H	3.5412000000	-4.9155000000	2.6777000000
H	0.3906000000	-4.8826000000	-2.1020000000
H	2.0109000000	-7.3732000000	-1.3682000000
H	0.3585000000	-5.5484000000	0.4505000000
H	2.0644000000	-6.5890000000	1.6463000000
H	-0.7285000000	-7.7373000000	1.8055000000
H	-5.9020000000	1.9927000000	-3.4488000000
H	-4.8903000000	3.1474000000	-4.3602000000
H	-6.7330000000	3.5978000000	-1.1460000000
H	-6.1443000000	2.9423000000	1.7135000000
H	-2.2987000000	5.5910000000	-3.7591000000
H	-0.7183000000	6.0191000000	-4.4700000000
H	-1.0829000000	8.1650000000	-3.5282000000
H	-0.0033000000	7.9212000000	0.1272000000
H	1.9990000000	7.5119000000	-1.1854000000
H	-3.9118000000	2.9252000000	-2.1238000000
H	-6.5265000000	4.7619000000	-3.7261000000

H	-4.1269000000	3.3941000000	0.4465000000
H	-5.8365000000	5.8407000000	-0.1853000000
H	-4.0054000000	6.6380000000	-1.5889000000
H	-4.7212000000	5.6744000000	1.9067000000
H	-0.3470000000	4.8299000000	-2.3336000000
H	-2.0228000000	7.1027000000	-1.1501000000
H	-0.4251000000	4.9014000000	0.2511000000
H	6.5248000000	-3.8892000000	-0.0708000000
H	-5.4835000000	-7.1785000000	-2.3614000000
H	5.2677000000	6.2999000000	-3.5834000000
H	-3.7685000000	-3.9683000000	-1.9983000000
H	3.3731000000	3.5823000000	-2.2125000000
H	5.8512000000	-4.6378000000	-3.6542000000
H	6.4603000000	-3.0930000000	-2.9993000000
H	-7.8467000000	1.9357000000	-0.4605000000
H	7.7236000000	-2.2292000000	-0.5649000000
H	-8.0277000000	0.0619000000	1.1035000000
H	8.1288000000	-0.1811000000	0.7100000000
H	-9.0594000000	-1.2936000000	-2.2011000000
H	8.6608000000	0.7191000000	-2.9664000000
H	-7.1830000000	-1.2152000000	-3.6572000000
H	6.5790000000	0.3922000000	-4.0659000000
H	-6.6742000000	-2.7215000000	-2.8444000000
H	6.1631000000	2.0154000000	-3.4509000000
H	-2.2097000000	-6.0565000000	1.5636000000
H	2.7412000000	6.0001000000	1.4425000000
H	-2.4913000000	6.0923000000	1.3105000000
H	0.5564000000	6.7403000000	2.1213000000
H	-3.6491000000	-6.5950000000	-0.4276000000
H	3.7763000000	6.4415000000	-1.1995000000
H	-4.8188000000	-5.3823000000	-3.7554000000
H	3.8768000000	4.5554000000	-4.4127000000
H	-3.1497000000	-5.9173000000	-3.4286000000
H	2.5339000000	5.4911000000	-3.6988000000
C	-2.3263000000	3.0066000000	3.2205000000
C	2.7012000000	2.8533000000	3.6426000000
C	-3.0771000000	1.7848000000	3.1308000000
C	-2.5738000000	0.5825000000	3.6539000000
C	-1.3768000000	0.6757000000	4.3811000000
C	-2.7999000000	-0.9346000000	3.3485000000
C	1.3618000000	2.9659000000	4.0461000000
C	-0.6854000000	-2.6752000000	3.2603000000
C	0.1958000000	3.9495000000	3.7116000000
C	3.3833000000	1.5884000000	3.6743000000
C	2.7297000000	0.4242000000	4.1094000000
C	-1.0663000000	3.0387000000	3.8368000000

C	-0.6528000000	1.8563000000	4.4703000000
C	-1.4191000000	-1.5688000000	3.7196000000
C	-0.6970000000	-0.5883000000	4.4173000000
C	0.7773000000	1.8139000000	4.5954000000
C	0.7465000000	-2.7126000000	3.3809000000
C	2.9166000000	-1.1048000000	3.8489000000
C	1.4347000000	0.5924000000	4.6257000000
C	1.4574000000	-1.6518000000	3.9646000000
C	0.6850000000	-0.6292000000	4.5381000000
H	-2.7014000000	3.8742000000	2.6854000000
H	3.2080000000	3.6938000000	3.1750000000
H	-3.9902000000	1.7996000000	2.5424000000
H	-3.0466000000	-1.1005000000	2.2940000000
H	-3.6292000000	-1.3519000000	3.9363000000
H	-1.1699000000	-3.4767000000	2.7076000000
H	0.2935000000	4.3688000000	2.7057000000
H	0.1579000000	4.7966000000	4.4120000000
H	4.3761000000	1.5391000000	3.2333000000
H	1.2699000000	-3.5293000000	2.8889000000
H	3.5846000000	-1.5684000000	4.5892000000
H	3.3527000000	-1.3132000000	2.8673000000

Table S7. Atomic coordinates for the DFT-optimized structure (B3LYP/6-31g(d,p)) of 1:1 sumanene@γCD non-inclusion complex labelled as **sumanene@γCD_5**.

	x	*y*	*z*
C	6.8014000000	-4.5062000000	1.2436000000
C	6.7350000000	-5.5220000000	0.0986000000
C	5.3605000000	-5.5129000000	-0.5590000000
C	4.2834000000	-5.7663000000	0.4953000000
C	4.4142000000	-4.7532000000	1.6431000000
C	3.5306000000	-5.0944000000	2.8407000000
C	2.3424000000	-6.7957000000	-0.5228000000
C	1.8474000000	-6.6376000000	-1.9650000000
C	0.7331000000	-5.6020000000	-2.0654000000
O	8.3515000000	-2.3251000000	3.8855000000
O	-5.6419000000	2.4737000000	1.9100000000
C	7.1611000000	-1.7413000000	3.3803000000
C	-4.4274000000	1.7377000000	1.8774000000
C	7.3518000000	-1.2032000000	1.9651000000
C	-4.1614000000	1.1399000000	0.4991000000
C	7.7845000000	-2.2891000000	0.9664000000
C	-4.1970000000	2.1962000000	-0.6188000000
O	8.3880000000	-0.2031000000	2.0789000000
O	7.7478000000	-5.2363000000	-0.8599000000
O	5.4056000000	-6.5067000000	-1.5702000000
O	5.7612000000	-4.7690000000	2.1654000000
O	3.8902000000	-6.3347000000	3.4215000000
O	2.9880000000	-5.6174000000	-0.0964000000
O	2.9325000000	-6.2905000000	-2.8187000000
O	0.3193000000	-5.6038000000	-3.4185000000
O	-1.3495000000	-4.9116000000	-1.1264000000
O	1.2445000000	-7.1150000000	0.3113000000
C	-0.3837000000	-5.9690000000	-1.0938000000
C	0.1702000000	-6.1525000000	0.3282000000
C	-0.8593000000	-6.7199000000	1.3012000000
O	-5.2088000000	0.1680000000	0.2946000000
O	6.7032000000	-3.1982000000	0.7257000000
O	-3.0714000000	3.0754000000	-0.5096000000
O	8.7610000000	-2.5869000000	-1.2721000000
O	-4.3537000000	2.4361000000	-3.0604000000
O	9.5368000000	0.0739000000	-1.3711000000
O	-5.2183000000	-0.1742000000	-3.3398000000
O	7.6511000000	1.1786000000	0.3570000000
O	-1.3558000000	-7.9766000000	0.8655000000
O	-4.0056000000	-1.2893000000	-1.0676000000
O	8.8723000000	3.6637000000	2.7438000000
O	-5.6765000000	-3.8487000000	1.1090000000

C	-3.3023000000	4.3809000000	-0.0225000000
C	-2.9787000000	5.4089000000	-1.1113000000
C	-1.4878000000	5.4228000000	-1.4256000000
C	-0.6913000000	5.6689000000	-0.1440000000
C	-1.0773000000	4.6356000000	0.9253000000
C	-0.5011000000	4.9581000000	2.3019000000
C	1.4232000000	6.7286000000	-0.6713000000
C	2.2384000000	6.5908000000	-1.9628000000
C	3.3411000000	5.5494000000	-1.8142000000
O	7.0054000000	4.7624000000	0.9756000000
O	-3.4678000000	-4.8269000000	-0.1634000000
O	8.1477000000	2.4278000000	-2.2358000000
O	-3.9460000000	-2.6788000000	-3.6545000000
O	6.7935000000	4.8159000000	-2.6667000000
O	-2.4767000000	-5.0272000000	-3.6683000000
O	4.6839000000	7.9390000000	1.5095000000
C	8.1733000000	-1.6685000000	-0.3715000000
C	-4.1429000000	1.5346000000	-1.9899000000
O	-3.7309000000	5.1227000000	-2.2865000000
O	-1.3067000000	6.4312000000	-2.4060000000
O	-2.5087000000	4.6381000000	1.1182000000
O	-0.9968000000	6.1862000000	2.8036000000
O	0.7089000000	5.5405000000	-0.4145000000
O	1.3839000000	6.2639000000	-3.0531000000
O	4.0525000000	5.5569000000	-3.0375000000
O	5.1459000000	4.8353000000	-0.4273000000
O	2.2988000000	7.0448000000	0.3940000000
C	4.2098000000	5.9040000000	-0.6113000000
C	3.3496000000	6.0898000000	0.6502000000
C	4.1332000000	6.6694000000	1.8248000000
C	-2.7075000000	-5.2613000000	-1.2749000000
C	6.5123000000	5.1560000000	-0.2909000000
C	-3.2734000000	-4.6359000000	-2.5536000000
C	7.3183000000	4.4576000000	-1.3914000000
C	-3.3439000000	-3.1164000000	-2.4523000000
C	7.3166000000	2.9427000000	-1.2136000000
C	-4.1197000000	-2.7100000000	-1.2020000000
C	7.7960000000	2.5935000000	0.1914000000
C	-3.5370000000	-3.3992000000	0.0433000000
C	6.9714000000	3.3449000000	1.2492000000
C	-4.4094000000	-3.2301000000	1.2840000000
C	7.5352000000	3.1955000000	2.6590000000
C	-5.1825000000	-0.5180000000	-0.9423000000
C	8.7446000000	0.4535000000	0.8789000000
C	-5.2589000000	0.4999000000	-2.0839000000
C	9.2189000000	-0.5814000000	-0.1459000000

H	2.8788000000	4.5669000000	-1.6304000000
H	2.7159000000	7.5646000000	-2.1459000000
H	3.9354000000	8.4953000000	1.2491000000
H	2.9166000000	5.1207000000	0.9322000000
H	-4.3369000000	4.4800000000	0.3193000000
H	-2.7700000000	-4.4622000000	-4.4011000000
H	7.2233000000	4.2181000000	-3.2988000000
H	-3.2607000000	6.3998000000	-0.7255000000
H	-4.2218000000	-1.7450000000	-3.5575000000
H	8.4619000000	1.5377000000	-1.9808000000
H	-2.3191000000	-2.7232000000	-2.3560000000
H	6.2837000000	2.5758000000	-1.3246000000
H	-1.9619000000	6.1095000000	2.7838000000
H	-5.1719000000	-2.9994000000	-1.3089000000
H	8.8484000000	2.8810000000	0.2985000000
H	-2.5326000000	-3.0004000000	0.2357000000
H	5.9345000000	2.9848000000	1.2233000000
H	-0.7257000000	4.1230000000	2.9829000000
H	0.5842000000	5.0564000000	2.2234000000
H	-5.0708000000	0.5224000000	-3.9994000000
H	9.6691000000	-0.6376000000	-2.0174000000
H	4.9187000000	5.1222000000	-2.9091000000
H	-6.0682000000	-1.1608000000	-0.9376000000
H	9.5572000000	1.1339000000	1.1512000000
H	-6.2175000000	1.0271000000	-1.9833000000
H	10.1222000000	-1.0590000000	0.2604000000
H	-3.1722000000	1.0241000000	-2.0927000000
H	7.2727000000	-1.2053000000	-0.8064000000
H	0.7360000000	7.5767000000	-0.7451000000
H	1.9656000000	5.9823000000	-3.7760000000
H	-0.7461000000	3.6392000000	0.6045000000
H	-0.9091000000	6.6691000000	0.2505000000
H	-1.2019000000	4.4371000000	-1.8209000000
H	-0.4180000000	6.3220000000	-2.7993000000
H	-3.3156000000	5.6641000000	-2.9787000000
H	-1.7154000000	-6.0435000000	1.3623000000
H	-0.3985000000	-6.7919000000	2.2971000000
H	-0.8455000000	-6.9104000000	-1.4134000000
H	-0.5764000000	-5.2170000000	-3.4826000000
H	2.4936000000	-5.1833000000	2.5080000000
H	3.5915000000	-4.2730000000	3.5706000000
H	4.8315000000	-6.2631000000	3.6372000000
H	6.9061000000	-6.5194000000	0.5298000000
H	7.7270000000	-4.6208000000	1.8162000000
H	0.5368000000	-5.1857000000	0.6985000000
H	-0.5817000000	-8.5506000000	0.7719000000

H	1.1355000000	-4.6182000000	-1.7761000000
H	1.4315000000	-7.6092000000	-2.2700000000
H	3.0220000000	-7.6499000000	-0.4480000000
H	2.5374000000	-6.0150000000	-3.6600000000
H	4.1705000000	-3.7496000000	1.2704000000
H	4.3938000000	-6.7745000000	0.9129000000
H	5.1847000000	-4.5188000000	-0.9954000000
H	4.7435000000	6.8401000000	-0.8129000000
H	-6.3469000000	1.8741000000	1.6233000000
H	9.0297000000	-1.6356000000	3.8365000000
H	-3.1831000000	0.6411000000	0.4948000000
H	6.4216000000	-0.7379000000	1.6139000000
H	3.4672000000	6.7246000000	2.6983000000
H	4.9701000000	6.0096000000	2.0671000000
H	-2.8197000000	-6.3497000000	-1.2909000000
H	6.6553000000	6.2402000000	-0.3324000000
H	-4.2976000000	-5.0159000000	-2.6807000000
H	8.3580000000	4.8069000000	-1.3092000000
H	-5.4927000000	-4.7689000000	0.8694000000
H	8.8525000000	4.5864000000	2.4510000000
H	-3.8790000000	-3.6468000000	2.1528000000
H	6.8758000000	3.7283000000	3.3599000000
H	-4.5922000000	-2.1680000000	1.4641000000
H	7.5505000000	2.1382000000	2.9351000000
H	-3.9662000000	3.3050000000	-2.8343000000
H	8.2631000000	-3.4288000000	-1.2516000000
H	4.6330000000	-6.3851000000	-2.1569000000
H	7.4993000000	-5.7543000000	-1.6435000000
H	-5.1292000000	2.7658000000	-0.5333000000
H	8.6438000000	-2.8267000000	1.3839000000
H	-4.4285000000	0.9253000000	2.6179000000
H	6.8090000000	-0.9215000000	4.0240000000
H	-3.6277000000	2.4387000000	2.1298000000
H	6.4001000000	-2.5257000000	3.3720000000
C	-8.7388000000	3.5931000000	0.7334000000
C	-8.2489000000	0.2595000000	4.4618000000
C	-8.9274000000	3.1440000000	-0.6140000000
C	-9.4882000000	1.8913000000	-0.8889000000
C	-9.9633000000	1.1718000000	0.2134000000
C	-9.4133000000	0.8929000000	-2.0791000000
C	-8.8774000000	1.1889000000	3.6244000000
C	-9.0664000000	-1.7740000000	-1.6194000000
C	-8.6625000000	2.6964000000	3.3091000000
C	-8.2832000000	-1.1419000000	4.1738000000
C	-8.9448000000	-1.6363000000	3.0435000000
C	-9.1132000000	2.7984000000	1.8237000000

C	-9.7841000000	1.6084000000	1.5174000000
C	-9.5581000000	-0.4860000000	-1.3750000000
C	-10.0038000000	-0.2322000000	-0.0735000000
C	-9.6423000000	0.6569000000	2.5804000000
C	-8.9130000000	-2.7228000000	-0.5571000000
C	-8.7963000000	-2.9047000000	2.1585000000
C	-9.6758000000	-0.7016000000	2.3018000000
C	-9.2510000000	-2.3986000000	0.7621000000
C	-9.8584000000	-1.1528000000	0.9538000000
H	-8.1835000000	4.5154000000	0.8826000000
H	-7.6135000000	0.5886000000	5.2805000000
H	-8.5090000000	3.7549000000	-1.4103000000
H	-8.4685000000	0.9749000000	-2.6255000000
H	-10.2144000000	1.0671000000	-2.8104000000
H	-8.6731000000	-2.0442000000	-2.5966000000
H	-7.6142000000	2.9864000000	3.4274000000
H	-9.2571000000	3.3462000000	3.9654000000
H	-7.6705000000	-1.7957000000	4.7898000000
H	-8.4046000000	-3.6560000000	-0.7851000000
H	-9.4204000000	-3.7341000000	2.5194000000
H	-7.7635000000	-3.2654000000	2.1269000000

S6. Analyzes of mechanochemically-obtained inclusion complexes

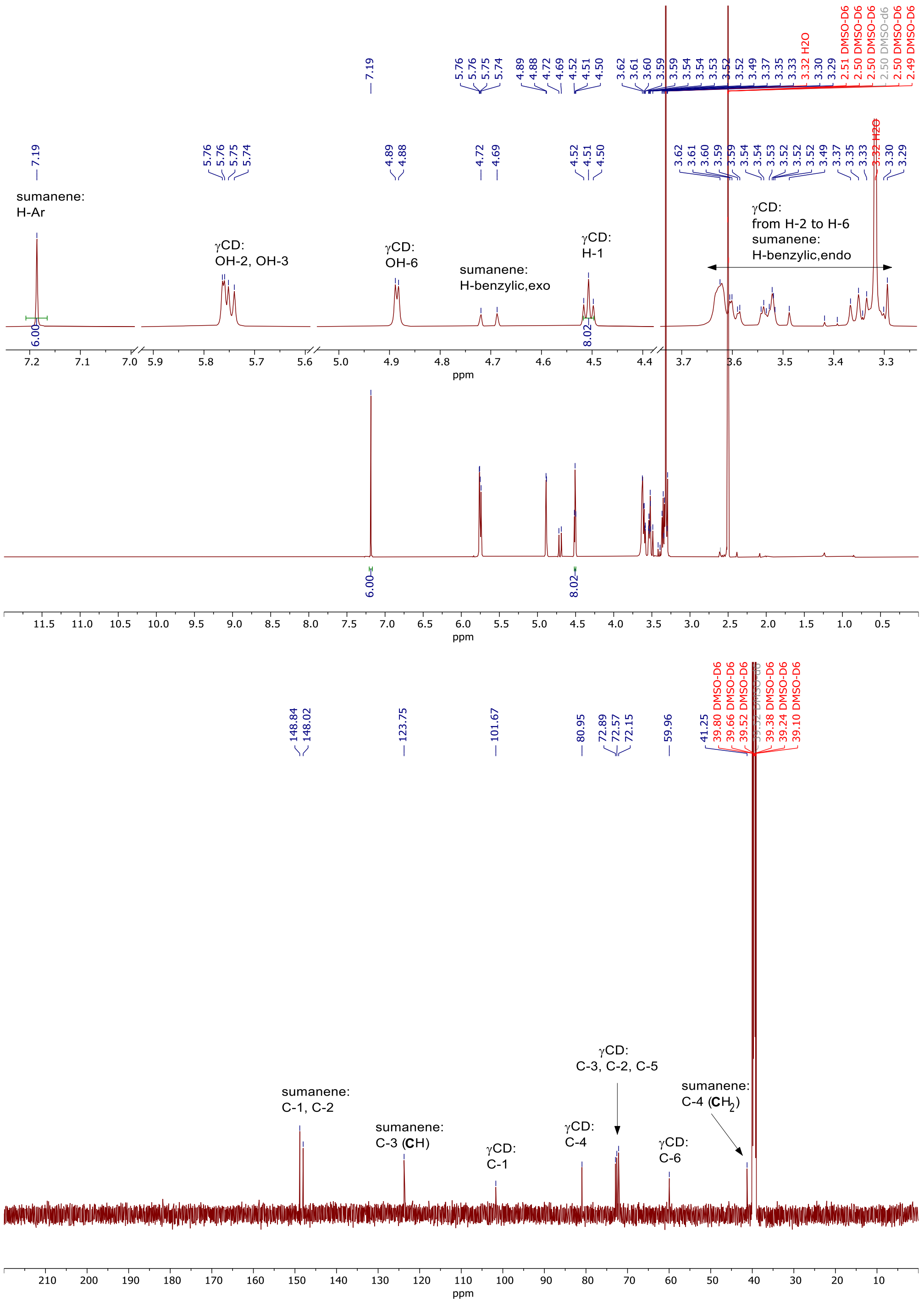

Fig. S47. (top) ^{1}H NMR spectrum (600 MHz, DMSO-d_6) of mechanochemically-prepared sumanene@γCD inclusion complex. Selected signals from which 1:1 stoichiometry of the molecules in the complex could be confirmed are integrated. Sumanene[27] and γCD[28] signals are also marked; (bottom) $\{^{1}H\}^{13}C$ NMR spectrum (151 MHz, DMSO-d_6) of mechanochemically-prepared sumanene@γCD inclusion complex. Sumanene[27] and γCD[28] signals are also marked.

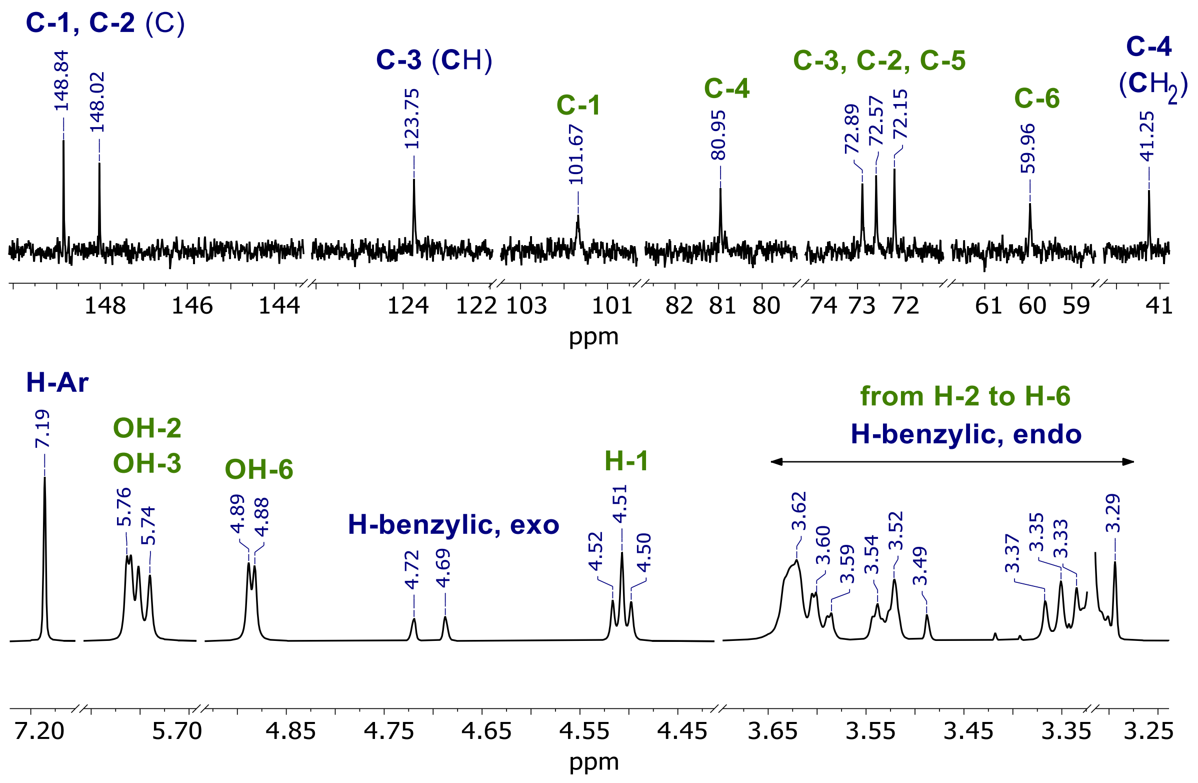

Fig. S48. Selected insets of ^{1}H (600 MHz, DMSO-d_6) and {^{1}H}^{13}C NMR (151 MHz, DMSO-d_6) spectra of mechanochemically-prepared sumanene@γCD complex. Signals labels in blue and green correspond to the signals coming from sumanene and γCD, respectively. In the ^{1}H NMR spectrum, for the clarity of the image (*i*) selected peaks were marked and (*ii*) residual HOD signal was cut off.

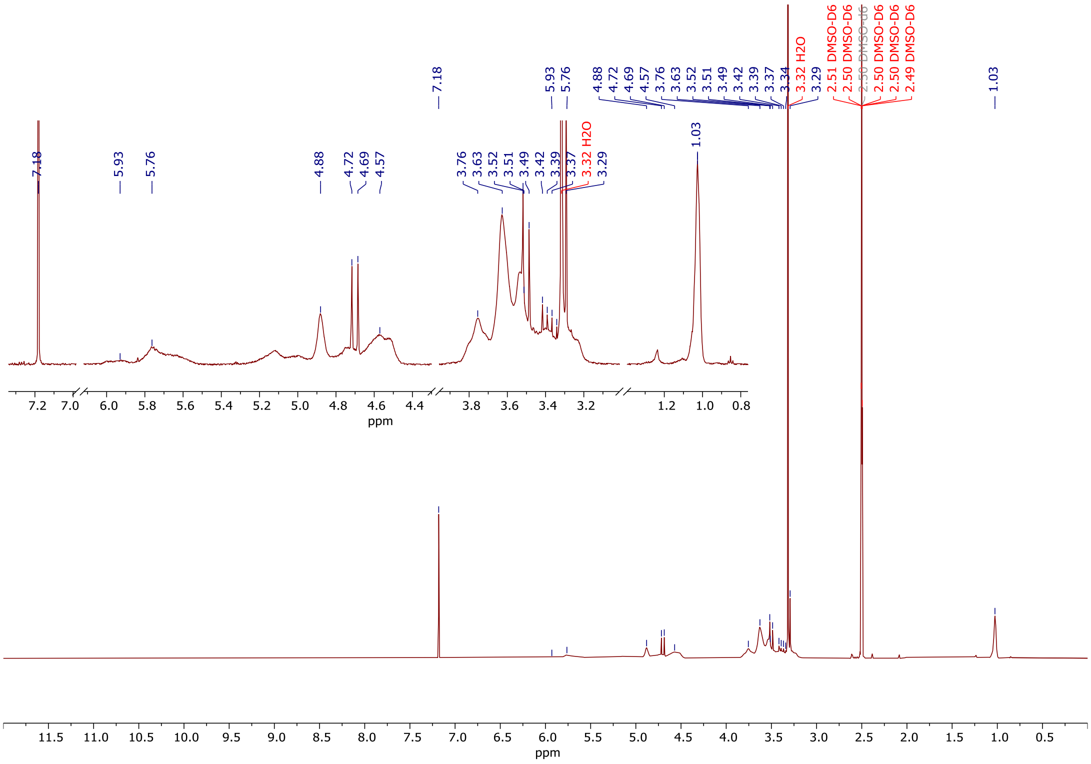

Fig. S49. ^{1}H NMR spectrum (600 MHz, DMSO-d_6) of mechanochemically-prepared sumanene@HP-γCD inclusion complex.

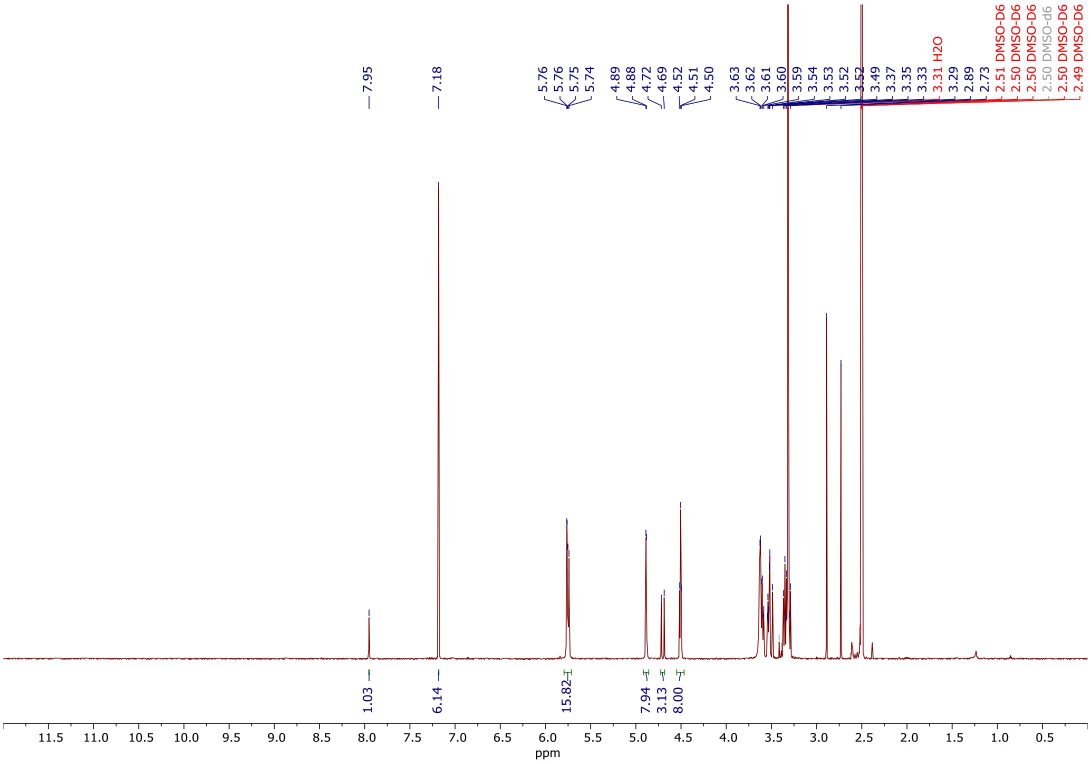

Fig. S50. ^{1}H NMR spectrum (600 MHz, DMSO-d_6) of mechanochemically-prepared sumanene@γCD inclusion complex (C = 1.6815 mM) for the purpose of qNMR analysis.

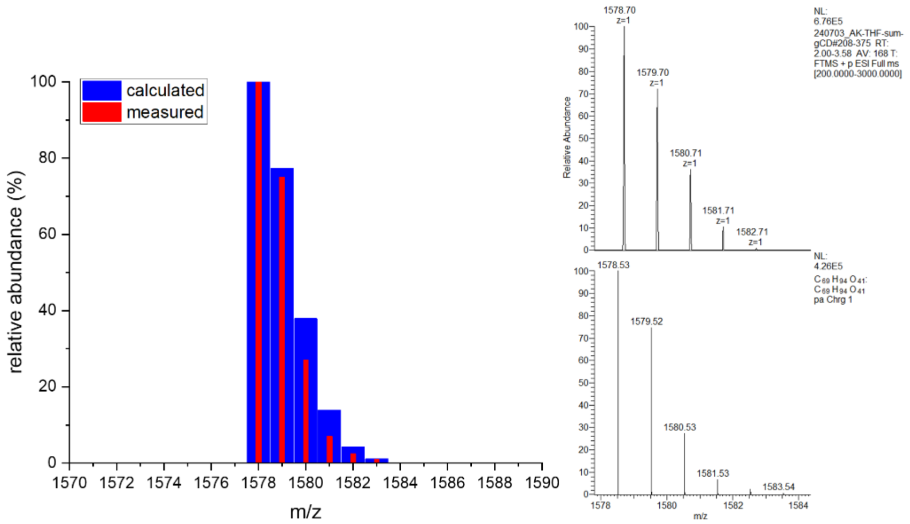

Fig. S51. ESI-MS (TOF) spectrum and presentation of isotope pattern of mechanochemically-prepared sumanene@γCD inclusion complex (stacked experimental and simulated spectra). The simulated isotope pattern for hydrated sumanene@γCD inclusion complex ($C_{69}H_{92}O_{40}{\cdot}H_2O$; $[M]^+$ calcd. for $C_{69}H_{94}O_{41}$ 1578.5271) is shown in blue (generated with Isotope Pattern Calculator v4.0, www: https://yanjunhua.tripod.com/pattern.htm). The experimental data are presented in red. The raw ESI-MS (TOF) spectrum image from the experiment is also presented (top: measured, bottom: simulated).

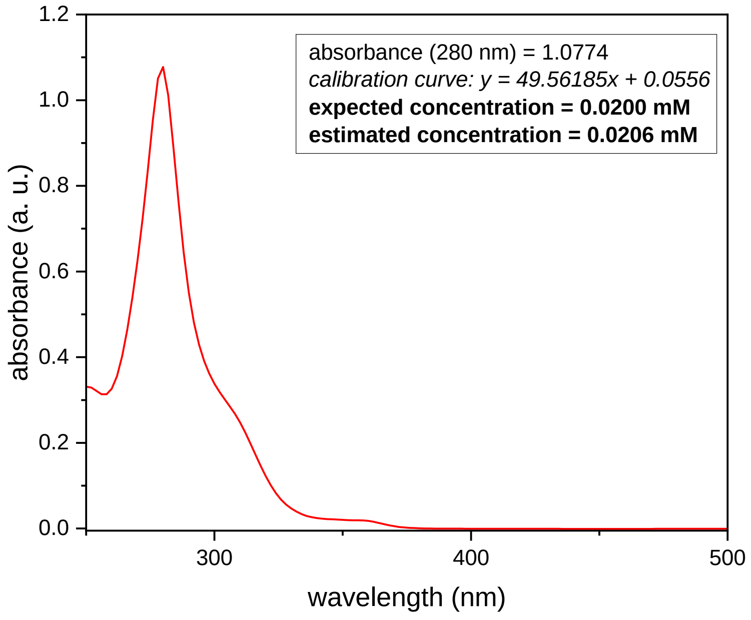

Fig. S52. UV-vis spectra (DMSO:H_2O = 90:10 *vol*/vol) of sumanene@γCD complex at the anticipated concentration of 2×10^{-5} M. Sumanene content in the sample calculated from the calibration curve (from phase solubility studies experiments; Fig. S29) is also presented.

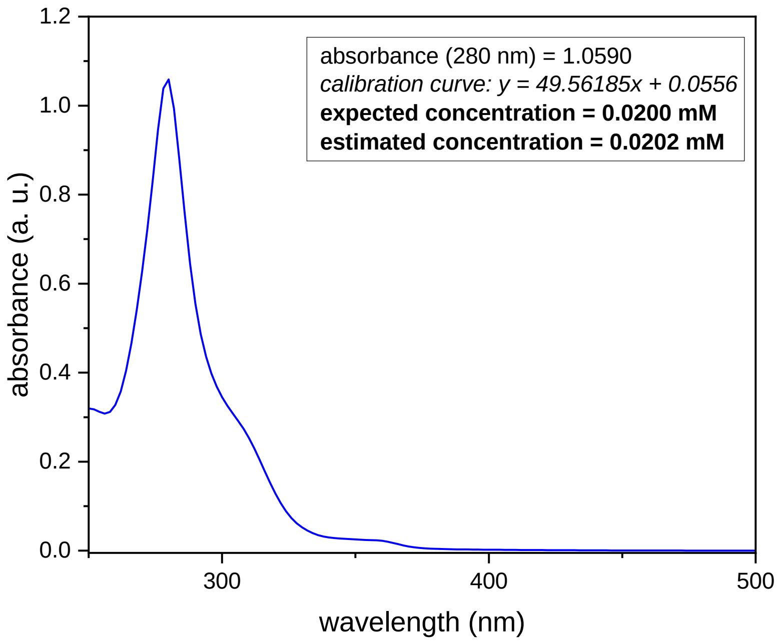

Fig. S53. UV-vis spectra (DMSO:H_2O = 90:10 *vol*/vol) of sumanene@HP-γCD complex at the anticipated concentration of 2 × 10^{-5} M. Sumanene content in the sample calculated from the calibration curve (from phase solubility studies experiments; Fig. S29) is also presented.

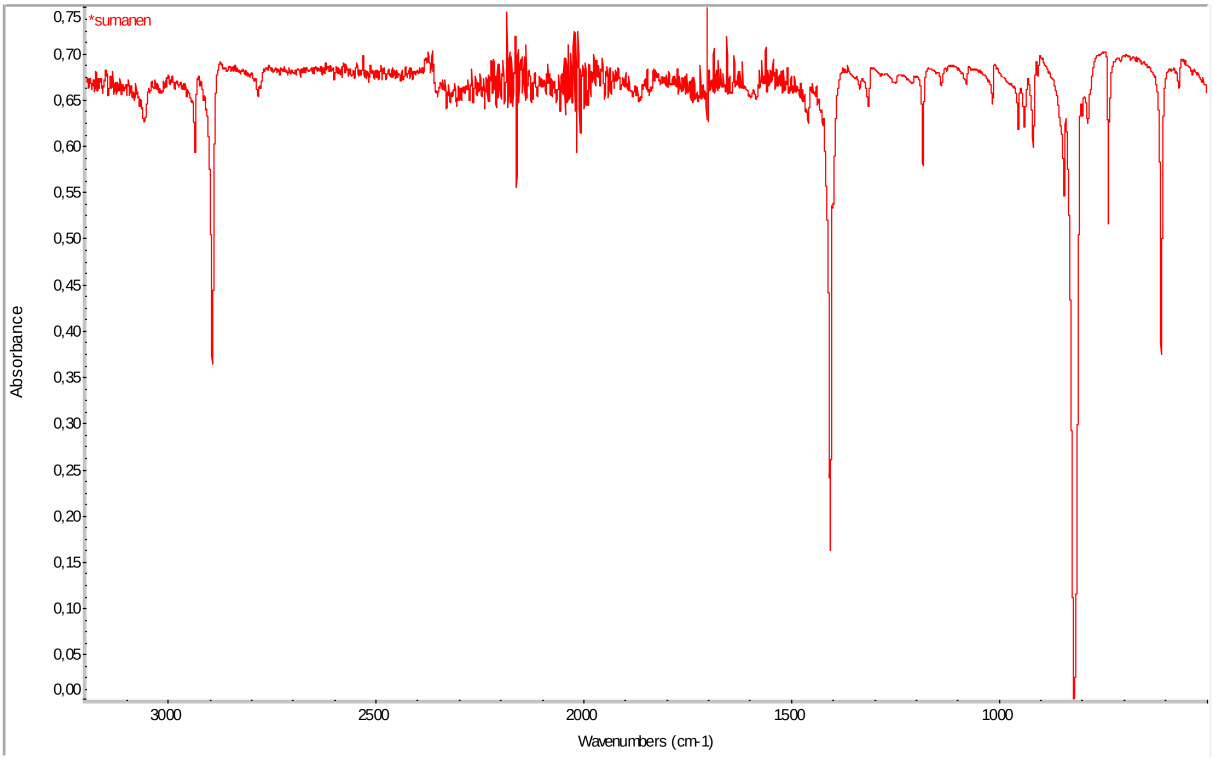

Fig. S54. FT-IR (ATR) spectrum native sumanene.

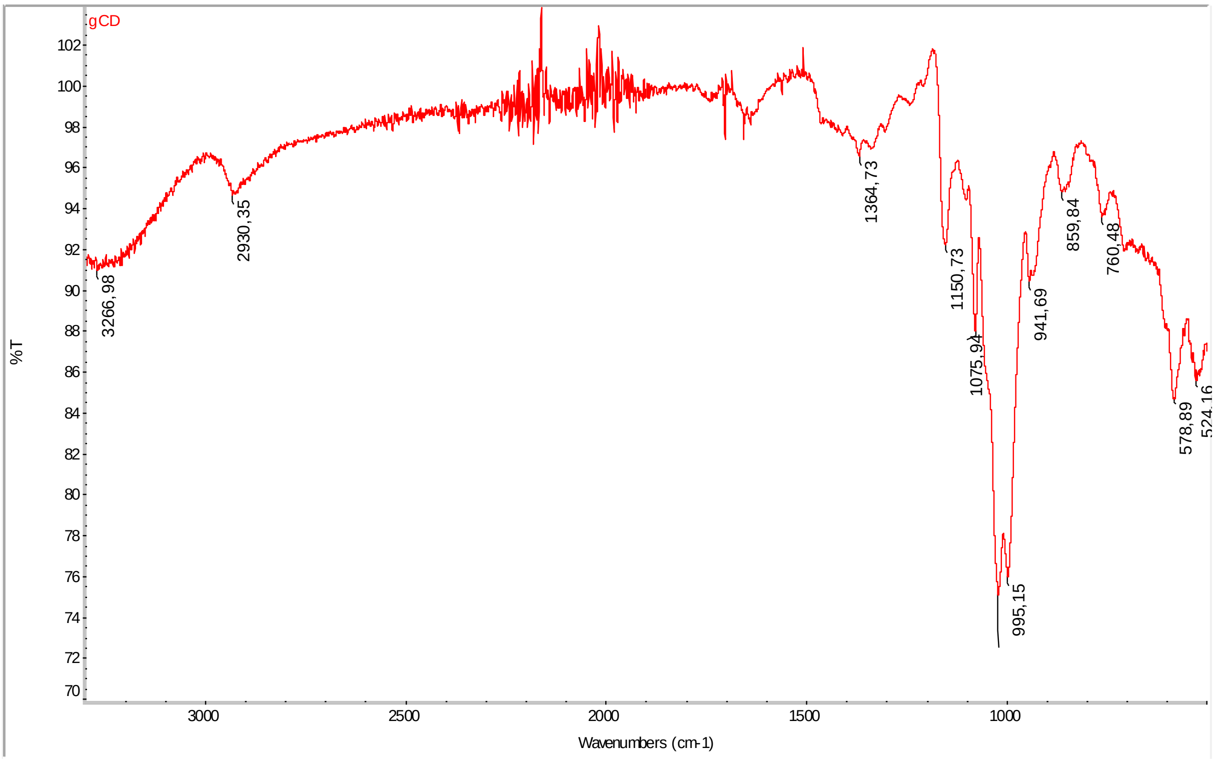

Fig. S55. FT-IR (ATR) spectrum native γCD.

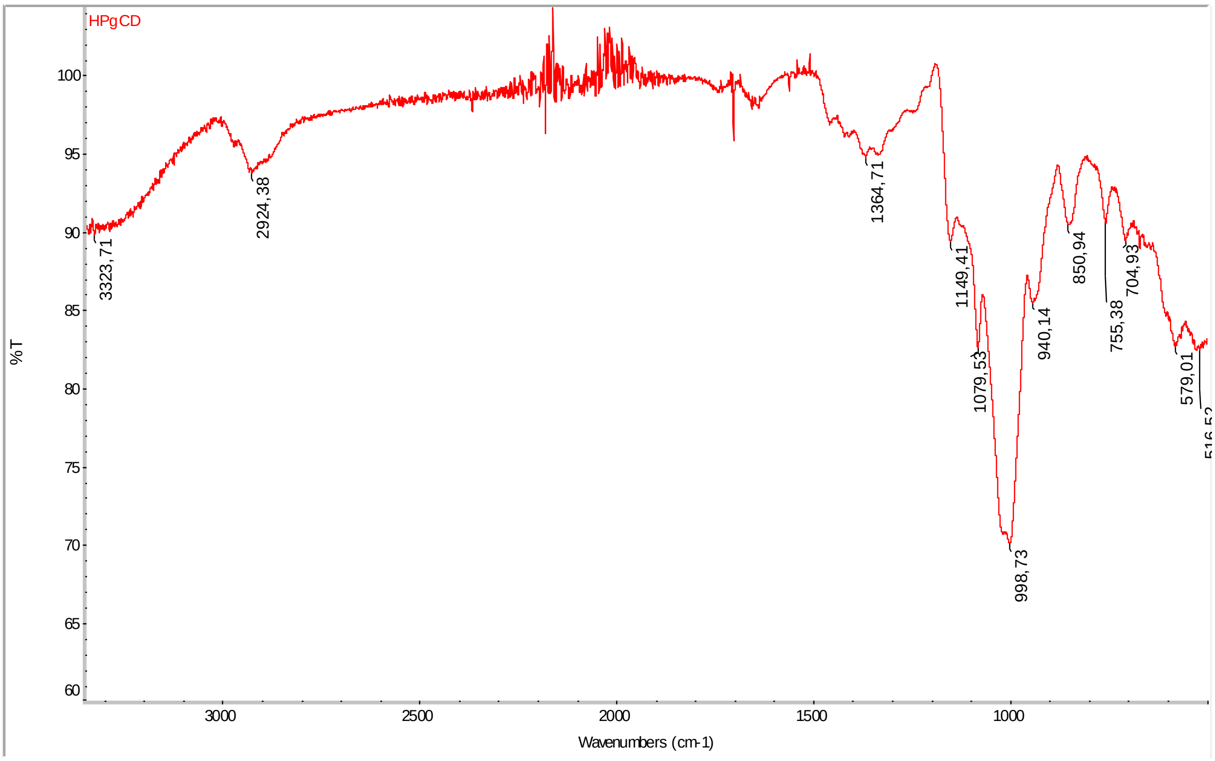

Fig. S56. FT-IR (ATR) spectrum native HP-γCD.

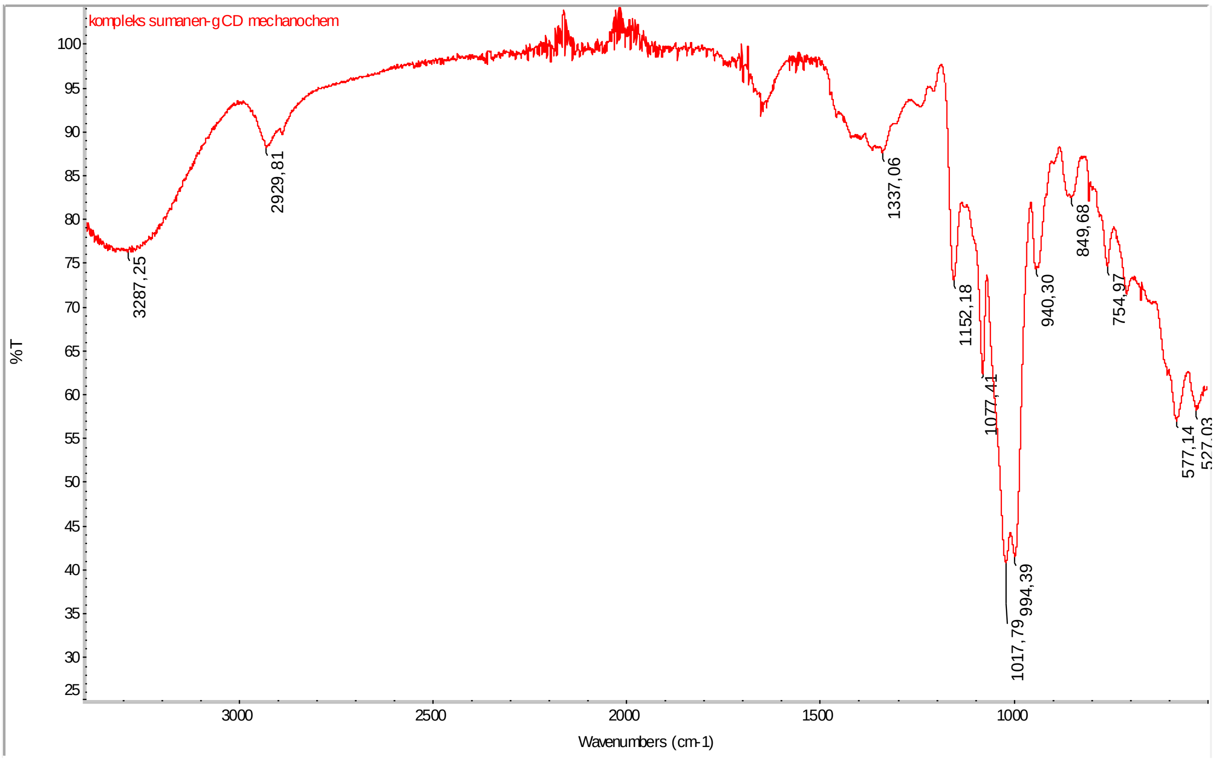

Fig. S57. FT-IR (ATR) spectrum of mechanochemically-prepared sumanene@γCD inclusion complex.

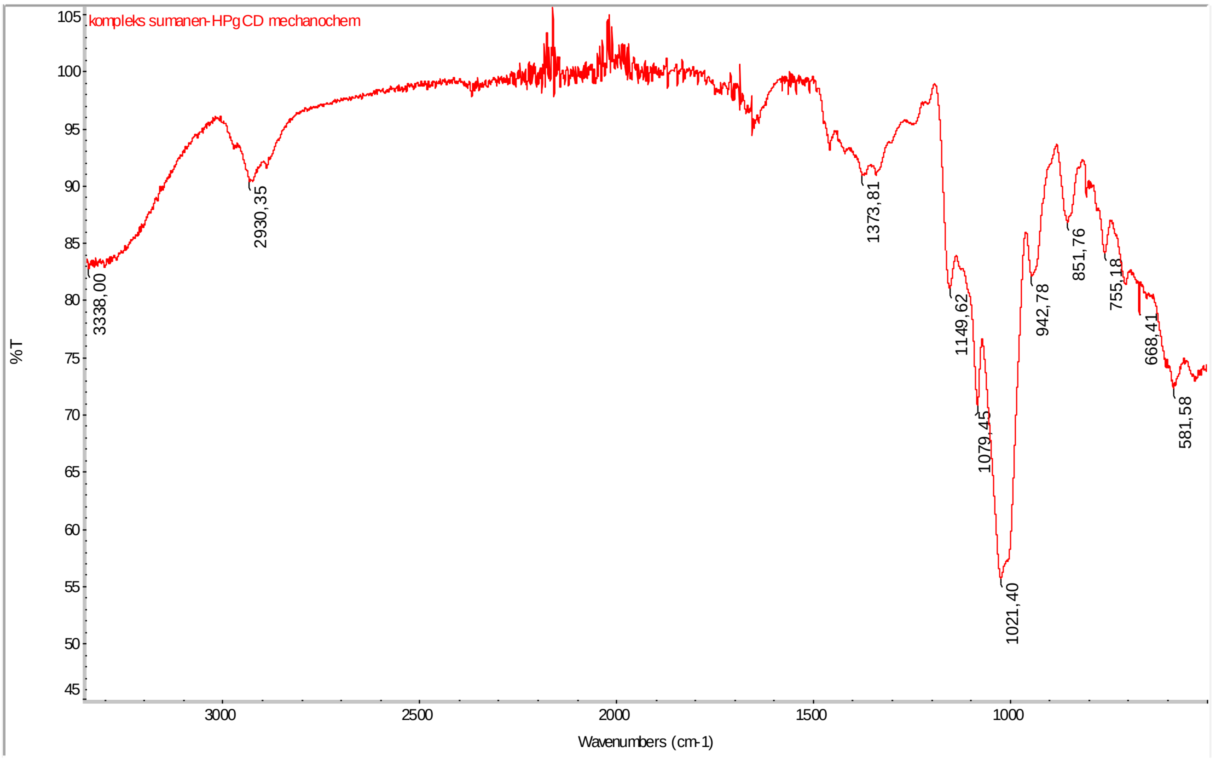

Fig. S58. FT-IR (ATR) spectrum of mechanochemically-prepared sumanene@HP-γCD inclusion complex.

S7. *In sillico* modeling of pharmacokinetic (ADME-Tox) properties

Table S8. List of predicted values of pharmacokinetic properties for native sumanene and sumanene@γCD complex (legend: A – absorption, D – distribution, M – metabolism, E – excretion, Tox – toxicity).

Prop.	Model name	Unit	Predicted value for native **sumanene**	Predicted value for **sumanene@γCD complex**
-	Log P	-	4.8588	−12.5476
A	Water solubility	Numeric (log mol/L)	−7.618	−2.892
	Caco2 permeability	Numeric (log Papp in 10^{-6} cm/s)	1.057	−0.321
	Intestinal absorption (human)	Numeric (% Absorbed)	100	70.043
	Skin Permeability	Numeric (log Kp)	−2.735	−2.735
	P-glycoprotein substrate	Categorical (Yes/No)	Yes	Yes
	P-glycoprotein I inhibitor	Categorical (Yes/No)	No	Yes
	P-glycoprotein II inhibitor	Categorical (Yes/No)	Yes	Yes
D	VDss (human)	Numeric (log L/kg)	−0.089	0.011
	Fraction unbound (human)	Numeric (Fu)	0.264	0.381
	BBB permeability	Numeric (log BB)	0.913	−2.722
	CNS permeability	Numeric (log PS)	−1.354	−9.868
M	CYP2D6 substrate	Categorical (Yes/No)	Yes	No
	CYP3A4 substrate	Categorical (Yes/No)	Yes	No
	CYP1A2 inhibitior	Categorical (Yes/No)	Yes	No
	CYP2C19 inhibitior	Categorical (Yes/No)	Yes	No
	CYP2C9 inhibitior	Categorical (Yes/No)	No	No
	CYP2D6 inhibitior	Categorical (Yes/No)	No	No
	CYP3A4 inhibitior	Categorical (Yes/No)	No	No
E	Total Clearance	Numeric (log ml/min/kg)	−0.128	0.236
	Renal OCT2 substrate	Categorical (Yes/No)	No	No
Tox	AMES toxicity	Categorical (Yes/No)	Yes	Yes
	Max. tolerated dose (human)	Numeric (log mg/kg/day)	0.598	0.438
	hERG I inhibitor	Categorical (Yes/No)	No	No
	hERG II inhibitor	Categorical (Yes/No)	Yes	No
	Oral Rat Acute Toxicity (LD50)	Numeric (mol/kg)	2.629	2.482
	Oral Rat Chronic Toxicity (LOAEL)	Numeric (log mg/kg_bw/day)	0.595	6.094
	Hepatotoxicity	Categorical (Yes/No)	Yes	No
	Skin Sensitisation	Categorical (Yes/No)	No	No
	T.Pyriformis toxicity	Numeric (log mg/L)	0.285	0.285
	Minnow toxicity	Numeric (log mM)	−2.012	39.75

Table S9. List of predicted values of pharmacokinetic properties for sumanene@HP-γCD complex (legend: A – absorption, D – distribution, M – metabolism, E – excretion, Tox – toxicity).

Prop.	Model name	Unit	Predicted value for **sumanene@HP-γCD complex**
-	Log P	-	−11.7519
A	Water solubility	Numeric (log mol/L)	−2.892
	Caco2 permeability	Numeric (log Papp in 10^{-6} cm/s)	−0.59
	Intestinal absorption (human)	Numeric (% Absorbed)	43.508
	Skin Permeability	Numeric (log Kp)	−2.735
	P-glycoprotein substrate	Categorical (Yes/No)	Yes
	P-glycoprotein I inhibitor	Categorical (Yes/No)	Yes
	P-glycoprotein II inhibitor	Categorical (Yes/No)	Yes
D	VDss (human)	Numeric (log L/kg)	0.011
	Fraction unbound (human)	Numeric (Fu)	0.381
	BBB permeability	Numeric (log BB)	−3.247
	CNS permeability	Numeric (log PS)	−10.607
M	CYP2D6 substrate	Categorical (Yes/No)	No
	CYP3A4 substrate	Categorical (Yes/No)	No
	CYP1A2 inhibitior	Categorical (Yes/No)	No
	CYP2C19 inhibitior	Categorical (Yes/No)	No
	CYP2C9 inhibitior	Categorical (Yes/No)	No
	CYP2D6 inhibitior	Categorical (Yes/No)	No
	CYP3A4 inhibitior	Categorical (Yes/No)	No
E	Total Clearance	Numeric (log ml/min/kg)	0.273
	Renal OCT2 substrate	Categorical (Yes/No)	No
Tox	AMES toxicity	Categorical (Yes/No)	Yes
	Max. tolerated dose (human)	Numeric (log mg/kg/day)	0.438
	hERG I inhibitor	Categorical (Yes/No)	No
	hERG II inhibitor	Categorical (Yes/No)	Yes
	Oral Rat Acute Toxicity (LD50)	Numeric (mol/kg)	2.482
	Oral Rat Chronic Toxicity (LOAEL)	Numeric (log mg/kg_bw/day)	3.427
	Hepatotoxicity	Categorical (Yes/No)	No
	Skin Sensitisation	Categorical (Yes/No)	No
	T.Pyriformis toxicity	Numeric (log mg/L)	0.285
	Minnow toxicity	Numeric (log mM)	43.614

S8. Supporting references

1. Sakurai, H., Daiko, T. & Hirao, T. A Synthesis of Sumanene, a Fullerene Fragment. *Science* **301**, 1878–1878 (2003).
2. Wu, D. H., Chen, A. D. & Johnson, C. S. An Improved Diffusion-Ordered Spectroscopy Experiment Incorporating Bipolar-Gradient Pulses. *J. Magn. Res. A* **115**, 260–264 (1995).
3. Jerschow, A. & Müller, N. Suppression of Convection Artifacts in Stimulated-Echo Diffusion Experiments. Double-Stimulated-Echo Experiments. *J. Magn. Res.* **125**, 372–375 (1997).
4. Nilsson, M. The DOSY Toolbox: A new tool for processing PFG NMR diffusion data. *J. Magn. Res.* **200**, 296–302 (2009).
5. Benesi, H. A. & Hildebrand, J. H. A Spectrophotometric Investigation of the Interaction of Iodine with Aromatic Hydrocarbons. *J. Am. Chem. Soc.* **71**, 2703–2707 (1949).
6. http://supramolecular.org/.
7. Thordarson, P. Determining association constants from titration experiments in supramolecular chemistry. *Chem. Soc. Rev.* **40**, 1305–1323 (2011).
8. Brynn Hibbert, D. & Thordarson, P. The death of the Job plot, transparency, open science and online tools, uncertainty estimation methods and other developments in supramolecular chemistry data analysis. *Chem. Commun.* **52**, 12792–12805 (2016).
9. Huang, C. Y. Determination of binding stoichiometry by the continuous variation method: The job plot. in *Methods in Enzymology* vol. 87 509–525 (Elsevier, 1982).
10. Renny, J. S., Tomasevich, L. L., Tallmadge, E. H. & Collum, D. B. Method of Continuous Variations: Applications of Job Plots to the Study of Molecular Associations in Organometallic Chemistry. *Angew. Chem. Int. Ed.* **52**, 11998–12013 (2013).
11. Avram, L. & Cohen, Y. Complexation in Pseudorotaxanes Based on α-Cyclodextrin and Different α,ω-Diaminoalkanes by NMR Diffusion Measurements. *J. Org. Chem.* **67**, 2639–2644 (2002).
12. Kasprzak, A., Koszytkowska-Stawińska, M., Nowicka, A. M., Buchowicz, W. & Poplawska, M. Supramolecular Interactions between β-Cyclodextrin and the Nucleobase Derivatives of Ferrocene. *J. Org. Chem.* **84**, 15900–15914 (2019).
13. Jullian, C., Miranda, S., Zapata-Torres, G., Mendizábal, F. & Olea-Azar, C. Studies of inclusion complexes of natural and modified cyclodextrin with (+)catechin by NMR and molecular modeling. *Bioorg. Med. Chem.* **15**, 3217–3224 (2007).
14. (a) Bakkour, Y. *et al.* Formation of Cyclodextrin Inclusion Complexes with Doxycyclin-Hyclate: NMR Investigation of Their Characterisation and Stability. *J. Incl. Phenom. Macrocycl. Chem.* **54**, 109–114 (2006), (b) Viel, S. *et al*. Pulsed Field Gradient Magic Angle Spinning NMR Self-Diffusion Measurements in Liquids. J. Magn. Res. **190** (1), 113–123 (**2008**).
15. Higuchi, T. & Connors, K. A. *Phase Solubility Techniques*. (1965).
16. Frisch, M. J. *et al.* Gaussian 16, Revision C.01, Gaussian, Inc., Wallingford CT. (2016).
17. Becke, A. D. Density-functional thermochemistry. III. The role of exact exchange. *J. Chem. Phys.* **98**, 5648–5652 (1993).
18. Ditchfield, R., Hehre, W. J. & Pople, J. A. Self-Consistent Molecular-Orbital Methods. IX. An Extended Gaussian-Type Basis for Molecular-Orbital Studies of Organic Molecules. *J. Chem. Phys* **54**, 724–728 (1971).
19. Hanwell, M. D. *et al.* Avogadro: an advanced semantic chemical editor, visualization, and analysis platform. *J. Cheminform.* **4**, 17 (2012).
20. Dennington, R., Keith, T. A. & Millam, J. M. GaussView, Version 6.1, Semichem Inc., Shawnee Mission, KS. (2016).

21. Davies, S. R. *et al.* Purity assessment of organic calibration standards using a combination of quantitative NMR and mass balance. Anal. Bioanal. Chem. **407**, 3103–3113 (2015).
22. Pauli, G. F., Gödecke, T., Jaki, B. U. & Lankin, D. C. Quantitative ^{1}H NMR. Development and Potential of an Analytical Method: An Update. *J. Nat. Prod.* **75**, 834–851 (2012).
23. Pauli, G. F. *et al.* Importance of Purity Evaluation and the Potential of Quantitative ^{1}H NMR as a Purity Assay: Miniperspective. *J. Med. Chem.* **57**, 9220–9231 (2014).
24. https://biosig.lab.uq.edu.au/pkcsm/, access date: 2024-08-09.
25. Pires, D. E. V., Blundell, T. L. & Ascher, D. B. pkCSM: Predicting Small-Molecule Pharmacokinetic and Toxicity Properties Using Graph-Based Signatures. *J. Med. Chem.* **58**, 4066–4072 (2015).
26. Dulsat, J., López-Nieto, B., Estrada-Tejedor, R. & Borrell, J. I. Evaluation of Free Online ADMET Tools for Academic or Small Biotech Environments. *Molecules* **28**, 776 (2023).
27. Higashibayashi, S., Pandit, P., Haruki, R., Adachi, S. & Kumai, R. Redox-Dependent Transformation of a Hydrazinobuckybowl between Curved and Planar Geometries. *Angew. Chem. In.t Ed.* **55**, 10830–10834 (2016).
28. Schneider, H.-J., Hacket, F., Rüdiger, V. & Ikeda, H. NMR Studies of Cyclodextrins and Cyclodextrin Complexes. *Chem. Rev.* **98**, 1755–1786 (1998).
29. Joshi, H., Sreejith, S., Dey, R. & Stuparu, M. C. Host–guest interaction between corannulene and γ-cyclodextrin: mass spectrometric evidence of a 1 : 1 inclusion complex formation. *RSC Adv.* **6**, 110001–110003 (2016).
